# Downregulation of ZFP36L1 contributes to methotrexate resistance in osteosarcoma through enhanced NHEJ DNA repair mechanisms

**DOI:** 10.1038/s41419-025-08217-4

**Published:** 2025-11-24

**Authors:** Jiahao Zhuang, Mengjun Ma, Biao Yang, Yinliang Liu, Rujia Mi, Wen Yang, Yixuan Lu, Haoye Yu, Wangchang Wu, Yihui Song, Peng Wang, Hongyu Li

**Affiliations:** 1 Department of Orthopedics, The Eighth Affiliated Hospital of Sun Yat-sen University, Shenzhen, China; 2 Center for Biotherapy, The Eighth Affiliated Hospital of Sun Yat-sen University, Shenzhen, China

**Keywords:** Cancer therapeutic resistance, Tumour-suppressor proteins

## Abstract

Chemotherapy resistance poses a significant challenge in the treatment of osteosarcoma. While DNA damage repair mechanisms play a crucial role in this resistance, effective intervention strategies remain limited. This study elucidates an intrinsic DNA damage repair mechanism in osteosarcoma cells treated with methotrexate (MTX) that can be effectively disrupted through the overexpression of ZFP36L1. Our findings indicate that ZFP36L1 expression is downregulated in MTX-resistant osteosarcoma cells. By overexpressing ZFP36L1 in osteosarcoma cell lines, we observed an increase in sensitivity to MTX. Further investigation revealed that the overexpression of ZFP36L1 reduces the efficiency of the DNA damage repair process, particularly by inhibiting the DCLRE1C-mediated non-homologous end joining (NHEJ) pathway. Through analysis of the 3′ untranslated region (3′UTR) of DCLRE1C mRNA, we identified 8 potential AU-rich elements (AREs) that bind to ZFP36L1. We demonstrated that ZFP36L1 directly interacts with DCLRE1C mRNA, leading to its degradation. In summary, decreased ZFP36L1 expression serves as an inherent mechanism enabling osteosarcoma to develop resistance to MTX therapy. These results highlight ZFP36L1 as a promising therapeutic target for overcoming MTX chemoresistance in osteosarcoma.

## Introduction

Osteosarcoma (OS) is the most common primary malignant bone tumor affecting children and adolescents. While the combination of neoadjuvant chemotherapy and surgery has improved survival rates to over 60% in recent decades, the prognosis for patients with relapsed or metastatic OS remains poor, with survival rates around 20% [1, 2]. A major factor contributing to this disparity is the development of chemoresistance, underscoring the urgent need to investigate its underlying mechanisms and identify potential therapeutic targets.

Zinc Finger Protein 36 Like 1 (ZFP36L1) features two conserved CCCH-type zinc finger domains that bind to adenylate-uridylate (AU)-rich elements (AREs) within the 3′ untranslated regions (3′UTRs) of mRNA [3]. Functionally, ZFP36L1 acts as an RNA-binding protein, capturing target mRNAs and recruiting the CCR4-NOT deadenylase complex to mediate ARE-dependent RNA decay [4]. Its downregulation has been implicated in tumor progression in various cancers [5, 6]. In bladder cancer, ZFP36L1 destabilizes oncogenic mRNAs associated with cell cycle control and hypoxic signaling pathways [7]. Our earlier research demonstrated that ZFP36L1 suppresses OS lung metastasis by inhibiting the TGF-β signaling pathway [8]. In this study, we reveal that ZFP36L1 also plays a pivotal role in regulating methotrexate (MTX) resistance in OS.

Double-strand breaks (DSBs) are among the most severe forms of DNA damage induced by chemotherapeutic agents [9]. To preserve genomic stability and develop resistance, cancer cells enhance their DSB repair mechanisms, primarily through non-homologous end joining (NHEJ) and homologous recombination (HR) pathways [10, 11]. As the dominant repair pathway, NHEJ directly rejoins DNA ends via a process involving the Ku70/80 heterodimer, which recruits DNA-PKcs to initiate the repair response. DNA-PKcs interacts with DCLRE1C, an endonuclease critical for processing damaged DNA ends, followed by recruitment of DNA polymerases and a ligase complex to complete the repair [12]. Dysregulation of NHEJ components has been associated with cancer resistance [13, 14], yet the post-transcriptional regulation of these core factors remains poorly understood, presenting a promising target for synthetic lethality approaches.

This study identifies a novel function of ZFP36L1 in OS. We demonstrate that ZFP36L1 is downregulated in MTX-resistant OS cells. Overexpression of ZFP36L1 reverses MTX resistance by impairing DNA repair, specifically inhibiting NHEJ-mediated DSBs repair. Mechanistically, ZFP36L1 promotes the decay of DCLRE1C mRNA, thereby reducing DCLRE1C expression in an ARE-dependent manner. Our findings suggest that ZFP36L1-targeted therapy holds potential as a synthetic lethality strategy for treating OS.

## Materials and methods

### Study approval

All paraffin-embedded osteosarcoma tissues for IHC assays were obtained from patients with OS at the Eighth Affiliated Hospital, Sun Yat-sen University (Shenzhen, China) and Sun Yat-Sen Memorial Hospital (Guangzhou, China). Informed consent was obtained from all patients. Approval of this study was obtained from the Ethics Committee of The Eighth Affiliated Hospital, Sun Yat-sen University (Shenzhen, China). All the methods were performed in accordance with the relevant guidelines and regulations.

### Animals

All animal experiments were approved by the Sun Yat-sen University Laboratory Animal Care and Use Committee (Guangzhou, China). Six- to eight-week-old female BALB/c nude mice were used for experiments in this study. To establish the subcutaneous tumor model, mice were randomly divided into groups, each consisting of 5 mice, and 143B cells (1 × 10^6^) were subcutaneously injected into the flanks of BALB/c nude mice and grew for 2 weeks. For drug treatment, MTX (5 mg/kg/week) (MCE, USA) was administered by intraperitoneal injection. The mice were euthanized after drug treatment, and the tumors were harvested. After photography, the tumor was divided into two parts for protein extraction and paraffin embedding, respectively.

To establish the metastatic OS xenograft model, mice were randomly divided into groups, each consisting of 5 mice, and 143B cells (2 × 10^6^) stably expressing luciferase, were intravenously injected into the tail vein of the mice, followed by growth for 2 weeks. After administration with MTX (5 mg/kg/week) by intraperitoneal injection for 3 weeks, the growth of lung metastases was measured by detecting the luciferase activity with a Xenogen IVIS 200 imaging system. The mice were euthanized, and the lungs were harvested for Hematoxylin-Eosin (HE) and Immunohistochemistry (IHC) assays to determine tumor burden.

### Immunohistochemistry (IHC) staining

The paraffin was sliced into 4 μm sections, followed by deparaffinization and hydration with xylene and gradient alcohol, respectively. After eliminating endogenous peroxidase activity with H_2_O_2_, the sections were incubated with citrate buffer for antigen retrieval and blocked with goat serum. Next, the sections were incubated with primary antibody against ZFP36L1 (Solarbio, #K110803P), c-PARP (Santa Cruz, #sc-56196), c-caspase3 (CST, #9661S), DCLRE1C (Santa Cruz, #sc-518193), and γ-H2AX (CST, #2577S) overnight at 4 °C. After washing with PBS, immunostaining was conducted with the SP Rabbit & Mouse HRP Kit (ComWin Biotech, China). The images were captured by microscopy.

The staining scores were determined by the percentage of positive cells and staining intensity. In brief, the percentage of positive cells was graded as follows: 0-1, 0–10% positive cells; 1–2, 10–50% positive cells; and 2–3, >50% positive cells. The staining intensity was graded as follows: 0, no staining; 0–1, light brown; 1–2, brown; and 2–3, dark brown. The comprehensive score = the percentage score × the staining intensity score.

### Cell transfection

The osteosarcoma cell lines (143B and U2OS) and 293 T cells were obtained from American Type Culture Collection (ATCC, Manassas, VA, USA) and cultured according to ATCC protocols. Regular Mycoplasma testing was performed on all cell lines to confirm their authenticity.

### Cell transfection

Knockdown and overexpression cell lines were generated through lentiviral transduction. Briefly, psPAX2, pMD2G, and target plasmids were transfected into HEK293T cells with Lipofectamine 3000 (ThermoFisher, USA). After 24 h, the culture medium was collected, filtered, and subsequently used for lentiviral transduction twice a day. The stable knockdown and overexpression cells were selected with the corresponding drugs after lentiviral transduction. Plasmids (pLKO.1-shZFP36L1, pLKO.1-shDCLRE1C, pLVX-ZFP36L1-FLAG, and pLVX-DCLRE1C) were constructed, and siRNAs were synthesized by Fubio Biotechnology (Suzhou, China) or Igebio Biotechnology (Guangzhou, China). The target sequences of shRNA and siRNA were as follows: shZFP36L1#1: 5’- GCT CGC GAG ACA GCC GCT TCC -3’; shZFP36L1#2: 5’- GCT TCC GAG ACC GCT CCT TCT -3’; shDCLRE1C#1: 5’- GAT CCT CTG CCA ATA CCT TTA -3’; shDCLRE1C#2: 5’- TAT GGA TAA AGT TGT CGA AAT -3’; siZFP36#1: 5’- GAT CCG ACC CTG ATG AAT ATG -3’; siZFP36#2: 5’- ATC TGT CTC CTA GAA TCT TAT -3’; siZFP36L2#1: 5’- GCC ACC TTG CTA AAC CTA TAA -3’; and siZFP36L2#2: 5’- GCT CAA CAA GGA GAA CAA ATT -3’.

### Western blotting (WB)

Cells or tissues were lysed by radio immunoprecipitation assay (RIPA) buffer (CWBio, China) containing phenylmethanesulfonyl fluoride (PMSF) (Beyotime, China) and phosphatase inhibitor (CWBio, China), and total protein was boiled with 5x sodium dodecyl sulfate-polyacrylamide gel electrophoresis (SDS-PAGE) loading buffer (Beyotime, China). Bicinchoninic acid (BCA) protein assay kit was used to determine the protein concentration. Equal amounts of protein samples were separated on 8-12% SDS‒PAGE gel and transferred to 0.22 μm or 0.45 μm polyvinylidene fluoride (PVDF) membranes (Merck Millipore, USA). The membranes were incubated with the selected primary antibodies against ZFP36L1 (Solarbio, #K110803P), GAPDH (CST, #2118S), c-caspase3 (CST, #9661S), caspase3 (CST, #9662S), γ-H2AX (CST, #2577S), H2AX (CST, #2595S), DCLRE1C (Santa Cruz, #sc-518193), XRCC1 (CST, # 2735S), XPC (CST, # 12701T), ZFP36 (proteintech, # 12737-1-AP), and ZFP36L2 (CST, # 85891T) at 4 °C overnight, followed by blocked with 5% skim milk. After incubation with horseradish peroxidase (HRP)-conjugated antibody (Boster, China), the HRP activity was visualized with Chemiluminescent HRP Substrate (Merck Millipore, USA) and detected with an ECL detection system. The bands were quantified using ImageJ software.

### RNA extraction and quantitative real‑time PCR (qRT‑PCR)

Total RNA was extracted from cells by using an RNA-Quick Purification Kit (ES Science, China) according to the manufacturer’s instructions. The RNA concentration was detected with a NanoDrop spectrophotometer (ThermoFisher, USA). Reverse transcription was performed with an equal amount of RNA with Evo M-MLV RT Premix for q-PCR kit (Accurate Biology, China). qRT-PCR was conducted using SYBR Green Pro Taq HS q-PCR Kit (Accurate Biology, China). The relative mRNA expression was calculated by the 2^-ΔΔCt^ method. GAPDH was used as a control. All primers used in this study are listed in Table S1.

### Cell viability assay

143B and U2OS cells were seeded into the 96-well plates at a number of 1000 or 2500 cells/well, respectively. After culturing for the indicated days, 10 μl of CCK-8 solution (Beyotime, China) was added to the 96-well plates and incubated in 37 °C for 2 h. The absorbance at 450 nm was detected by a Varioskan LUX microplate reader (Thermo Fisher, USA) and used for calculating cell viability.

### Flow cytometry

143B and U2OS cells were seeded into 12 well-plates at a number of 1.5 × 10^5^ and 3 × 10^5^ cells/well. After treatment with MTX for 24 h, the cells were harvested and analyzed for the apoptosis levels by using Annexin V-FITC/PI Apoptosis Detection Kit (Yeasen, China) according to the manufacturer’s instructions. Briefly, cells were incubated in 200 μl binding buffer containing 5 μl Annexin V-FITC and 10 μl PI at room temperature in the dark for 15 min. The number of apoptotic cells was measured by FACSort Flow Cytometer.

### Neutral comet assays

Neutral comet assays were performed using the Comet Assay Kit (Wanleibio, China) according to the manufacturer’s protocol. Briefly, 143B and U2OS were seeded into 12 well-plates followed by treatment with MTX for 24 h. The agarose was boiled and melted in advance and pre-cooled at 37 °C. Cells were harvested and combined with molten agarose at a ratio of 1:10. 50ul of cell mixture was plated onto the slides and placed at 4 °C for 10 min. After solidification, the slides were placed in RIPA lysis buffer for 1 h, followed by incubation with electrophoretic buffer for 1 h. The slides were subjected to electrophoresis at 25 V for 25 min and incubated in neutralizing buffer for 1 h. Subsequently, drops of DNA dye were added to the slide for staining, and the images were photographed with a fluorescence microscope. The tail moment was analyzed using ImageJ software.

### Immunofluorescence (IF)

143B and U2OS were seeded into a confocal dish at a number of 2000 cells/well. After treatment with MTX for 24 h, the cells were fixed with 4% paraformaldehyde for 10 min and permeabilized with 0.5% TritonX-100 for 10 min. After incubation with goat serum, the cells were incubated with the indicated primary antibody against γ-H2AX (CST, #2577S) (Santa Cruz, #sc-517348), 53BP1 (SAB, #49517), RAD51 (SAB, #49686), and DCLRE1C (CST, #13381) (Santa Cruz, #sc-518193) at 4 °C overnight. The next day, the cells were incubated with Goat Anti-Rabbit IgG H&L (Alexa Fluor® 488) (ab150077, Abcam) and Goat Anti-Mouse IgG H&L (Alexa Fluor® 555) (ab150114, Abcam) at 37 °C for 1 h, followed by incubation with Antifade Mounting Medium with DAPI (Beyotime, China). The images were captured with LSM780 Confocal Laser Scanning Microscopes (Zeiss, Germany). The number of foci and immunofluorescence intensity were quantified using ImageJ software.

### NHEJ and HR reporter assays

NHEJ and HR reporter assays were performed using NHEJ reporter plasmid pEJ5-GFP, HR reporter plasmid DR-GFP, and nuclease plasmid Scel. Briefly, plasmid Scel was co-transfected with pEJ5-GFP or pDR-GFP into HEK293T cells by using Lipofectamine 3000. After 24 h, the cells were treated with MTX to induce DSBs. The cells were harvested and subjected to a flow cytometer to analyze the proportion of GFP-positive cells.

### NER and BER efficiency assays

NER and BER reporter assays were performed according to previous studies [15, 16]. Briefly, for NER efficiency assays, pmaxGFP plasmid was treated with UVC irradiation, while for BER efficiency assays, pmaxGFP was treated with methylene blue at a concentration of 500 μM, and the mixture was irradiated with visible light. The damaged pmaxGFP plasmid was cotransfected with pmCherry-C1, which served as an internal control. After 3days of transfection, cells were harvested for FACS analysis. The ratio of GFP+ cells to mCherry+ cells was used as a measure of NER or BER efficiency.

### mRNA decay assay

An equal number of 143B cells were seeded into 12-well plates. After the addition of 30 μg/ml actinomycin D (Abmole, China), total RNA was extracted at the indicated times. Equal amounts of RNA were used for reverse transcription and qRT-PCR to measure the mRNA expression. GAPDH was used as the internal control.

### RNA immunoprecipitation (RIP) assay

According to the manufacturer’s instructions for the RNA Immunoprecipitation Kit (Geneseed, China), 1 × 10^7^ cells were harvested and lysed with lysis buffer. Part of the cell lysate was removed as the Input group, and the rest was incubated with protein A + G beads and ZFP36L1 antibody or IgG with rotation overnight at 4 °C. The next day, the RNA that had bound to the beads was extracted and eluted in RNase-free water, and DNA was removed. The enrichment of certain fragments was analyzed by RT-qPCR and electrophoresis. All primers used in this study are listed in Table S2.

### RNA pull-down assay

For the RNA pull-down assay, the DCLRE1C 3’UTR, HIF1A 3’UTR, and VEGFA 3’UTR were synthesized by using T7 RNA polymerase (Thermo Fisher Scientific, USA) and subsequently biotinylated by using biotinylated RNA labeling mix (Sigma, USA). RNA pull-down assays were performed using the PureBinding^TM^ RNA‒Protein Pull-Down Kit (Geneseed, China) according to the manufacturer’s instructions. Briefly, 1 × 10^7^ cells were harvested and lysed with 1x capture buffer, followed by incubation with magnetic beads and biotin-labeled probes under rotation at 4 °C for 1 h. Protein bound to the beads was eluted with loading buffer (Beyotime, China) and analyzed by western blotting.

### Luciferase activity assay

The DCLRE1C mRNA 3’UTR sequence (wild-type or mutant) and the TNF mRNA 3’UTR sequence were fused to the Renilla-PEST reporter gene. 8 × 10^4^ HEK293T cells were seeded into the 48-well plates and transfected with the reporter plasmids and oeZFP36L1 or shZFP36L1 plasmids using Lipofectamine 3000. After 48 h, luciferase activity was assessed using a Dual Luciferase Reporter Assay kit (Promega, USA).

### Statistical analysis

The data were expressed as mean ± standard deviations (SD), which were obtained from at least three independent experiments. The variability within each dataset is represented as SD in all figures. The groups exhibited similar variance. FlowJo v10.6.1 software (TreeStar) was used for flow cytometry data analysis. WB data are representative of three independent experiments unless specified in the legends. All statistical analyses were performed with GraphPad Prism 8 software. Differences between the two groups were compared using a two-tailed unpaired Student’s *t*-test. One-way or two-way ANOVA was used for multiple comparisons. Pearson’s correlation analysis was performed to evaluate the colocalization in IF assays and the correlation between ZFP36L1 expression levels and c-caspase3 or c-PARP expression levels in OS patients. The log-rank test was used for statistical analysis of overall survival in the Therapeutically Applicable Research to Generate Effective Treatments (TARGET) Osteosarcoma database. All statistical tests were justified as appropriate, and the data met the assumptions of normal distribution. *p* value < 0.05 was considered statistically significant (*p < 0.05; **p < 0.01; NS, not significant).

## Results

### Low Expression of ZFP36L1 Identified as a Hallmark of MTX Resistance

**Fig. 1:**
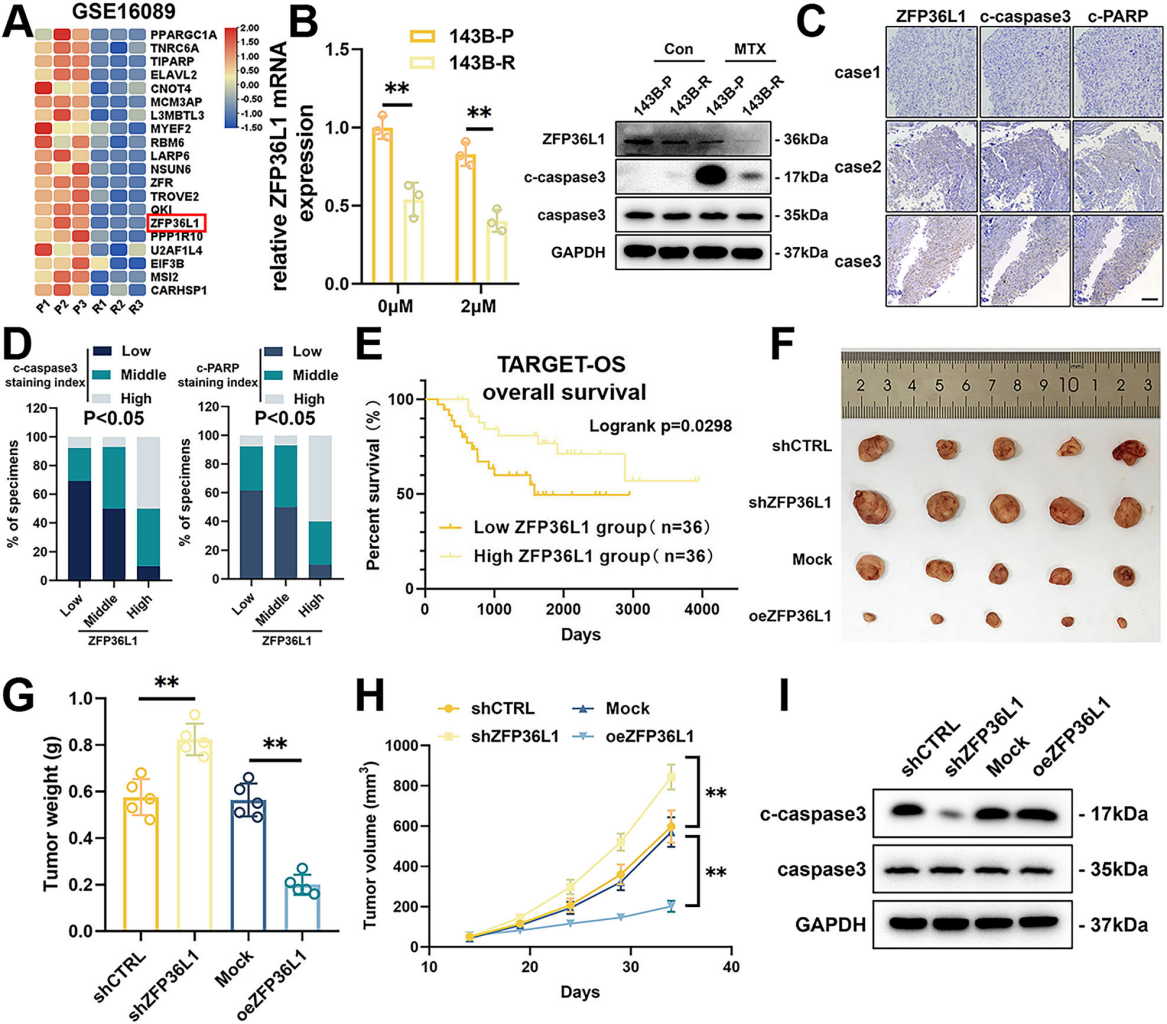
Downregulation of ZFP36L1 is correlated with the MTX resistance of OS. **A** heat maps showing the expression of RBPs with significant differences in GSE16089 (parental Saos2 cells vs. resistant Saos2 cells). **B** qPCR and WB assays were performed to detect the expression of ZFP36L1 and apoptosis marker c-caspase3 in MTX-resistant (143B-R) and parental (143B-P) 143B cells. **C** Representative IHC images of ZFP36L1, c-caspase3, and c-PARP in individual samples from among 37 OS specimens. Scale bar, 200 μm. **D** the percentage of samples with low or high ZFP36L1 expression compared to the expression of c-caspase3 and c-PARP. **E** Kaplan-Meier survival analysis was performed to investigate the association between ZFP36L1 expression levels and overall survival in OS patients. Photograph (**F**) of xenograft tumors from mice injected with 143B cells expressing different levels of ZFP36L1 after MTX treatment. Tumor weights (**G**) and volumes (**H**) were measured. **I** WB assays were performed to detect apoptosis-associated proteins in xenograft tumors grown in vivo. The data are shown as means ± SEMs; *p < 0.05, **p < 0.01.

While our previous research identified ZFP36L1 as a tumor suppressor that inhibits lung metastasis in OS [8], its role in chemoresistance remains unclear. By analyzing a published dataset (GSE16089), which compared Saos-2 OS cells sensitive to MTX with Saos-2 cells resistant to MTX, we observed significant downregulation of ZFP36L1 in MTX-resistant Saos-2 cells (Fig. 1A). Similarly, MTX-resistant 143B cells (143B-R), generated in our laboratory (Supplementary Fig. 1A–C), exhibited markedly reduced ZFP36L1 expression compared to their MTX-sensitive parental counterparts (143B-P) (Fig. 1B). These findings suggest a potential link between ZFP36L1 downregulation and MTX resistance in OS.

IHC analyses of OS patient tissues revealed a positive correlation between ZFP36L1 expression and apoptotic markers cleaved-caspase3 (c-caspase3) and cleaved-PARP (c-PARP), indicating that ZFP36L1 facilitates chemotherapy-induced apoptosis in OS cells (Fig. 1C, D). Analysis of the TARGER-OS database further revealed that high ZFP36L1 expression was associated with prolonged overall survival in OS patients (Fig. 1E). In vivo studies using ZFP36L1 knockdown (shZFP36L1) or overexpression (oeZFP36L1) 143B cell lines injected into nude mice showed that ZFP36L1 downregulation significantly enhanced tumor survival from MTX treatment (Fig. 1F–H, Supplementary Fig. 1D, E). Furthermore, WB assays confirmed that apoptosis levels were reduced in shZFP36L1 cells (Fig. 1I). However, under untreated conditions, ZFP36L1 had no discernible effect on tumor growth (Supplementary Fig. 2). Collectively, these results indicate that ZFP36L1 functions as a suppressor of chemoresistance in OS.

### Low Expression of ZFP36L1 Reduces MTX-induced DNA Damage in OS

**Fig. 2:**
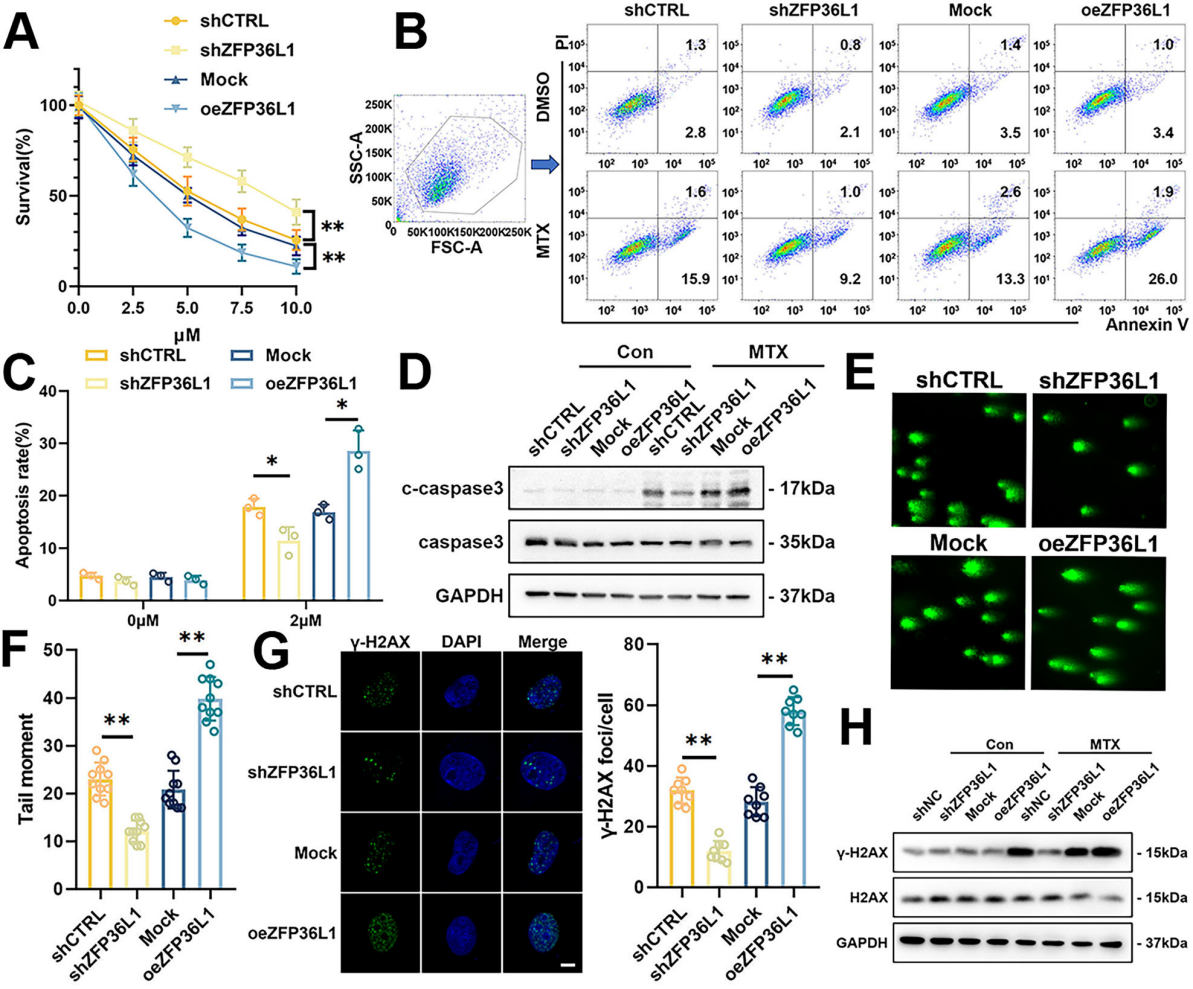
ZFP36L1 promotes apoptosis and DSBs induced by MTX treatment in 143B cells. **A** CCK-8 assays were performed to measure the survival rate of 143B cells after MTX treatment. **B**, **C** flow cytometry was performed to measure the proportion of apoptotic 143B cells after MTX treatment (**B**). The percentage of apoptotic cells was quantified (**C**). **D** WB assays were performed to detect the expression of apoptosis marker c-caspase3 in 143B cells after MTX treatment. **E**, **F** comet assays were performed to detect the level of DNA damage in 143B cells after MTX treatment (**E**), and the tail moment was quantified (**F**). **G** IF assays were performed to detect the formation of γ-H2AX foci in the nucleus of 143B cells after MTX treatment, and the number of γ-H2AX foci in each cell was quantified. Scale bar, 10 μm. **H** WB assays were performed to detect the expression of γ-H2AX in 143B cells after MTX treatment. The data are shown as means ± SEMs; *p < 0.05, **p < 0.01.

To investigate the role of ZFP36L1 in MTX resistance, we performed CCK8 assays, which showed that shZFP36L1 significantly increased the viability of 143B and U2OS cells under MTX treatment (Fig. 2A, Supplementary Fig. 3A). Flow cytometry further confirmed that shZFP36L1 reduced the percentage of apoptotic cells under MTX exposure (Fig. 2B, C, Supplementary Fig. 3B, C). Consistently, WB assays demonstrated downregulation of apoptosis-related proteins, such as c-caspase3, in shZFP36L1 cells (Fig. 2D, Supplementary Fig. 3D). These results indicate that downregulation of ZFP36L1 played a direct role in MTX resistance.

To further assess the long-term therapeutic effect of ZFP36L1 overexpression on MTX sensitivity, we constructed a doxycycline-inducible ZFP36L1 plasmid. Long-term MTX treatment assays demonstrated that induced ZFP36L1 overexpression enhanced MTX sensitivity in OS cells in vitro and in vivo (Supplementary Fig. 4A–D). Consistently, ZFP36L1 overexpression reversed MTX resistance in 143B-R cells (Supplementary Fig. 4E–H). These results indicate a sustained therapeutic effect of ZFP36L1 overexpression in long-term MTX treatment.

DNA damage is one of the primary cytotoxic mechanisms of MTX treatment [17]. To investigate whether ZFP36L1 has an impact on DNA damage under MTX treatment, we conducted Comet assays, which revealed that shZFP36L1 significantly shortened the comet tail length, indicating protection against DNA damage (Fig. 2E, F, Supplementary Fig. 5A). Given that histone H2AX is rapidly phosphorylated to γ-H2AX in response to double-strand breaks (DSBs; a severe form of DNA damage) [18], we further evaluated γ-H2AX expression through IF and WB assays. IF assays showed reduced γ-H2AX foci in shZFP36L1 cells (Fig. 2G, Supplementary Fig. 5B), which was corroborated by the WB results showing decreased γ-H2AX expression (Fig. 2H, Supplementary Fig. 5C). Building on these findings, we further explored the role of ZFP36L1 in DNA damage responses to cisplatin (Cis) and radiotherapy (IR). We found that ZFP36L1 knockdown decreased DNA damage induced by both therapeutic modalities (Supplementary Fig. 6). Taken together, these findings suggest that low expression of ZFP36L1 reduced DNA damage induced by genotoxic therapy, thereby promoting therapy resistance.

### ZFP36L1 inhibits DSBs repair via the NHEJ pathway

**Fig. 3:**
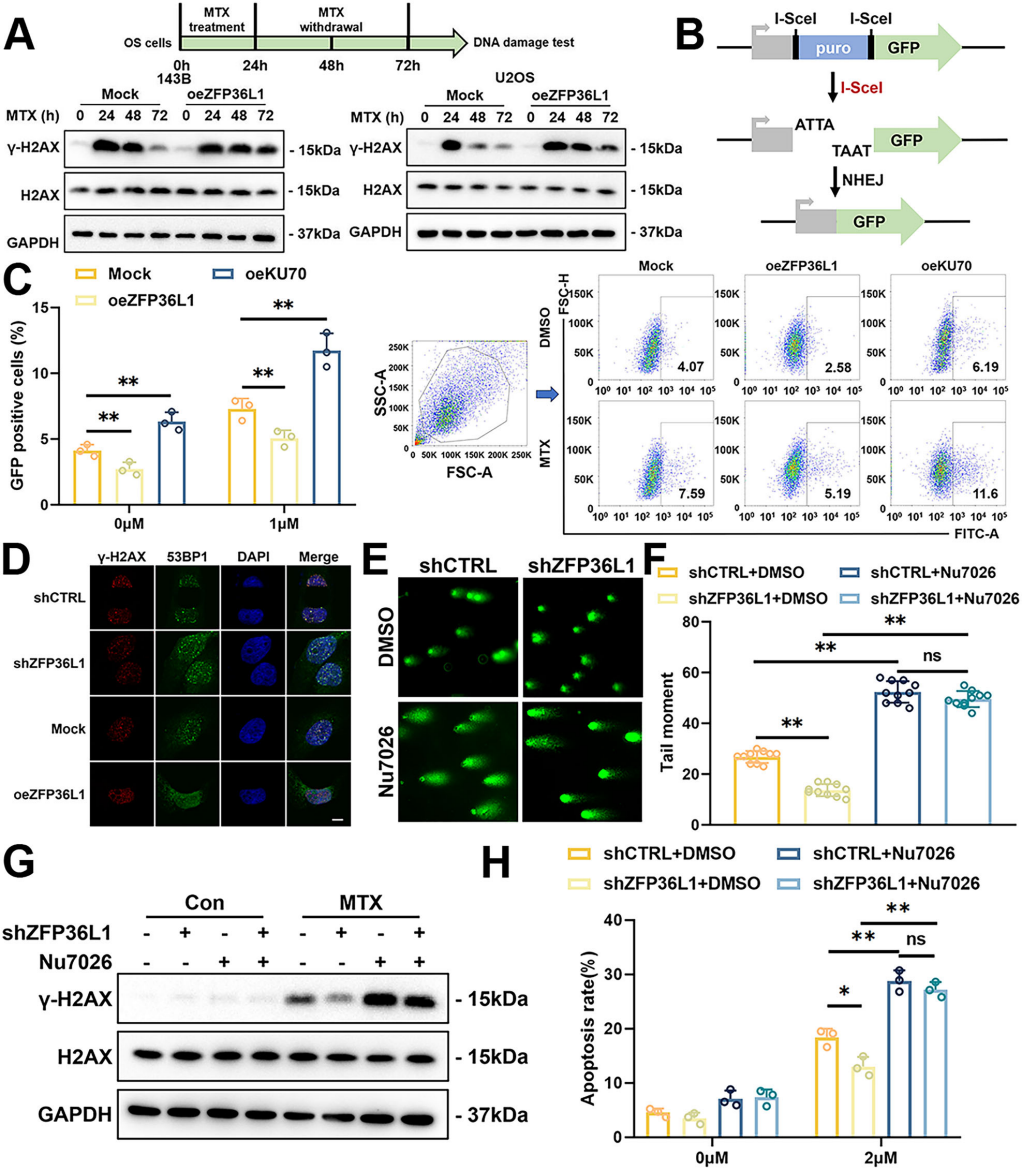
ZFP36L1 inhibited DSBs repair through blocking the NHEJ pathway in OS. **A** OS cells were treated with MTX for 24 hours, followed by drug withdrawal. WB assays were performed to detect the expression of γ-H2AX in OS cells at the indicated time points. **B** schematic representation of the EJ5-GFP plasmid to measure the capability of NHEJ repair. **C** flow cytometry was performed to measure the proportion of GFP-positive cells in NHEJ reporter assays. **D** IF assays were performed to detect the formation of 53BP1 foci in the nucleus of OS cells after MTX treatment. Scale bar, 10 μm. **E, F** Comet assays were performed to detect the level of DNA damage in 143B cells after MTX treatment with or without Nu7026 treatment (**E**), and the tail moment was quantified (**F**). **G** WB assays were performed to detect the expression of γ-H2AX in 143B cells after MTX treatment with or without Nu7026 treatment. **H** flow cytometry was performed to measure the proportion of apoptotic 143B cells after MTX treatment with or without Nu7026 treatment. The data are shown as means ± SEMs; *p < 0.05, **p < 0.01.

To investigate how ZFP36L1 regulated DNA damage, we monitored γ-H2AX expression over time. After 24 hours of MTX treatment, both the mock and oeZFP36L1 groups rapidly generated a large amount of γ-H2AX. However, following the removal of MTX, the oeZFP36L1 group exhibited a slower rate of γ-H2AX reduction, suggesting that the DNA damage repair mechanism is impaired in the oeZFP36L1 group (Fig. 3A).

NHEJ and HR are two important pathways for DSBs repair. To assess their activity, we utilized EJ5-GFP and DR-GFP plasmid reporters, which measure NHEJ and HR repair efficiency, respectively [19, 20]. In these reporters, GFP expression is restored when DSBs are repaired via NHEJ (EJ5-GFP) or HR (DR-GFP) following the introduction of DSBs by the I-SceI nuclease (Fig. 3B, Supplementary Fig. 7A). Upon transfecting Mock and oeZFP36L1 HEK293T cells with these plasmids, we observed that oeZFP36L1 significantly reduced the percentage of GFP-positive cells in the EJ5-GFP reporter group, indicating impaired NHEJ activity (Fig. 3C). In contrast, no significant effect was observed in the DR-GFP reporter group (Supplementary Fig. 7B, C). Furthermore, we found that prolonged exposure to MTX in OS cells led to upregulated ZFP36L1 expression, which was accompanied by enhanced NHEJ repair capacity (Supplementary Fig. 7D–F).

To further investigate the repair pathway involved, we examined the recruitment of DNA damage repair proteins, such as 53BP1 (a key regulator in the NHEJ pathway) [21] and RAD51 (a key enzyme in the HR pathway) [22], in 143B cells treated with MTX. The results showed that shZFP36L1 promoted the formation of 53BP1 foci at DSB sites (Fig. 3D, Supplementary Fig. 8A), with minimal impact on RAD51 foci (Supplementary Fig. 8B). These results suggest that ZFP36L1 primarily inhibits the NHEJ pathway rather than affecting the HR pathway.

To explore whether ZFP36L1 downregulation promotes MTX resistance through the NHEJ pathway, we performed rescue experiments using Nu-7026, a DNA-PK inhibitor. First, we demonstrated that Nu-7026 effectively inhibited the NHEJ pathway in OS cells (Supplementary Fig. 8C). Functional assays further revealed that treatment with Nu-7026 reduced DSB repair capacity and attenuated MTX resistance in shZFP36L1 OS cells (Fig. 3E–H, Supplementary Fig. 9). Conversely, the HR pathway inhibitor RI-2 had minimal effect on MTX resistance in shZFP36L1 cells (Supplementary Fig. 10).

While nucleotide excision repair (NER) and base excision repair (BER) repair pathways play crucial roles in the repair of single-strand breaks (SSBs) induced by chemotherapeutic agents [23], we further investigated the role of ZFP36L1 in the NER and BER repair pathways. The result showed that ZFP36L1 had a negligible impact on the NER and BER repair pathways (Supplementary Fig. 11) [15, 16]. Collectively, these findings indicate that ZFP36L1 primarily impairs the NHEJ pathway, thereby exacerbating MTX-induced DNA damage and cell death.

Given that PARP1 inhibitors, which target the SSB repair pathway, have been clinically approved for cancers with DNA repair defects [24], we further investigated the synergistic effect of ZFP36L1 overexpression with PARP1 inhibitors. In vitro and in vivo experiments showed that this combination enhanced MTX-induced DNA damage and tumor suppression (Supplementary Fig. 12). These findings suggest that PARP1 inhibitors combined with ZFP36L1 overexpression may potentiate MTX efficacy in OS.

### ZFP36L1-mediated Inhibition of NHEJ Pathway Depends on DCLRE1C

**Fig. 4:**
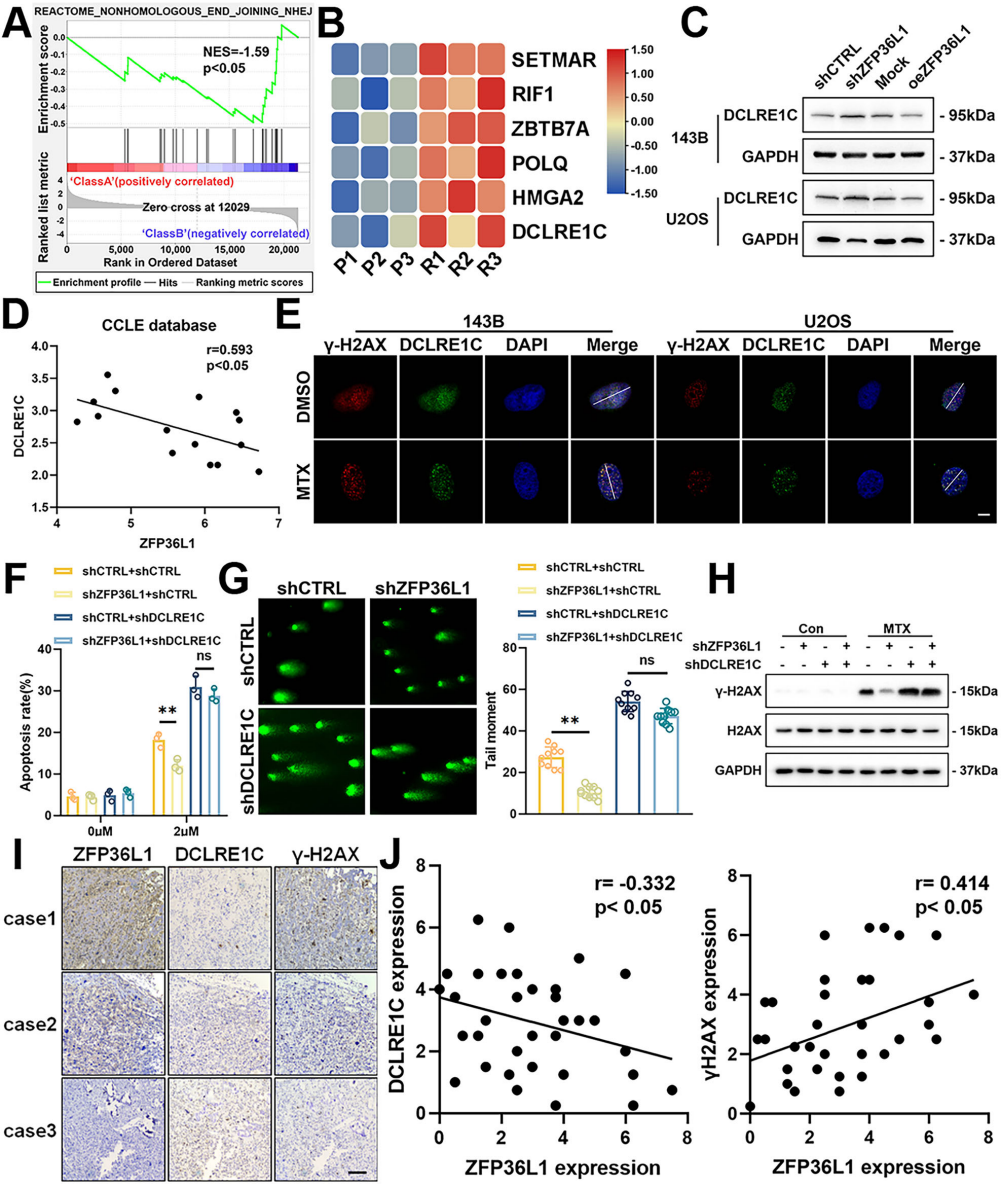
ZFP36L1 inhibits NHEJ by reducing DCLRE1C expression. **A** the gene expression profiles of parental and MTX-resistant Saos2 cells subjected to Gene Set Enrichment Analysis (GSEA) using an NHEJ pathway-associated gene set. **B** a heat map showing the expression of NHEJ-associated genes with significant differences in GSE16089. **C** WB assays were performed to detect the expression of DCLRE1C in OS cells. **D** linear correlations between the expression of ZFP36L1 and DCLRE1C in OS cell lines according to the CCLE database. **E** IF assays were performed to detect the colocalization of DCLRE1C and γ-H2AX in 143B and U2OS cells after MTX treatment. Scale bar, 10 μm. **F** flow cytometry was performed to measure the proportion of apoptotic 143B cells after MTX treatment. **G** comet assays were performed to detect the level of DNA damage in 143B cells after MTX treatment. **H** WB assays were performed to detect the expression of γ-H2AX in 143B cells after MTX treatment. **I** Representative IHC images of ZFP36L1, DCLRE1C, and γ-H2AX in individual samples from among 37 human OS specimens. Scale bar, 200 μm. **J** Linear correlations between ZFP36L1 and DCLRE1C, ZFP36L1 and γ-H2AX, according to IHC staining of human OS specimens. The data are shown as means ± SEMs; *p < 0.05, **p < 0.01.

In coordination with the low expression of ZFP36L1, we found NHEJ pathway was activated in MTX-resistant Saos-2 cells (GSE16089) (Fig. 4A). Among these genes, HMGA2, ZBTB7A, and DCLRE1C were significantly upregulated in MTX-resistant Saos-2 cells (Fig. 4B). Further expression analysis in 143B and U2OS cells revealed an increase in DCLRE1C expression in shZFP36L1 cells and a decrease in oeZFP36L1 cells (Fig. 4C, Supplementary Fig. 13A). Additionally, data from the CCLE database demonstrated a negative correlation between ZFP36L1 and DCLRE1C expression in OS cell lines (Fig. 4D). These findings suggest that ZFP36L1 might regulate DCLRE1C expression.

DCLRE1C plays a crucial role in the NHEJ pathway as a nuclease that processes DNA ends upon activation by DNA-PKcs in response to DSBs. However, its function in the OS remains unclear. In our study, we observed that both DCLRE1C and 53BP1 were recruited to DSB sites in OS cells with MTX treatment (Fig. 4E, Supplementary Fig. 13B–D). Furthermore, overexpression of DCLRE1C (oeDCLRE1C) significantly enhanced DNA damage repair and reduced apoptosis levels in these cells under MTX treatment (Supplementary Fig. 14).

Rescue assays further confirmed the role of DCLRE1C in ZFP36L1-mediated effects. Knockdown of DCLRE1C (shDCLRE1C) reversed the protective effect of shZFP36L1 against DNA damage and MTX-induced apoptosis in OS cells (Fig. 4F–H, Supplementary Fig. 15). Furthermore, IHC assays conducted on OS patient samples revealed an inverse relationship between ZFP36L1 and DCLRE1C expression, as well as between DCLRE1C and γ-H2AX levels (Fig. 4I, J). Collectively, these findings suggest that ZFP36L1 inhibits the NHEJ pathway by regulating DCLRE1C expression.

### ZFP36L1 regulates DCLRE1C expression by promoting mRNA degradation

**Fig. 5:**
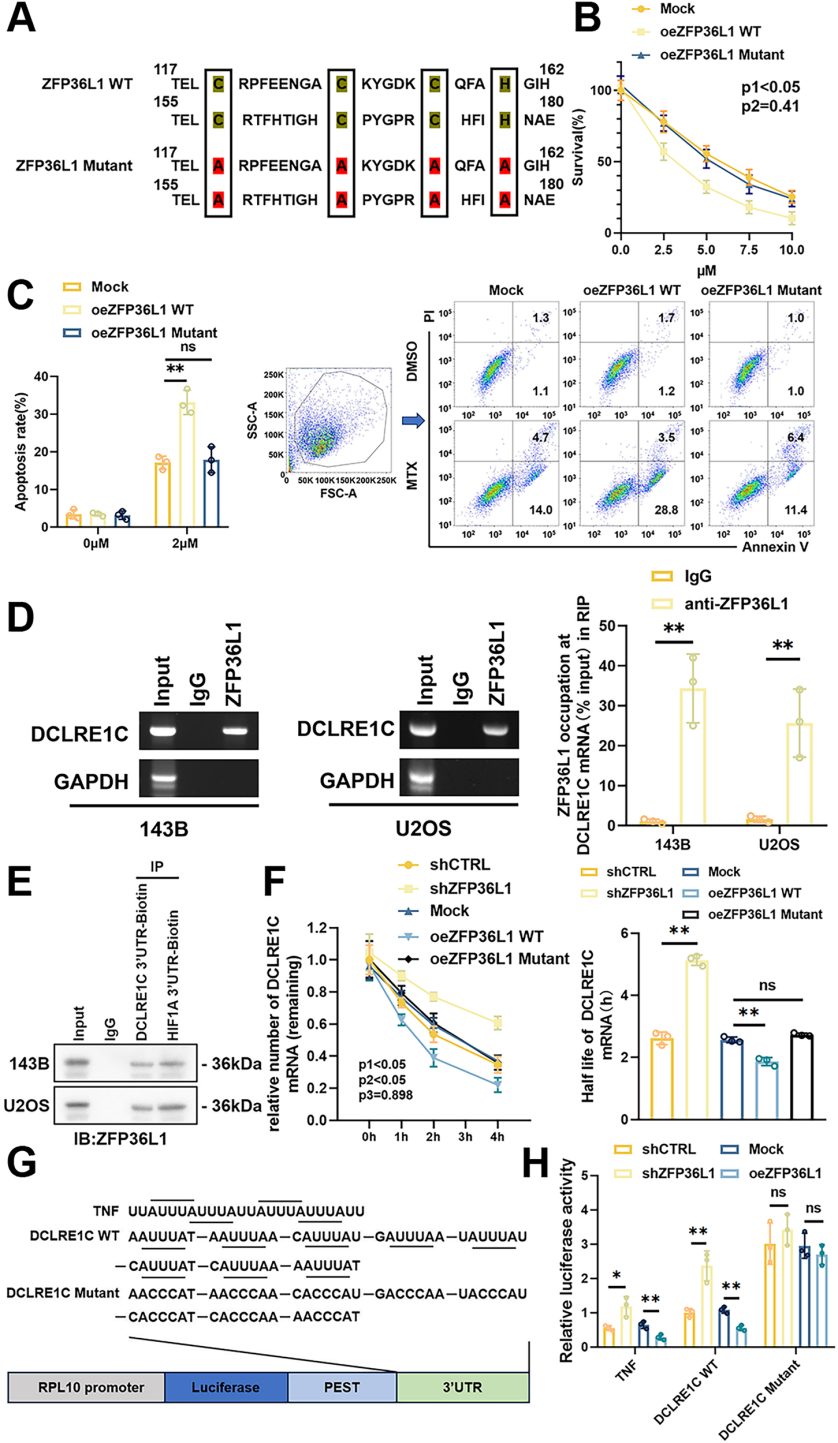
ZFP36L1 promotes DCLRE1C mRNA degradation by binding to the ARE element. **A** Schematic representation of the CCCH-type zinc-fingers in ZFP36L1 and construction of the corresponding mutant protein. **B** CCK-8 assays were performed to determine the cell viability of 143B cells after MTX treatment. p1, Mock vs. oeZFP36L1 WT; p2, Mock vs. oeZFP36L1 Mutant. **C** flow cytometry was performed to measure the proportion of apoptotic 143B cells after MTX treatment. **D** RIP assays were performed to detect the interaction between ZFP36L1 and DCLRE1C mRNA in OS cells. **E** RNA pull-down assays were performed to detect the function of 3’UTR of DCLRE1C mRNA in the binding relation between ZFP36L1 and DCLRE1C mRNA. HIF1A 3’UTR was used as a positive control. **F** mRNA stability assays were performed to determine the stability of DCLRE1C mRNA regulated by ZFP36L1, and the half-life of DCLRE1C mRNA was analyzed. **G, H** Schematic representation (**G**) of the predicted AREs in the 3’UTR of DCLRE1C mRNA (NM_001033855.3) and construction of corresponding reporter plasmids. The 3’UTR in TNF mRNA (NM_000594.4) was used as a positive control. Luciferase activity (**H**) was detected by dual-luciferase reporter assays. The data are shown as means ± SEMs; *p < 0.05, **p < 0.01.

ZFP36L1 has been shown to participate in the degradation of several mRNAs via its zinc finger structure, which binds to mRNA directly [7]. To investigate whether ZFP36L1 regulates MTX resistance relying on its RNA binding property, we constructed a mutant ZFP36L1 protein in which the eight conserved amino acids in the zinc finger domain (CCCH-CCCH) were replaced with alanine (A) (Fig. 5A). Unlike the wild-type ZFP36L1 (ZFP36L1 WT), the mutant ZFP36L1 (ZFP36L1 Mutant) showed minimal ability to reduce MTX resistance in OS cells, indicating ZFP36L1 regulates MTX resistance by relying on its RNA binding property (Fig. 5B, C).

To further investigate the interaction between ZFP36L1 and DCLRE1C mRNA, RNA immunoprecipitation (RIP) assays were conducted and the results confirmed that ZFP36L1 directly binds to DCLRE1C mRNA (Fig. 5D). Previous research has shown that ZFP36L1 binds to the 3′ untranslated region (3′UTR) of target mRNAs [7]. Therefore, we constructed biotin-labeled DCLRE1C mRNA 3′UTR to perform RNA pull-down assays. The results showed that DCLRE1C mRNA 3’UTR effectively pulled down ZFP36L1, similar to HIF1A mRNA 3′UTR, a known target of ZFP36L1 (Fig. 5E). However, the ZFP36L1 Mutant protein showed negligible interaction with DCLRE1C mRNA (Supplementary Fig. 16A, B). These findings establish that ZFP36L1 directly binds to the 3′UTR of DCLRE1C mRNA in a zinc finger-dependent manner.

Given that ZFP36L1 binds to and promotes target mRNA degradation, we next examined its role in DCLRE1C mRNA decay. Using actinomycin D, we observed a significant decrease in the half-life of DCLRE1C mRNA in oeZFP36L1 WT OS cells, while no changes were detected in oeZFP36L1 Mutant OS cells (Fig. 5F). This result further underscores that ZFP36L1 promotes DCLRE1C mRNA decay, primarily through its RNA-binding capability.

Previous studies have shown that ZFP36L1 binds to ARE sequences (AUUUA motifs) in 3′UTRs of mRNAs [7]. By scanning the 3′UTR of DCLRE1C mRNA, we identified 8 candidate AREs. To assess the role of these AREs in ZFP36L1-mediated degradation of DCLRE1C mRNA, we generated Renilla luciferase reporter plasmids containing wild-type or mutant ARE sequences. Dual-luciferase assays revealed that ZFP36L1 overexpression significantly reduced luciferase activity in the wild-type DCLRE1C group, but had minimal impact on the mutant group (Fig. 5G, H). These findings demonstrate that ZFP36L1 regulates DCLRE1C mRNA degradation through its interaction with AREs in the 3′UTR.

To elucidate the role of ZFP36L1-DCLRE1C mRNA interaction in MTX resistance, we generated two distinct DCLRE1C expression constructs: one containing its wild-type 3′UTR (DCLRE1C WT), and the other with its 3′UTR in which the AREs were mutated into ACCCA motifs (DCLRE1C Mutant). Functional assays demonstrated that ZFP36L1 overexpression specifically attenuates DCLRE1C WT-mediated MTX resistance, with no observable impact on DCLRE1C Mutant (Supplementary Fig. 16C–E). These results confirm that ZFP36L1 primarily relies on its interaction with DCLRE1C mRNA to enhance MTX sensitivity in OS cells.

Given the conserved role of ZFP36 family proteins in ARE-dependent mRNA regulation [25], we further investigated their potential roles in MTX resistance in OS. Results showed that both ZFP36 and ZFP36L2 could enhance MTX sensitivity in OS cells (Supplementary Fig. 17A–C). However, further analysis revealed a compensatory regulation of ZFP36L2 in ZFP36L1-modulated OS cells, with no such effect on ZFP36. Based on these findings, we co-overexpressed ZFP36L1 and ZFP36L2, which modestly enhanced OS cell sensitivity to MTX (Supplementary Fig. 17D–F). These results indicate potential compensatory roles of ZFP36L1 and ZFP36L2 in the development of MTX resistance in OS.

### The ZFP36L1-DCLRE1C-NHEJ axis regulates MTX sensitivity in OS

**Fig. 6:**
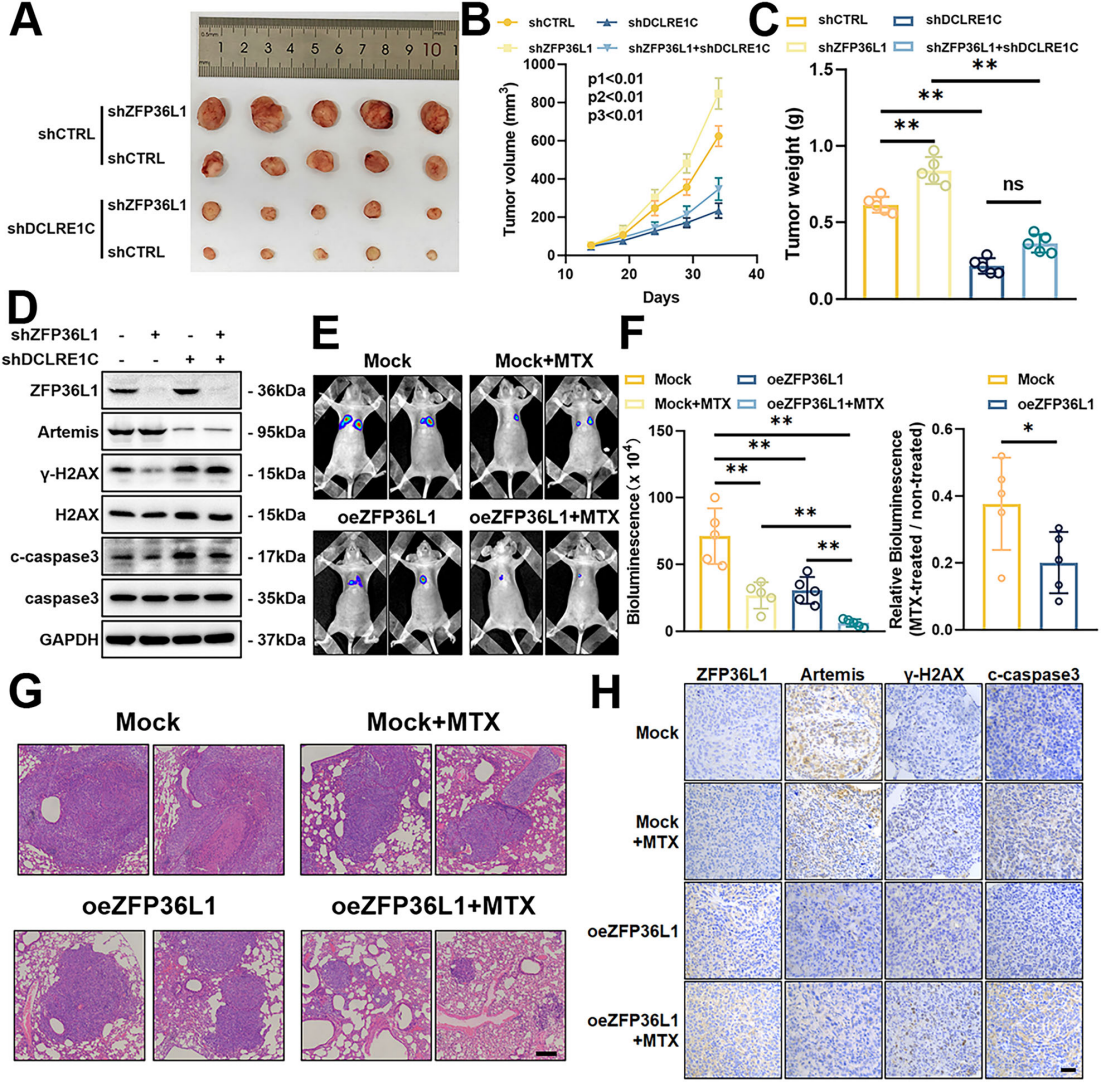
Targeting the ZFP36L1-DCLRE1C axis reverses MTX resistance in OS. **A–D** Photograph (**A**) of xenograft tumors from mice injected with shZFP36L1 or shDCLRE1C 143B cells after MTX treatment. Tumor weights (**B**) and volumes (**C**) were measured. The expression of ZFP36L1, DCLRE1C, γ-H2AX, and c-caspase3 in tumors was detected by WB assays (**D**). p1, shCTRL vs. shZFP36L1, p2, shCTRL vs. shDCLRE1C, p3, shZFP36L1 vs. shZFP36L1 + shDCLRE1C. **E–G** Lung metastasis of OS was measured by in vivo bioluminescence (**E**, **F**) and HE staining (**G**). Scale bar, 200 μm. **H** Representative OS metastatic lesions in the lungs from different groups of mice were analyzed by IHC staining for ZFP36L1, DCLRE1C, γ-H2AX, and c-caspase3. Scale bar, 50 μm. The data are shown as means ± SEMs; *p < 0.05, **p < 0.01.

To confirm the above findings in vivo, we established a xenograft tumor model by subcutaneously injecting 143B cells with shZFP36L1 or shDCLRE1C into nude mice. After allowing tumors to establish for two weeks, followed by three weeks of MTX treatment, the mice were euthanized, and tumor tissues were analyzed. Tumor weight measurements and WB assays demonstrated that shZFP36L1 cells exhibited resistance to MTX. However, this resistance was significantly reduced by shDCLRE1C, indicating its critical role in mediating MTX resistance (Fig. 6A–C). WB assays further revealed that shDCLRE1C enhanced MTX therapeutic efficacy by promoting DNA damage in OS cells (Fig. 6D).

Given the importance of chemotherapy in OS patients with lung metastases, where MTX resistance is a frequent challenge, we further investigated the impact of ZFP36L1 on MTX resistance in metastatic OS cells. We established a high-metastasis model by injecting 143B cells into the tail vein of nude mice. Consistent with our previous study, the results showed that oeZFP36L1 significantly inhibited lung metastases. Furthermore, we observed that the MTX-treated oeZFP36L1 group exhibited an approximately 80% decrease compared to the untreated oeZFP36L1 group, while the MTX-treated Mock group demonstrated a roughly 60% decrease relative to its untreated counterpart (Fig. 6E–G), which indicates that ZFP36L1 enhances MTX sensitivity in lung metastatic OS cells. IHC analysis further supported these findings, showing that in the MTX treatment group, oeZFP36L1 exhibited increased expression of γ-H2AX and c-caspase-3, along with reduced expression of DCLRE1C (Fig. 6H, Supplementary Fig. 18). These observations underscore the role of ZFP36L1 in overcoming MTX resistance in metastatic OS.

**Fig. 7:**
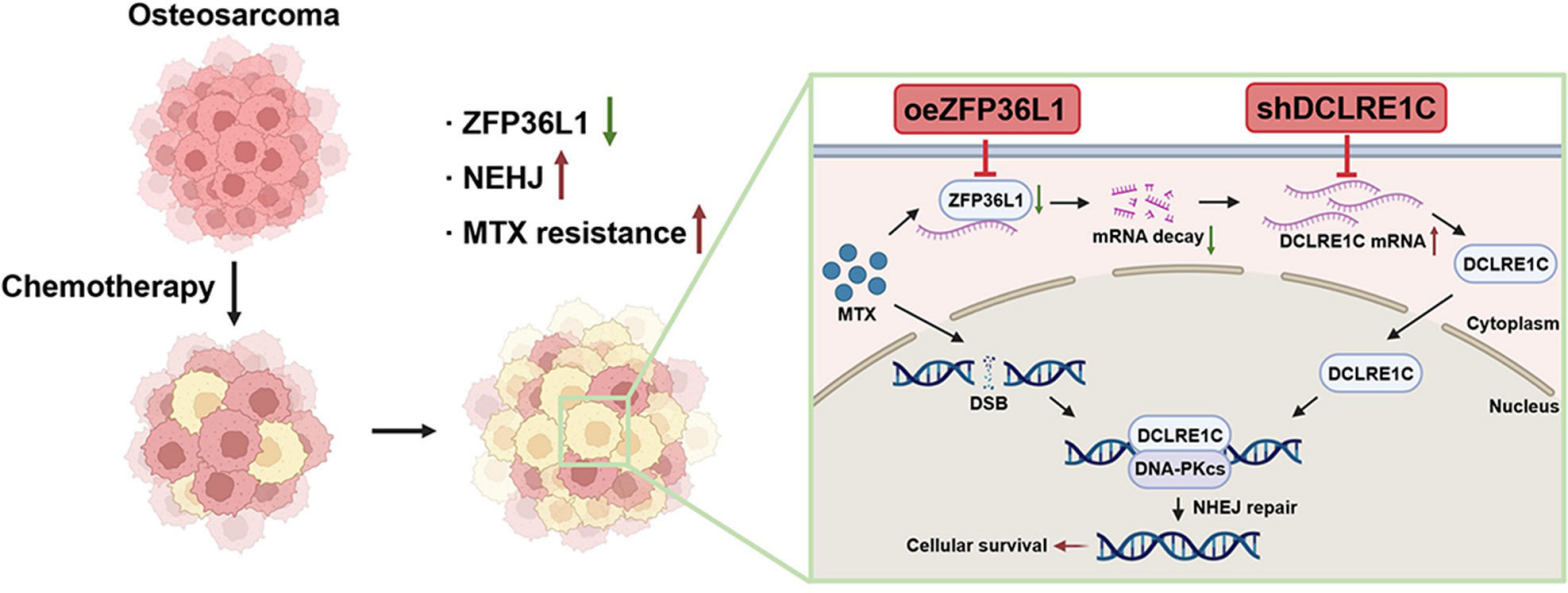
Blocking the ZFP36L1-DCLRE1C-NHEJ axis reverses MTX resistance in OS. In MTX-resistant OS cells, ZFP36L1 is abnormally downregulated, leading to decreased degradation of its downstream target, DCLRE1C mRNA. This results in upregulation of DCLRE1C protein, a key nuclease in the NHEJ repair pathway. DCLRE1C translocates to the nucleus and forms a heterodimer with DNA-PKcs to repair MTX-induced DNA damage, thereby restoring genomic stability and promoting cellular survival, ultimately contributing to MTX resistance. Overexpression of ZFP36L1 enhances its capability to degrade DCLRE1C mRNA, reducing DCLRE1C expression in MTX-resistant OS cells. This reduction compromises the NHEJ repair capacity, effectively reversing MTX resistance and inducing cellular death.

In conclusion, these findings underscore the pivotal role of the ZFP36L1-DCLRE1C-NHEJ axis in regulating MTX sensitivity in OS. Targeting this pathway, either by overexpressing ZFP36L1 or silencing DCLRE1C, effectively enhances MTX sensitivity, providing a promising therapeutic strategy for OS patients (Fig. 7).

## Discussion

The treatment of OS has stagnated over the past few decades, primarily relying on surgical intervention combined with chemotherapy agents such as MTX, Cis, and doxorubicin [26]. This approach is especially critical for patients with recurrent or metastatic OS, underscoring the urgent need to address chemotherapy resistance to improve patient outcomes. In this study, we found that ZFP36L1 expression is downregulated in MTX-resistant OS cells. This downregulation enhances their NHEJ repair capacity, leading to increased resistance to MTX-induced DNA damage and MTX resistance development. These findings underscore the pivotal role of ZFP36L1 in MTX resistance by regulating DNA repair pathways, providing a potential foundation for developing more effective therapeutic strategies to enhance OS chemotherapy outcomes.

Previous studies have demonstrated the tumor-suppressive role of ZFP36L1 in various cancers, primarily functioning through the binding of target mRNA 3’UTR sequences to accelerate their degradation. ZFP36L1 targets mRNAs involved in tumor progression, such as FGF21, ZEB2, and Cyclin D [7, 27, 28]. Consistent with these reports, our study identifies DCLRE1C mRNA as a target of ZFP36L1. ZFP36L1 promotes DCLRE1C mRNA degradation, thereby reducing DCLRE1C protein translation, a key nuclease involved in the NHEJ repair pathway [29]. Our research shows that ZFP36L1 decreases DCLRE1C protein levels, impairing NHEJ-mediated DNA repair in OS cells and enhancing their sensitivity to MTX, thus improving the chemotherapeutic effect. Furthermore, our analysis identified additional NHEJ-related genes (such as RIF1 and POLQ) whose mRNAs contain ZFP36L1 binding sites. While further investigation is needed to determine whether ZFP36L1 directly regulates the stability of these transcripts, our findings suggest that ZFP36L1 may play a broader role in modulating NHEJ-related pathways. This expanded understanding of ZFP36L1’s regulatory potential provides new insights into its contribution to overcoming chemotherapy resistance.

The ZFP36 protein family (ZFP36, ZFP36L1, ZFP36L2) mediates post-transcriptional mRNA regulation by binding AREs in target transcripts [25], with conserved function. While functional redundancies exist, emerging evidence highlights context-dependent divergences in their regulatory roles. Previous studies have shown growth-suppressive functions for ZFP36L1 [30] and ZFP36L2 [31], paradoxically, their double knockout promoted cellular proliferation [32], highlighting context-dependent functions. Our analysis of the MTX-resistant OS dataset (GSE16089) initially focused on the role of ZFP36L1 in suppressing MTX resistance. Subsequent functional assays revealed that both ZFP36 and ZFP36L2 inhibited MTX resistance in OS cells, suggesting a functional redundancy within the ZFP36 family. However, the molecular mechanisms underlying the function of ZFP36 and ZFP36L2 in MTX resistance remain undefined, which warrants dedicated investigation in future studies.

RNA-binding proteins (RBPs) have emerged as key tumor regulators, with their aberrant expression linked to chemotherapy resistance and tumor survival [33]. For example, aberrant expression of HuR links to tumor hypoxia [34], chemotherapy resistance [35], and radioresistance [36], while LARP1 serves as an oncogenic protein to promote tumor survival and progression [37, 38]. As the role of dysregulated RBPs in cancer becomes clearer, targeting these RBPs presents a promising therapeutic strategy [39]. Various methods have been developed to target RNA-binding proteins, including small molecule inhibitors [40], peptides [41], antisense oligonucleotides [42], and siRNA [43], some advancing to preclinical trials. In this study, ZFP36L1 overexpression demonstrated significant therapeutic potential in a mouse model of OS. We believe that future research focused on developing drugs to enhance ZFP36L1 stability will benefit OS treatment.

DNA damage is a critical mechanism underlying the cytotoxic effects of chemotherapy and radiotherapy. However, as resistance develops, tumor cells counteract this effect by enhancing their DNA repair capacity. Therefore, targeting DNA repair pathways has become a vital approach to overcoming chemoresistance. Clinically, DNA damage repair (DDR) inhibitors, including ATR inhibitors [44], ATM inhibitors [45], and DNA-PK inhibitors [46], have been applied to treat cancers like advanced solid cancers [47]. However, the application of DNA repair inhibitors in OS remains limited. Previous studies have established that the therapeutic efficacy of MTX [17], Cis [48], and doxorubicin [49] induces DNA damage to exert therapeutic efficacy in OS. Combinatorial strategies integrating DDR inhibitors have been shown to enhance the cytotoxicity of chemotherapeutic agents [50]. For example, PARP inhibitors like olaparib have demonstrated synergistic activity with doxorubicin in OS cells [1, 51]. Here, our study revealed that ZFP36L1 suppresses DNA damage repair, thereby amplifying MTX-induced DNA damage and sensitizing OS cells to MTX. Additionally, combining ZFP36L1 overexpression with olaparib disrupts both DSB and SSB repair pathways, further enhancing therapeutic efficacy. These findings identify ZFP36L1 as a potential target to overcome chemotherapy resistance by impairing DNA damage repair in OS.

DCLRE1C is a nuclease that resolves DNA hairpin structures and primarily forms a complex with DNA-PKcs. During NHEJ-mediated DSBs repair, DNA-PKcs auto-phosphorylates after binding to the Ku70/80 complex at the DSBs ends, activating DCLRE1C to perform its nuclease function [52]. Given the critical role of DCLRE1C in the NHEJ pathway, recent studies have focused on elucidating the structure of the DCLRE1C-DNA-PKcs complex [53] to identify potential therapeutic targets for inhibiting DCLRE1C activity. However, strategies to inhibit DCLRE1C function remain elusive. In this study, we demonstrated that ZFP36L1 expression is inversely correlated with DCLRE1C expression in OS cells. By upregulating ZFP36L1, we effectively reduced DCLRE1C function, thereby impairing NHEJ repair efficiency and enhancing MTX sensitivity in OS cells. These findings highlight a novel approach for modulating DCLRE1C activity and overcoming chemotherapy resistance in OS.

In summary, our findings reveal that ZFP36L1 accelerates the degradation of DCLRE1C mRNA, reducing DCLRE1C protein levels and impairing DNA damage repair. This leads to reduced NHEJ repair capacity in OS cells, enhancing the DNA-damaging effects of MTX. Targeting the ZFP36L1-DCLRE1C-NHEJ axis presents a promising approach for improving treatment outcomes in OS patients.

## Supplementary information

supplementary materials figures

supplementary materials tables

Original data

## Data Availability

The data generated in this study are available upon request from the corresponding author. Expression profile data analyzed in this study were obtained from Cancer Cell Line Encyclopedia (CCLE) (https://sites.broadinstitute.org/ccle/datasets), TARGET-OS database (https://ocg.cancer.gov/programs/target) and Gene Expression Omnibus (GEO) (https://www.ncbi.nlm.nih.gov/geo/) under accession numbers GSE16089 and GSE246405.
