# Supplementary material for: Downregulation of ZFP36L1 contributes to methotrexate resistance in osteosarcoma through enhanced NHEJ DNA repair mechanisms
Source: Cell Death Dis. 2025 Nov 24;16(1):852. doi: 10.1038/s41419-025-08217-4 (PMC12644584; doi:10.1038/s41419-025-08217-4)
Supplement: Supplementary file 1 — supplementary materials figures [file 41419_2025_8217_MOESM1_ESM.docx]

**Supplementary Figure 1**

**
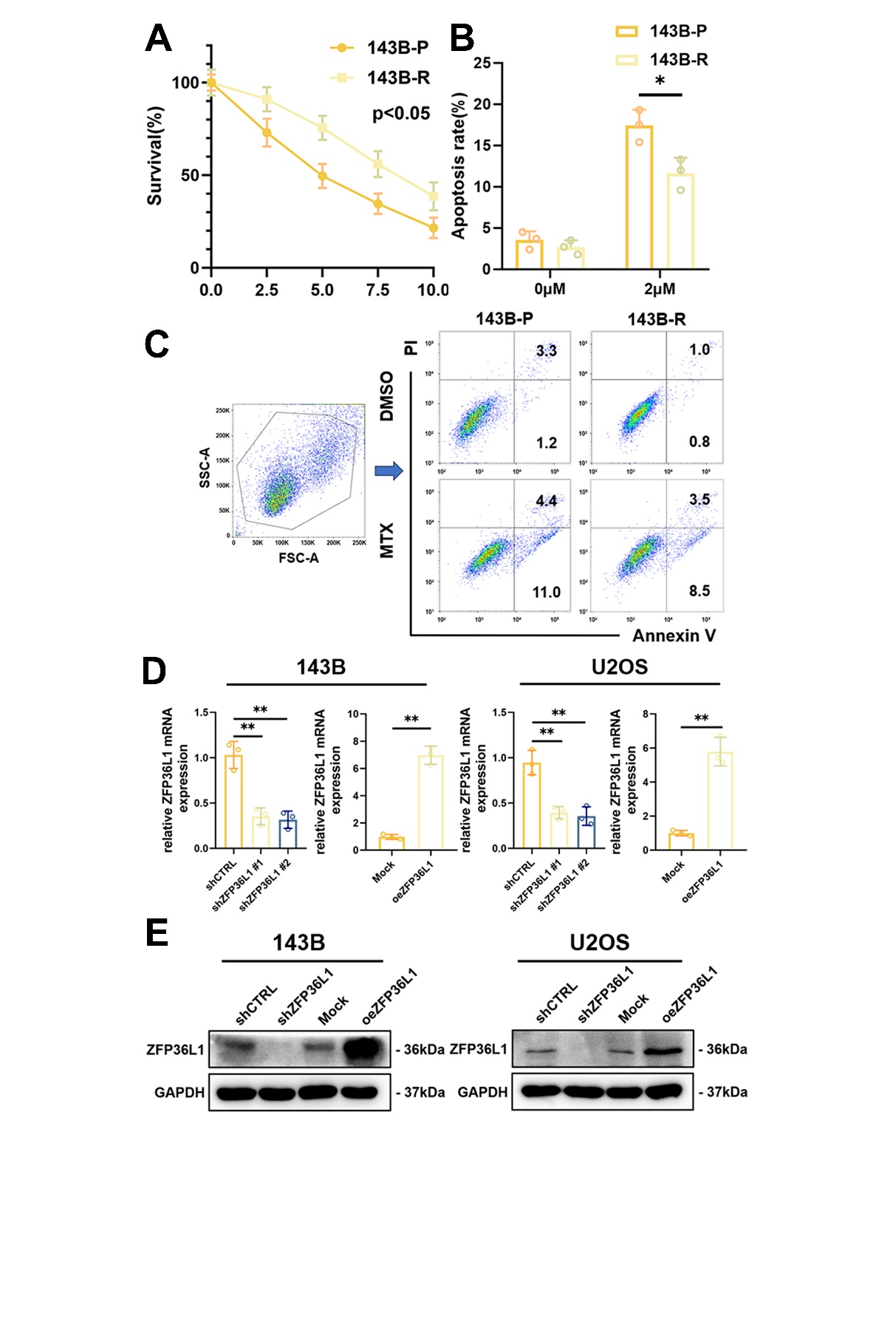
**

**Supplementary Figure 2**

**
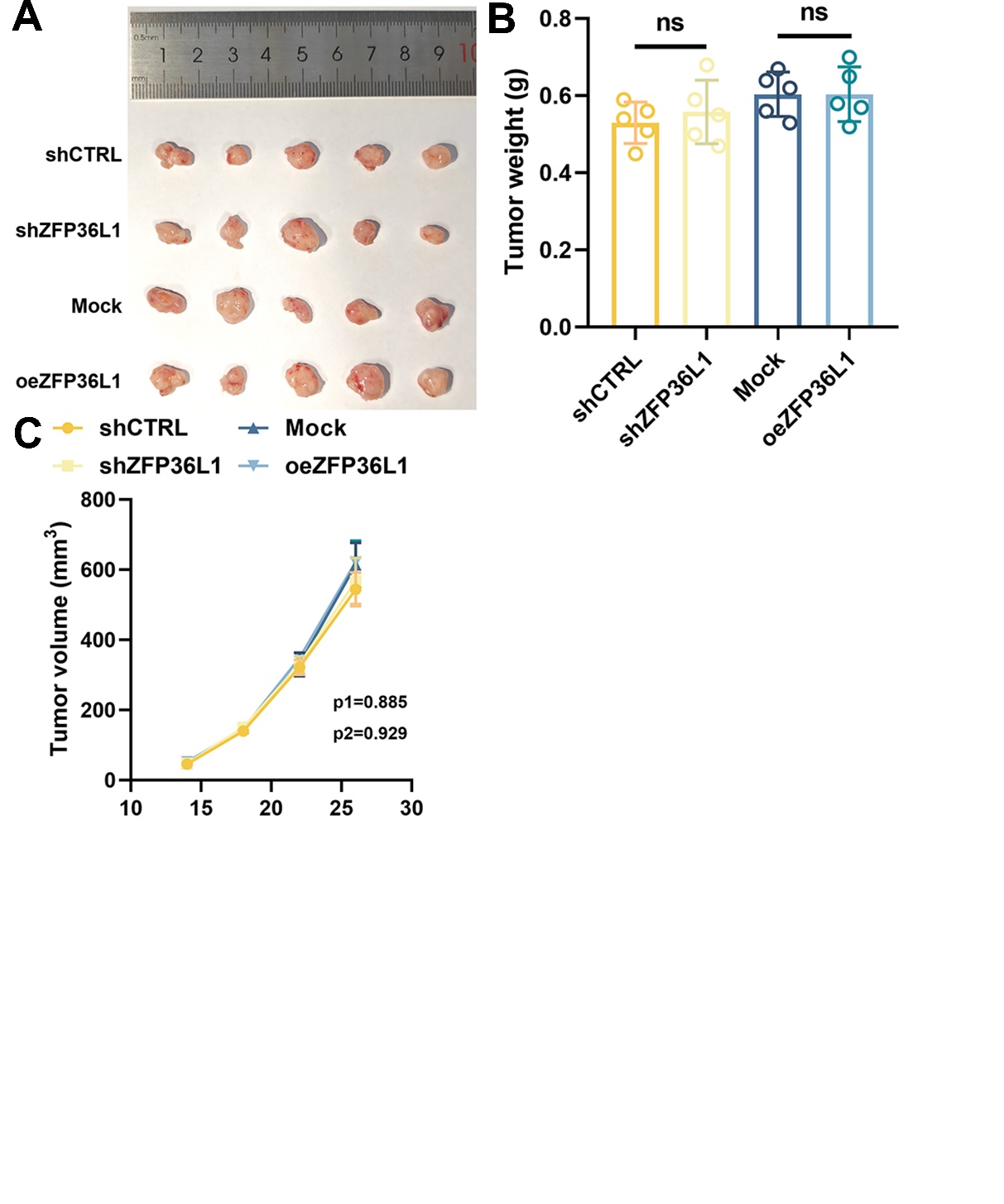
**

**Supplementary Figure 3**

**
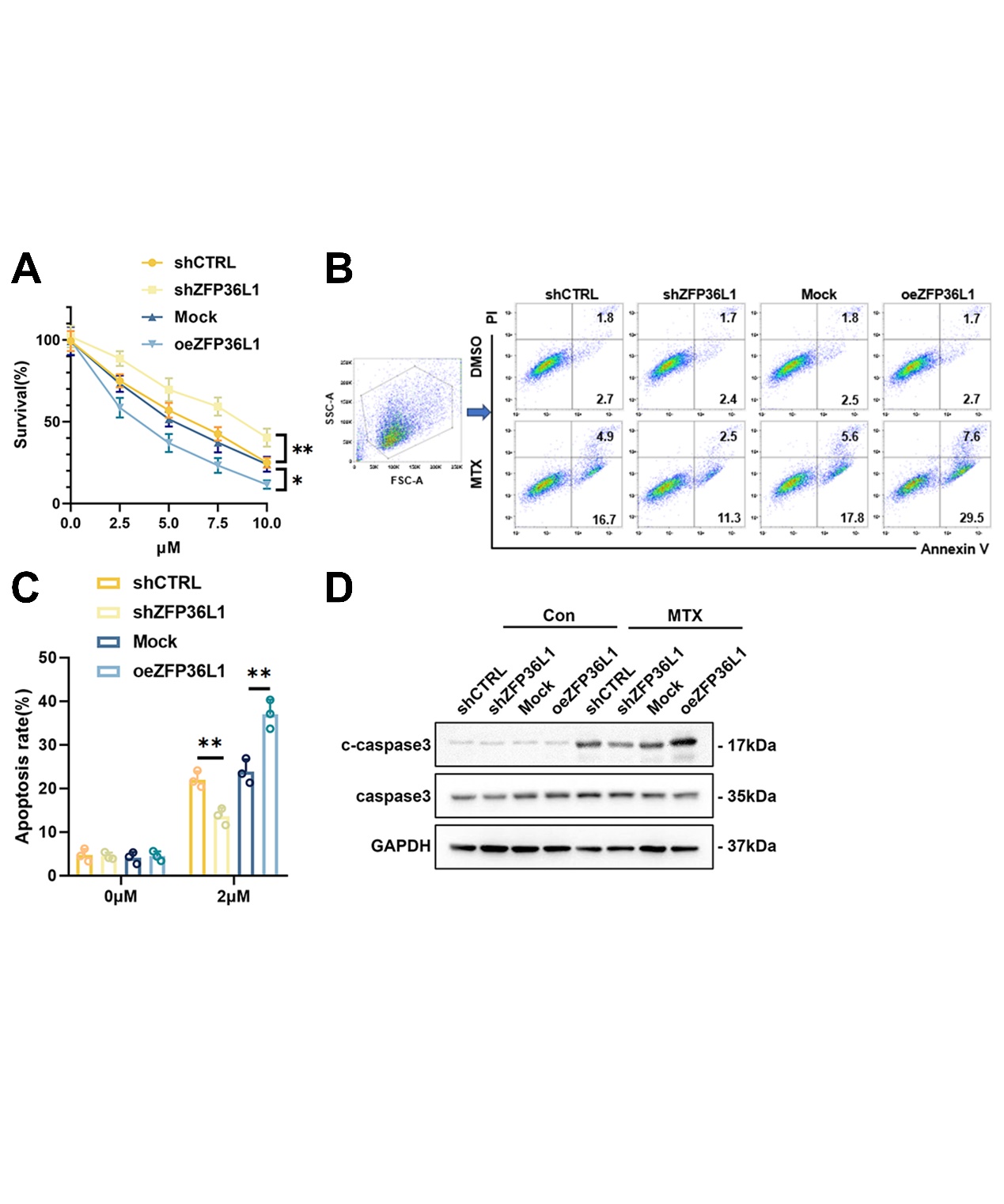
**

**Supplementary Figure 4**

**
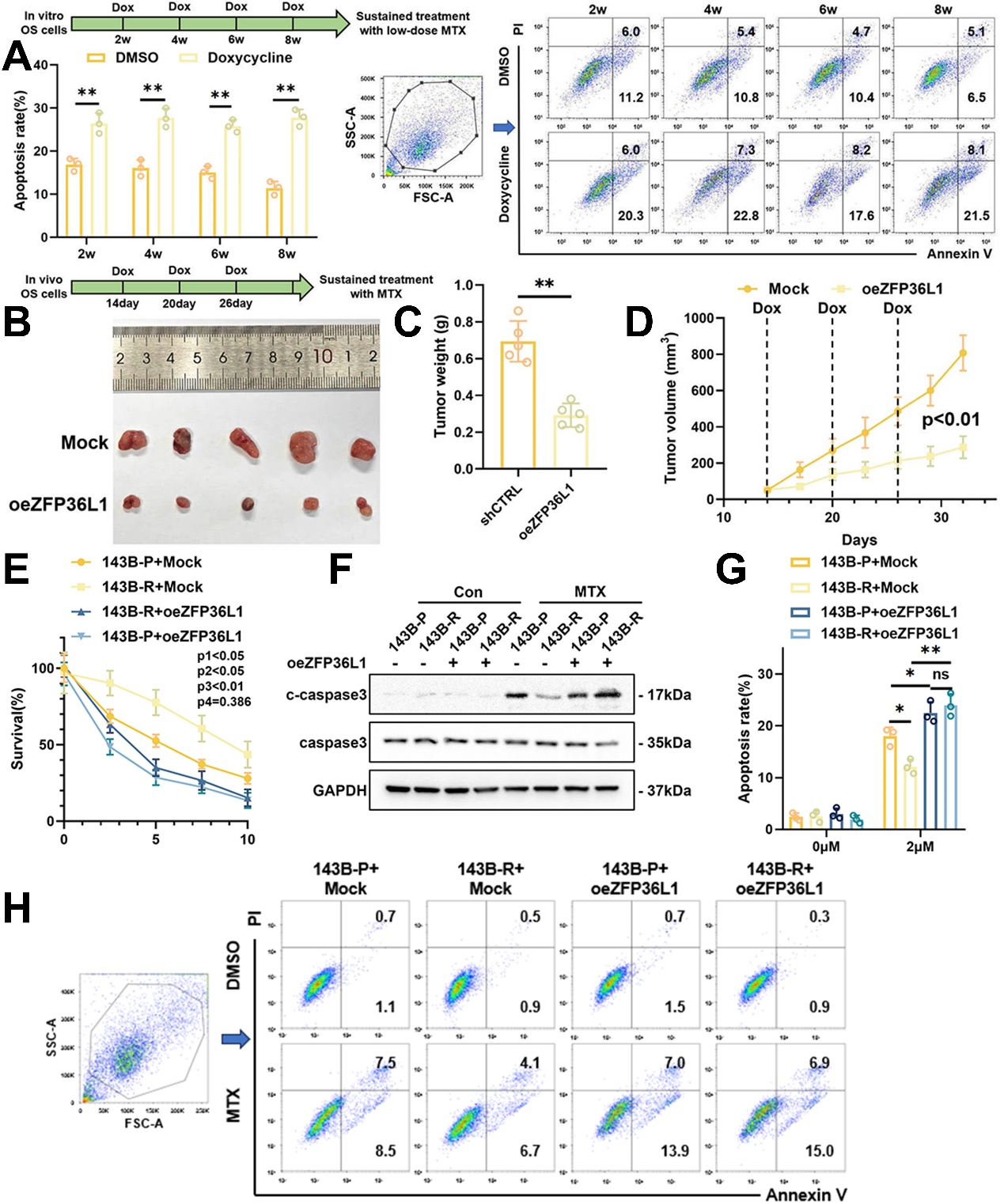
**

**Supplementary Figure 5**

**
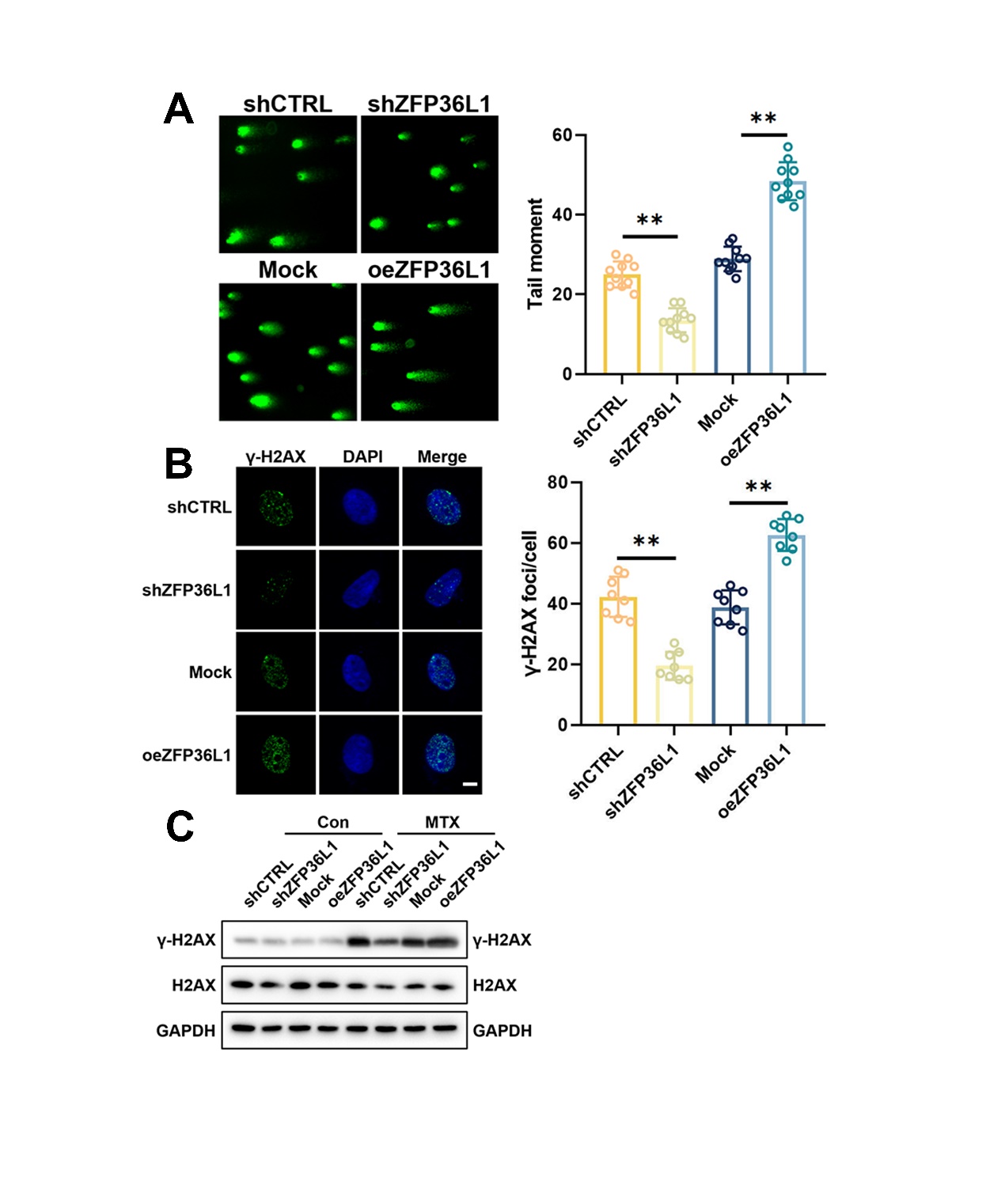
**

**Supplementary Figure 6**

**
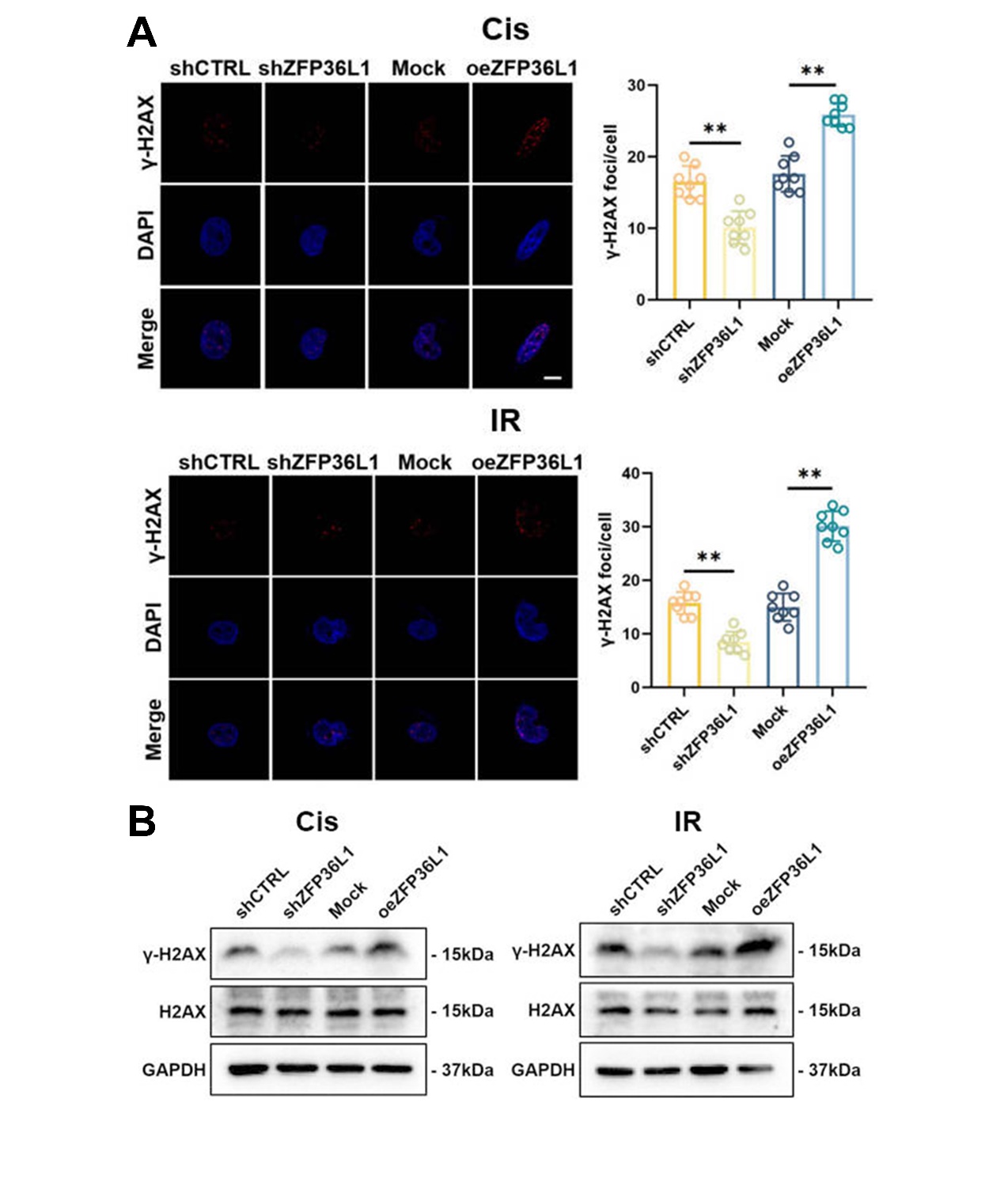
**

**Supplementary Figure 7**

**
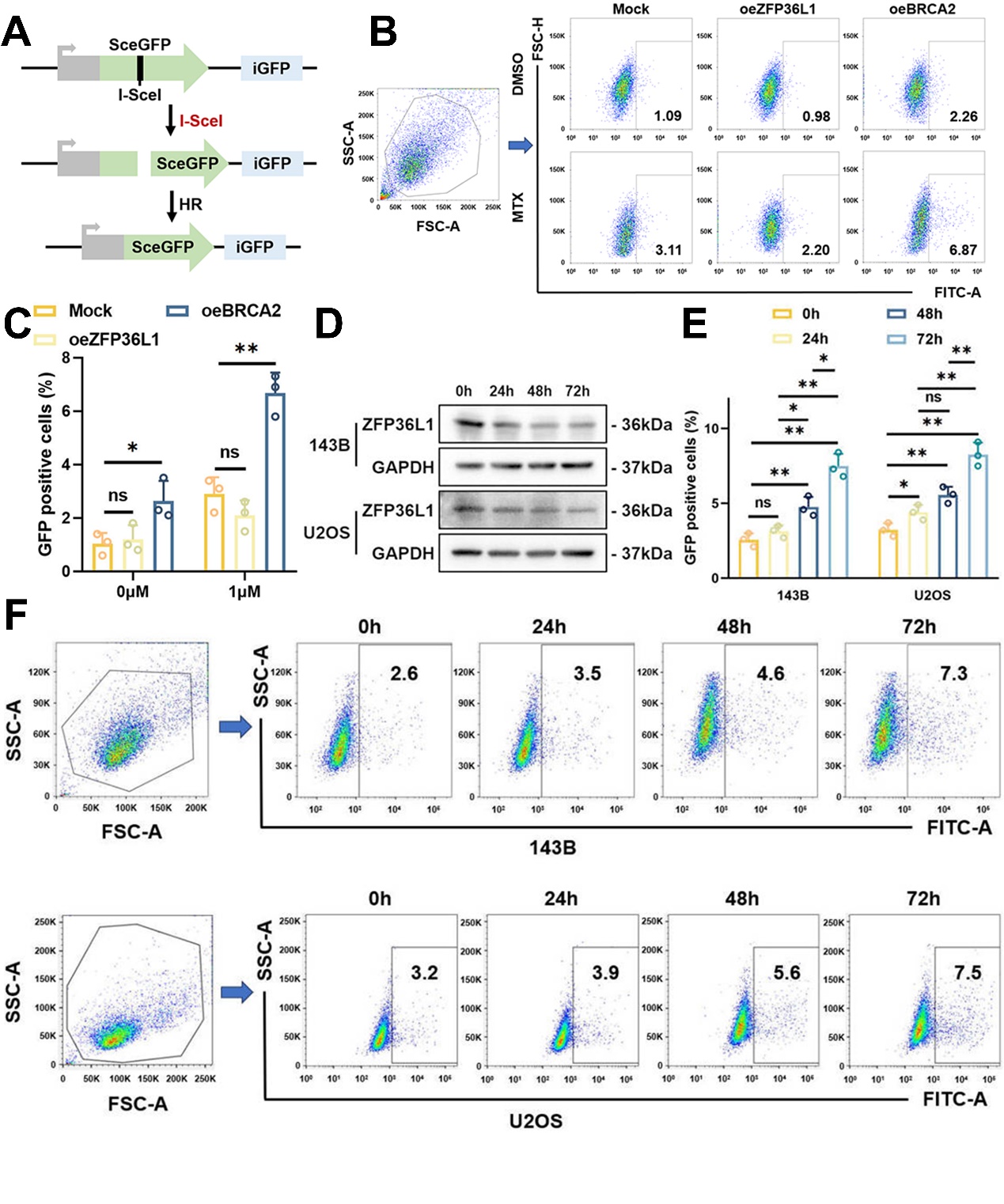
**

**Supplementary Figure 8**

**
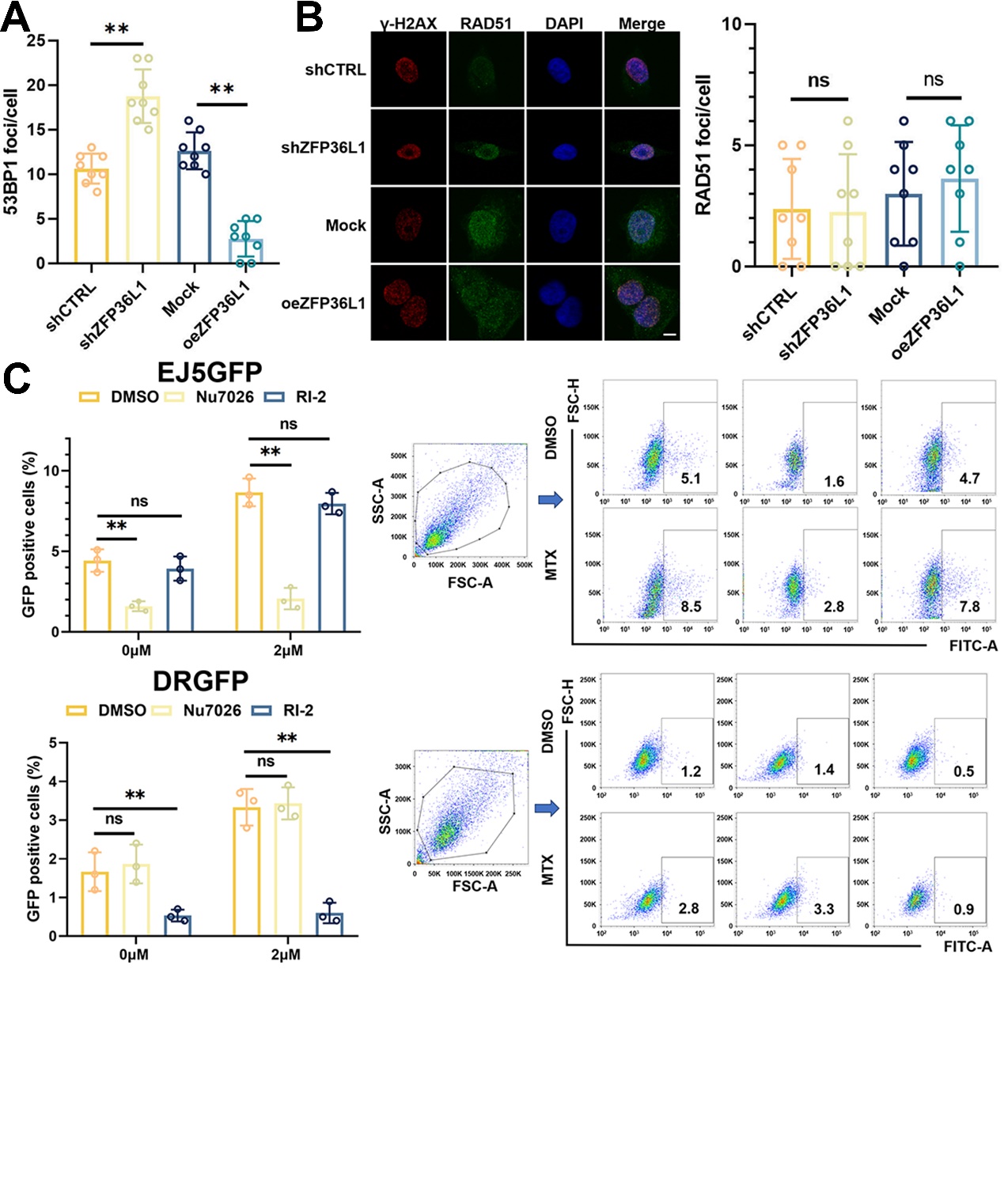
**

**Supplementary Figure 9**

**
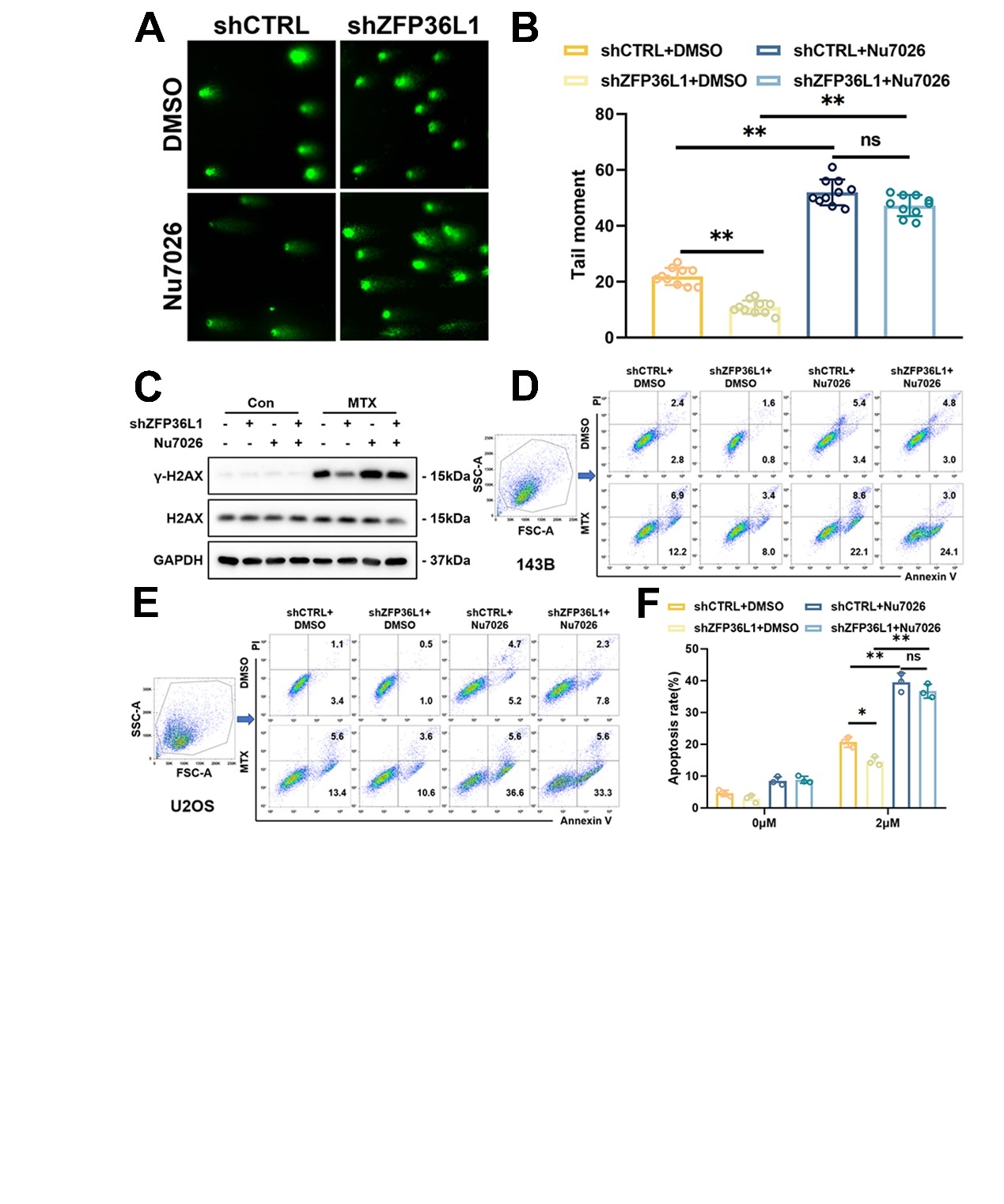
**

**Supplementary Figure 10**

**
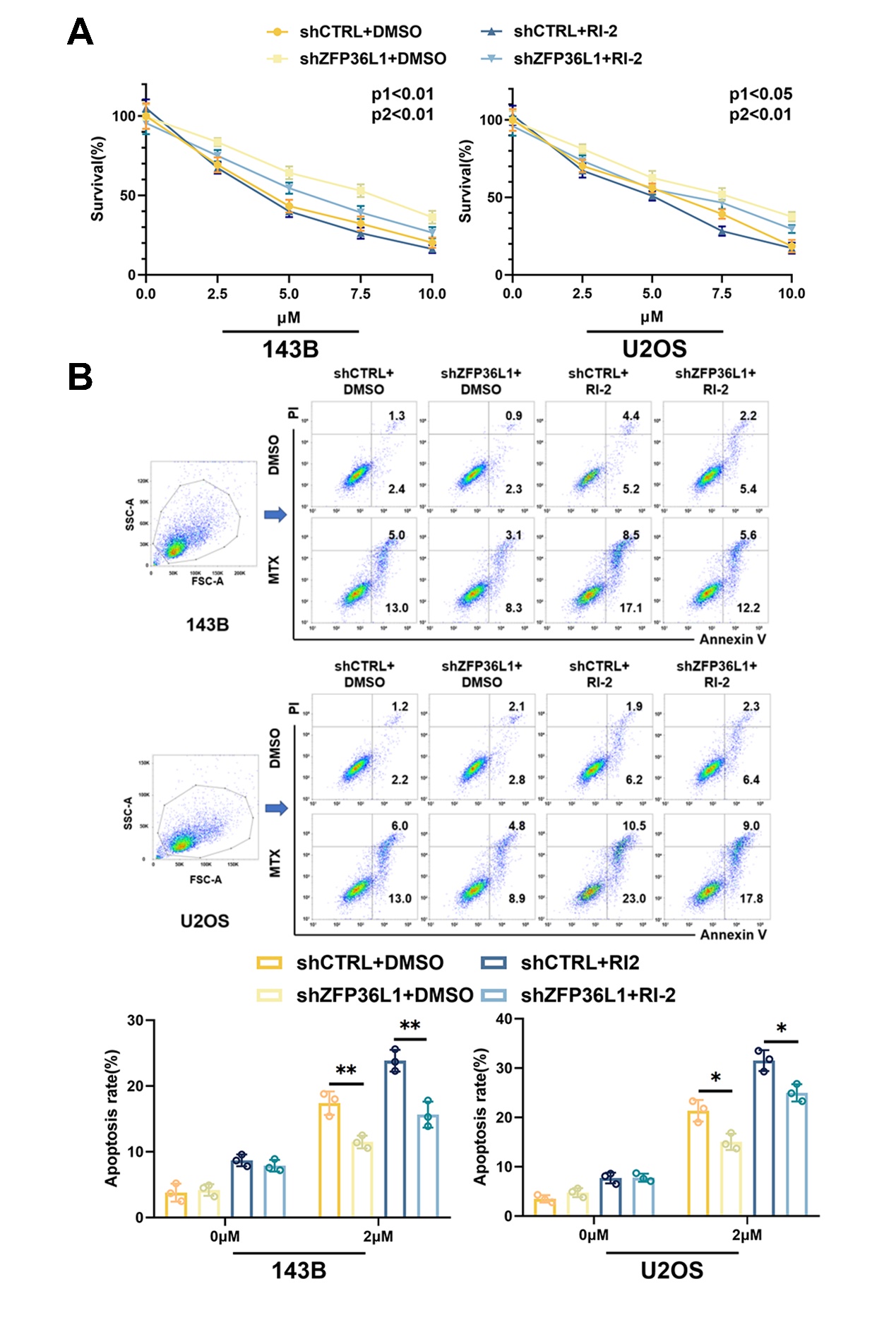
**

**Supplementary Figure 11**

**
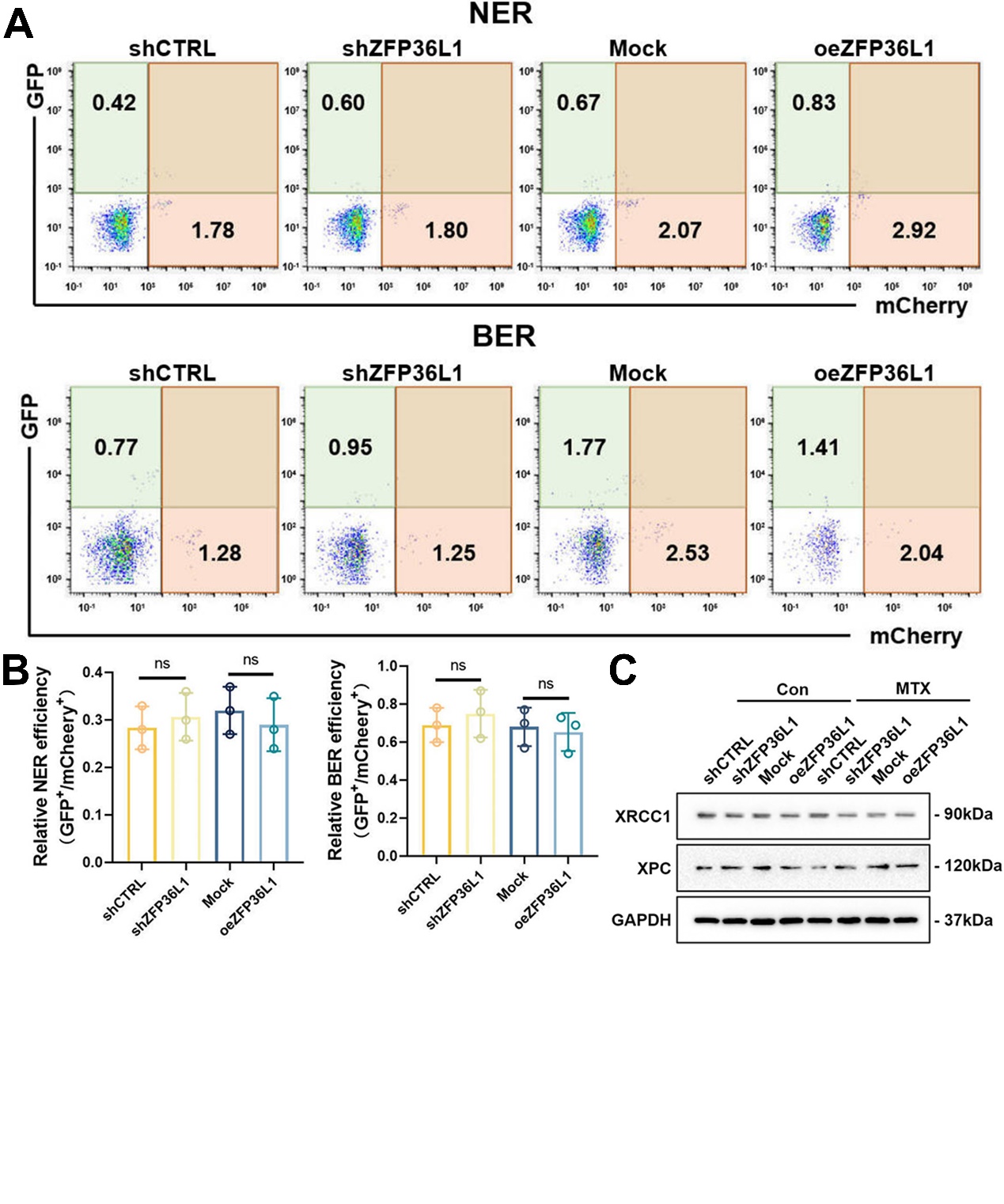
**

**Supplementary Figure 12**

**
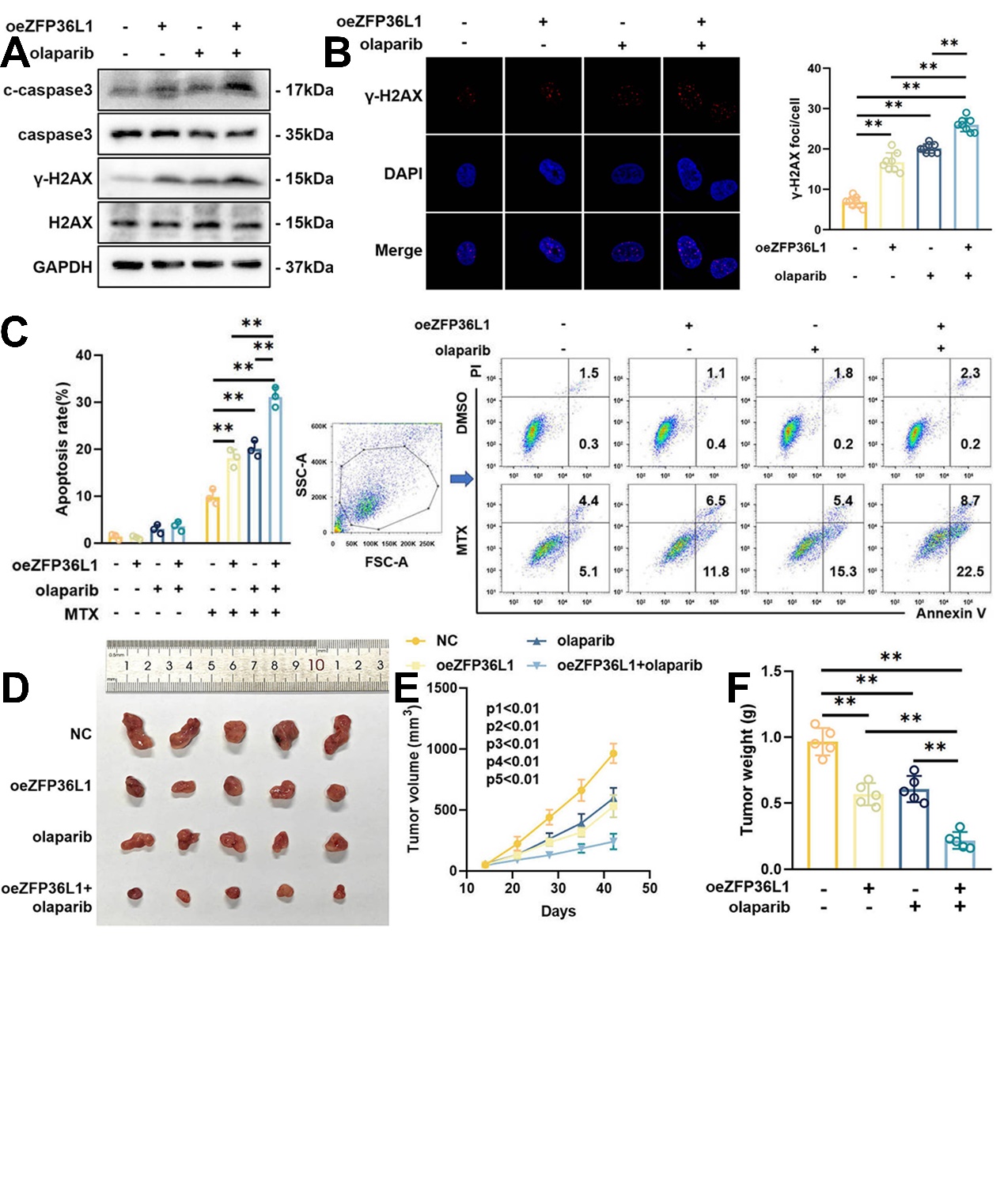
**

**Supplementary Figure 13**

**
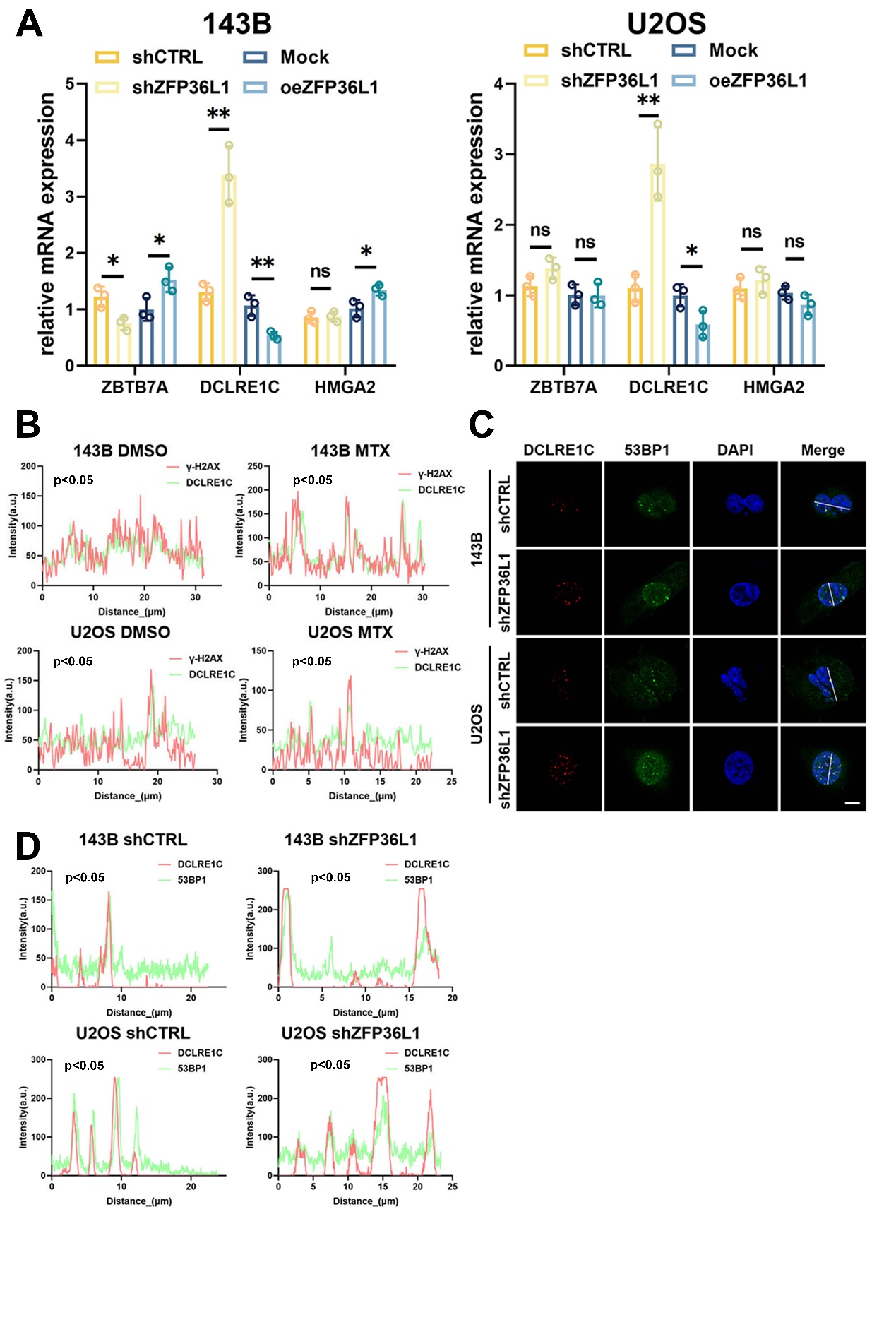
**

**Supplementary Figure 14**

**
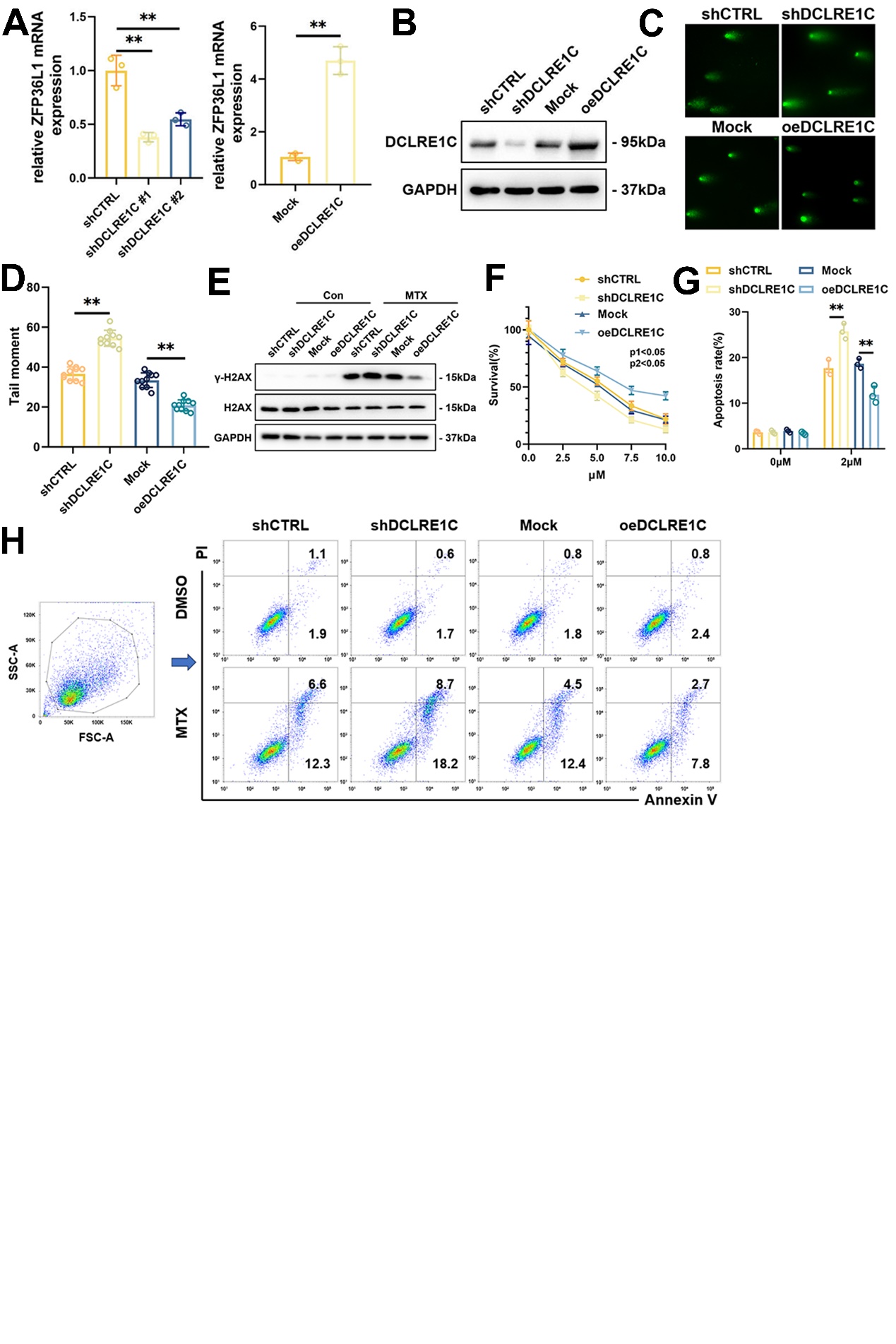
**

**Supplementary Figure 15**

**
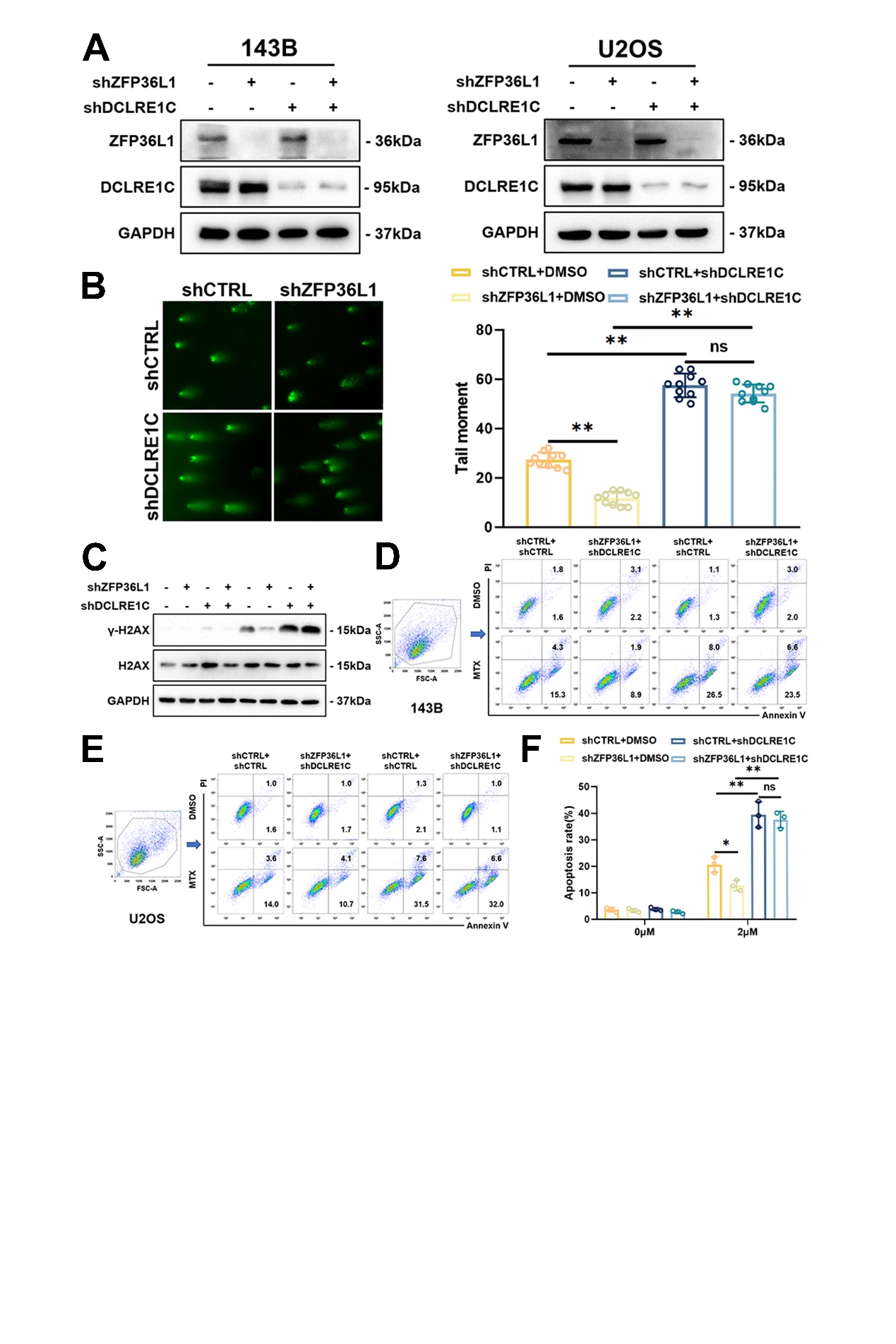
**

**Supplementary Figure 16**

**
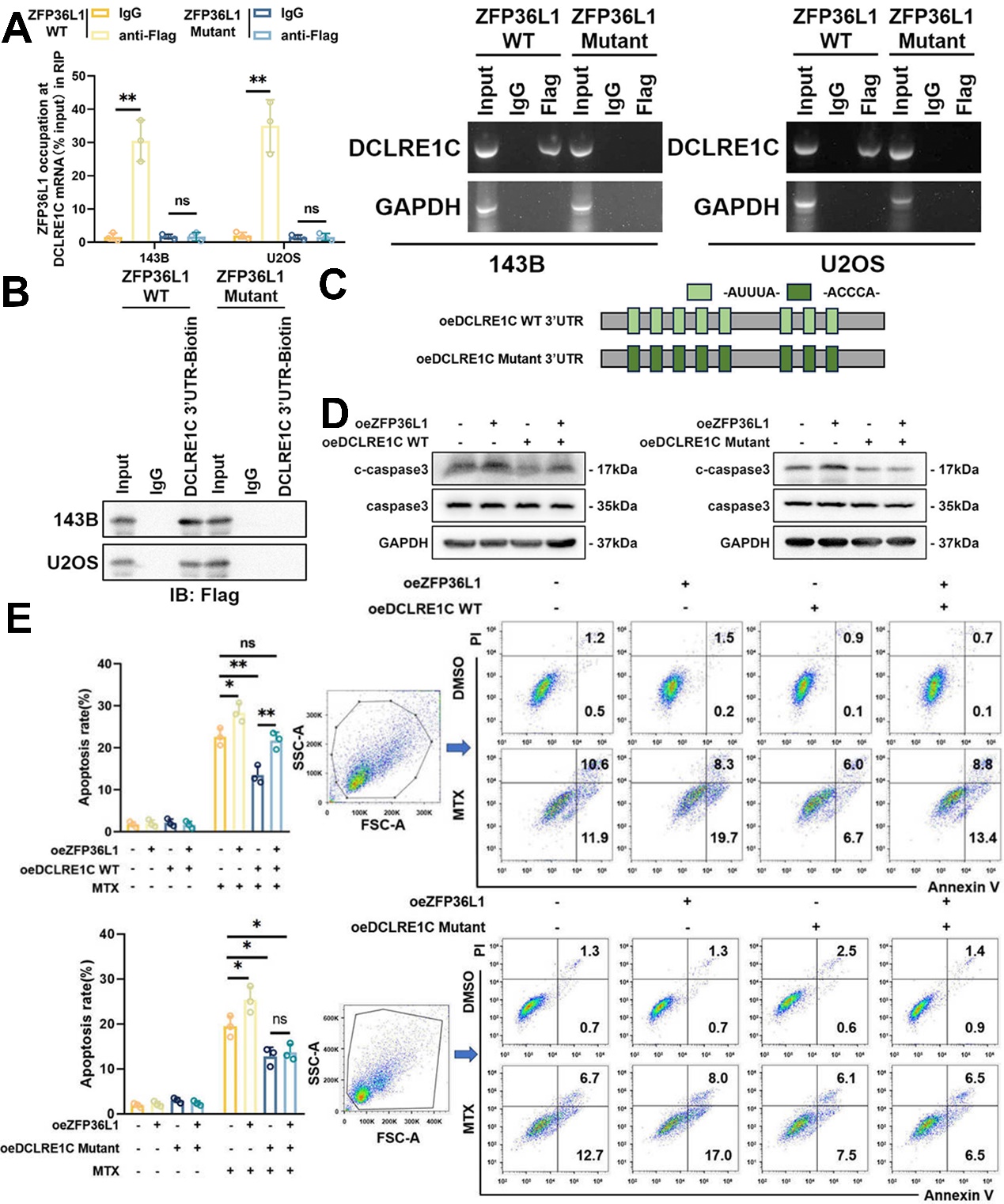
**

**Supplementary Figure 17**

**
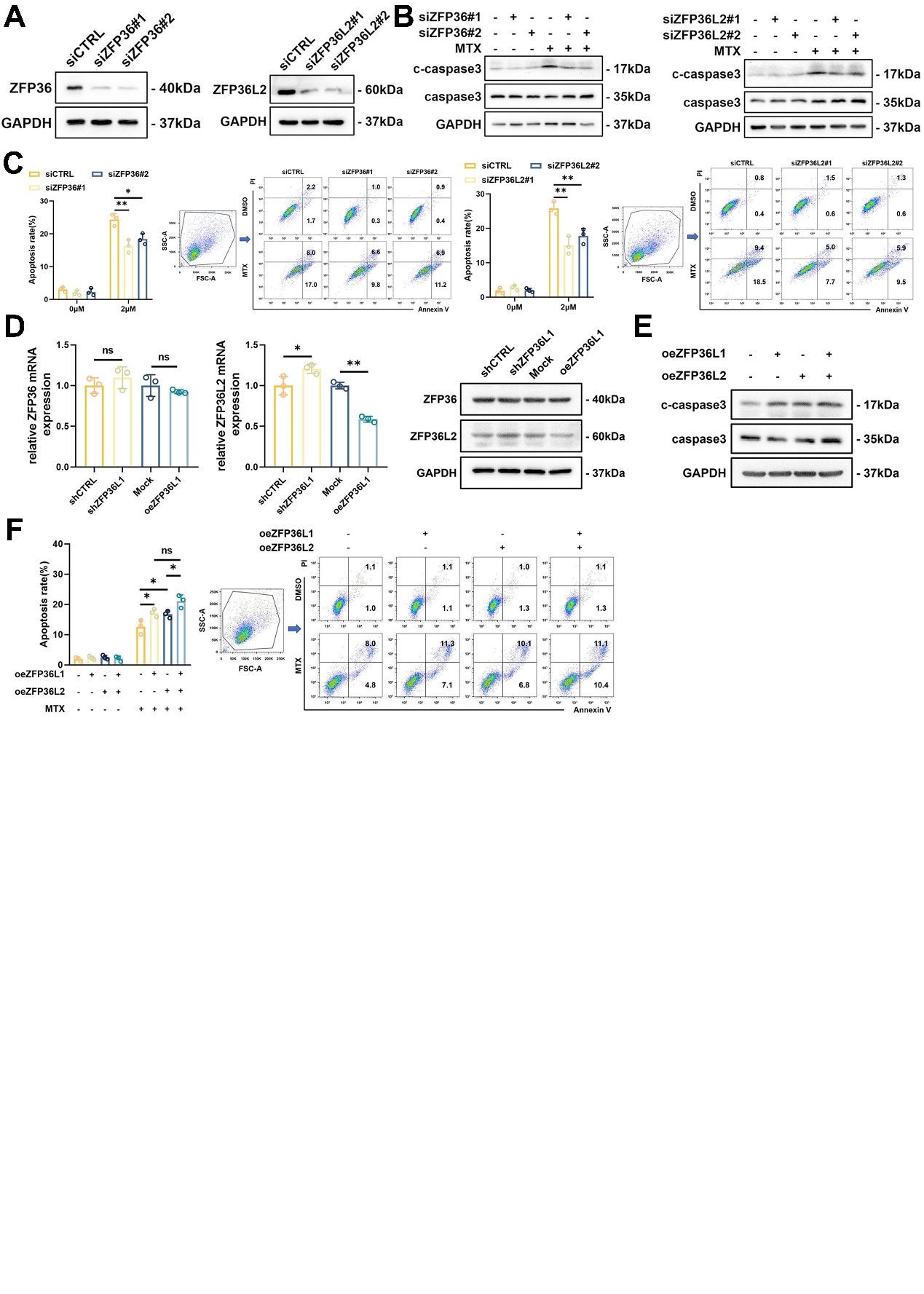
**

**Supplementary Figure 18**

**
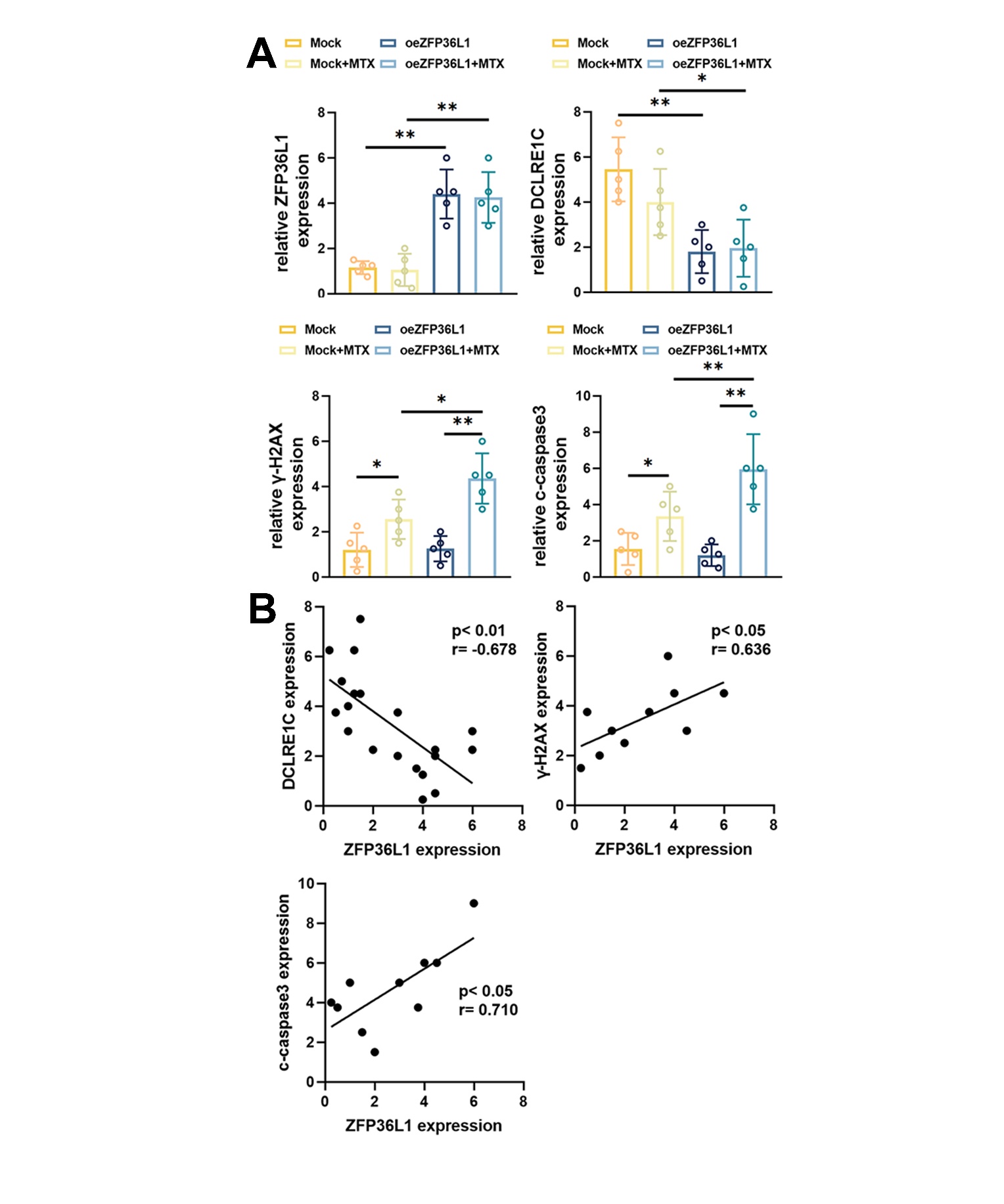
**

**Supplementary Figure 1. Construction of MTX-resistant 143B cells and ZFP36L1 knockdown or overexpression OS cell lines.**

A, CCK-8 assays were performed to measure the survival rate of 143B-P and 143B-R cells after MTX treatment. B-C, flow cytometry was performed to measure the proportion of apoptotic 143B-P and 143B-R cells after MTX treatment. D-E, qPCR (D) and WB (E) assays were performed to confirm the knockdown and overexpression efficiency of ZFP36L1 in 143B and U2OS cells. The data are shown as means ± SEMs; *p < 0.05, **p < 0.01.

**Supplementary Figure 2. ZFP36L1 exerts no effect on 143B growth in vivo.**

A-C, photographs (A) of xenograft tumors from mice injected with 143B cells expressing different levels of ZFP36L1. Tumor weights (B) and volumes (C) were measured. p1: shCTRL vs. shZFP36L1; p2: Mock vs. oeZFP36L1. The data are shown as means ± SEMs; *p < 0.05, **p < 0.01.

**Supplementary Figure 3. ZFP36L1 promotes apoptosis induced by MTX treatment in U2OS cells.**

A, CCK-8 assays were performed to measure the survival rate of U2OS cells after MTX treatment. B-C, flow cytometry was performed to measure the proportion of apoptotic U2OS cells after MTX treatment. D, WB assays were performed to detect the expression of apoptosis marker c-caspase3 in U2OS cells after MTX treatment. The data are shown as means ± SEMs; *p < 0.05, **p < 0.01.

**Supplementary Figure 4. ZFP36L1 shows sustained therapeutic effect in long-term MTX treatment.**

A, in vitro experiments, 143B cells were continuously treated with low-dose MTX, and doxycycline (Dox) was added to induce ZFP36L1 overexpression at indicated time points. Flow cytometry was performed to measure the proportion of apoptotic 143B cells at indicated time points. B-D, in vivo experiments, 143B cells were subcutaneously injected into nude mice to establish xenograft models. The mice underwent continuous MTX treatment, and Dox was administered at specific time points to induce ZFP36L1 overexpression within the tumors. After treatment, mice were euthanized, and the photograph (B) of xenograft tumors was captured. Tumor weights (C) and volumes (D) were measured. E, CCK-8 assays were performed to measure the survival rate of 143B-R cells after MTX treatment. p1: 143B-P + Mock vs. 143B-R+ Mock; p2: 143B-P+ Mock vs. 143B-P + oeZFP36L1; p3: 143B-R+ Mock vs. 143B-R + oeZFP36L1; p4: 143B-P+ oeZFP36L1 vs. 143B-R + oeZFP36L1. F, WB assays were performed to detect the expression of apoptosis marker c-caspase3 in 143B-R cells after MTX treatment. G-H, flow cytometry was performed to measure the proportion of apoptotic 143B-P and 143B-R cells after MTX treatment. The data are shown as means ± SEMs; *p < 0.05, **p < 0.01.

**Supplementary Figure 5. ZFP36L1 promotes DSBs induced by MTX treatment in U2OS cells.**

A, comet assays were performed to detect the level of DNA damage in U2OS cells after MTX treatment. B, IF assays were performed to detect the formation of γ-H2AX foci in the nucleus of U2OS cells after MTX treatment, and the number of γ-H2AX foci in each cell was quantified. Scale bar, 10μm. C, WB assays were performed to detect the expression of γ-H2AX in U2OS cells after MTX treatment. The data are shown as means ± SEMs; *p < 0.05, **p < 0.01.

**Supplementary Figure 6. ZFP36L1 promotes DSBs induced by Cis and IR treatment in OS cells.**

A, IF assays were performed to detect the formation of γ-H2AX foci in the nucleus of OS cells after Cis or IR treatment, and the number of γ-H2AX foci in each cell was quantified. Scale bar, 10μm. B, WB assays were performed to detect the expression of γ-H2AX in OS cells after Cis or IR treatment. The data are shown as means ± SEMs; *p < 0.05, **p < 0.01.

**Supplementary Figure 7. Effect of ZFP36L1 on NHEJ and HR pathways.**

A, schematic representation of the DR-GFP plasmid for measuring the capability of HR repair. B-C, flow cytometry was performed to measure the proportion of GFP positive cells in HR reporter assays (B). The percentage of GFP positive cells was quantified (C). D, WB assays were performed to detect the expression of ZFP36L1 in OS cells after MTX treatment for different durations. E-F, flow cytometry was performed to measure the proportion of GFP positive cells in NHEJ reporter assays. The data are shown as means ± SEMs; *p < 0.05, **p < 0.01.

**Supplementary Figure 8. The effect of Nu7026 and RI-2 on NHEJ and HR pathways.**

A, the number of 53BP1 foci in each cell was quantified in Fig 3D. B, IF assays were performed to detect the formation of RAD51 foci in the nucleus of OS cells after MTX treatment, and the number of RAD51 foci in each cell was quantified. Scale bar, 10μm. C-D, NHEJ and HR reporter assays were performed to measure the effect of Nu7026 and RI-2 on NHEJ and HR pathway. The data are shown as means ± SEMs; *p < 0.05, **p < 0.01.

**Supplementary Figure 9. ZFP36L1 inhibited DSBs repair through blocking the NHEJ pathway in U2OS cells.**

A-B, comet assays were performed to detect the level of DNA damage in U2OS cells after MTX treatment with or without Nu7026 treatment and the tail moment was quantified. C, WB assays were performed to detect the expression of γ-H2AX in U2OS cells after MTX treatment with or without Nu7026 treatment. D-F, flow cytometry was performed to measure the proportion of apoptotic 143B (D) and U2OS (E) cells after MTX treatment with or without Nu7026 treatment. The percentage of apoptotic U2OS cells was quantified (F). The data are shown as means ± SEMs; *p < 0.05, **p < 0.01.

**Supplementary Figure 10. Effect of the HR pathway on MTX resistance induced by ZFP36L1 knockdown.**

A, CCK-8 assays were performed to measure the survival rate of OS cells after MTX treatment with or without RI-2 treatment. p1: shCTRL vs. shZFP36L1; p2: shCTRL+RI-2 vs. shZFP36L1+RI-2. B, flow cytometry was performed to measure the proportion of apoptotic 143B and U2OS cells after MTX treatment with or without RI-2 treatment. The data are shown as means ± SEMs; *p < 0.05, **p < 0.01.

**Supplementary Figure 11. Minimal impact of ZFP36L1 on NER and BER pathways.**

A-B, flow cytometry was employed to quantify the ratio of GFP positive cells to mCherry positive cells in NER and BER reporter assays, respectively. C, WB assays were performed to detect key proteins in the NER and BER pathways in OS cells with differential ZFP36L1 expression after MTX treatment. The data are shown as means ± SEMs; *p < 0.05, **p < 0.01.

**Supplementary Figure 12. ZFP36L1 overexpression combined with olaparib potentiates MTX sensitivity in OS.**

A, WB assays were performed to detect the expression of c-caspase3 and γ-H2AX in 143B cells after MTX treatment. B, IF assays were performed to detect the formation of γ-H2AX foci in the nucleus of 143B cells after MTX treatment, and the number of γ-H2AX foci in each cell was quantified. Scale bar, 10μm. C, flow cytometry was performed to measure the proportion of apoptotic 143B cells after MTX treatment. D-F, photographs (D) of xenograft tumors from mice injected with 143B cells after MTX treatment. Tumor weights (E) and volumes (F) were measured. p1: NC vs. oeZFP36L1; p2: NC vs. olaparib; p3: NC vs. oeZFP36L1 + olaparib; p4: oeZFP36L1 vs. oeZFP36L1 + olaparib; p5: olaparib vs. oeZFP36L1 + olaparib. The data are shown as means ± SEMs; *p < 0.05, **p < 0.01.

**Supplementary Figure 13. The relationship between ZFP36L1 and DCLRE1C in OS cells.**

A, qPCR assays were performed to detect the expression of ZBTB7A, DCLRE1C and HMGA2 in OS cells. B, the colocalization of DCLRE1C and γ-H2AX in OS cells was quantified in Fig 4E. C-D, IF assays were performed to detect the colocalization of DCLRE1C and 53BP1 in shZFP36L1 OS cells. Scale bar, 10μm. The data are shown as means ± SEMs; *p < 0.05, **p < 0.01.

**Supplementary Figure 14. DCLRE1C inhibited apoptosis and DSBs induced by MTX treatment in 143B cells.**

A-B, qPCR (A) and WB (B) assays were performed to confirm the knockdown and overexpression efficiency of DCLRE1C in 143B cells. C-D, comet assays were performed to detect the level of DNA damage in 143B cells after MTX treatment and tail moment was quantified. E, WB assays were performed to detect the expression of γ-H2AX in 143B cells after MTX treatment. F, CCK-8 assays were performed to measure the survival rate of 143B cells after MTX treatment. p1: shCTRL vs. shDCLRE1C; p2: Mock vs. oeDCLRE1C. G-H, flow cytometry was performed to measure the proportion of apoptotic 143B cells after MTX treatment. The data are shown as means ± SEMs; *p < 0.05, **p < 0.01.

**Supplementary Figure 15. Effect of DCLRE1C on MTX resistance induced by ZFP36L1 knockdown.**

A, WB assays were performed to detect the knockdown efficiency of ZFP36L1 and DCLRE1C in rescue assays. B, comet assays were performed to detect the level of DNA damage in U2OS cells after MTX treatment and tail moment was quantified. C, WB assays were performed to detect the expression of γ-H2AX in U2OS cells after MTX treatment. D-F, flow cytometry was performed to measure the proportion of apoptotic 143B (D) and U2OS cells (E) after MTX treatment. The percentage of apoptotic U2OS cells was quantified (F). The data are shown as means ± SEMs; *p < 0.05, **p < 0.01.

**Supplementary Figure 16. The interaction between ZFP36L1 and DCLRE1C mRNA plays a crucial role in ZFP36L1 mediated MTX sensitivity.**

A, RIP assays were performed to detect the binding relationship between ZFP36L1 WT-Flag or ZFP36L1 Mutant-Flag and DCLRE1C mRNA in OS cells. B, RNA pull-down assays were performed to detect the role of 3’UTR of DCLRE1C mRNA in the binding relation between ZFP36L1 WT-Flag or ZFP36L1 Mutant-Flag and DCLRE1C mRNA. C, schematic representation of the 3’UTR of DCLRE1C mRNA (NM_001033855.3) in DCLRE1C WT or DCLRE1C Mutant overexpression plasmids. D-E, WB assays and flow cytometry were performed to detect the apoptotic levels of 143B cells transfected with DCLRE1C WT or DCLRE1C Mutant overexpression plasmid after MTX treatment. The data are shown as means ± SEMs; *p < 0.05, **p < 0.01.

**Supplementary Figure 17. ZFP36 and ZFP36L2 promote apoptosis induced by MTX treatment in 143B cells.**

A, WB assays were performed to detect the expression of ZFP36 or ZFP36L2 in 143B cells. B, WB assays were performed to detect the expression of apoptosis marker c-caspase3 in 143B cells after MTX treatment. C, flow cytometry was performed to measure the proportion of apoptotic 143B cells after MTX treatment. D, qPCR and WB assays were performed to detect the expression of ZFP36 or ZFP36L2 in 143B cells. E, WB assays were performed to detect the expression of apoptosis marker c-caspase3 in 143B cells after MTX treatment. F, flow cytometry was performed to measure the proportion of apoptotic 143B cells after MTX treatment. The data are shown as means ± SEMs; *p < 0.05, **p < 0.01.

**Supplementary Figure 18. Quantitative Analysis of IHC Staining in the lung metastatic lesions.**

A, relative expression levels of ZFP36L1, DCLRE1C, γ-H2AX and c-caspase3 were quantified across different groups of lung metastatic lesions using IHC staining. B, Linear correlation analyses were performed to evaluate the relationships between ZFP36L1 and DCLRE1C in various groups. Additionally, correlations between ZFP36L1 and γ-H2AX, and between ZFP36L1 and c-caspase3, were analyzed in the MTX treatment groups based on IHC staining of xenograft tissues. The data are shown as means ± SEMs; *p < 0.05, **p < 0.01.
