# Supplementary material for: Downregulation of ZFP36L1 contributes to methotrexate resistance in osteosarcoma through enhanced NHEJ DNA repair mechanisms
Source: Cell Death Dis. 2025 Nov 24;16(1):852. doi: 10.1038/s41419-025-08217-4 (PMC12644584; doi:10.1038/s41419-025-08217-4)
Supplement: Supplementary file 2 — supplementary materials tables [file 41419_2025_8217_MOESM2_ESM.docx]

**Table S1. Primer sequences used for qRT-PCR assays**

| Genes | Primer |
| --- | --- |
| ZFP36L1-F | TCCAGCATAGCTTTAGCTTTGC |
| ZFP36L1-R | GGTCATCGGCGCTCAGAATAG |
| ZBTB7A-F | GCCCACAACTACGACCTGAA |
| ZBTB7A-R | TGTGAAGTTACCGTCGGTGG |
| DCLRE1C-F | GCAGATGGCCGAGTATCCAA |
| DCLRE1C-R | ATGTCTTTGACTCTGCCCCC |
| HMGA2-F | TTGGTGCAAGACTCAGGAGC |
| HMGA2-R | CAGCACCTTTCGGGAGATGA |
| GAPDH-F | GGAGCGAGATCCCTCCAAAAT |
| GAPDH-R | GGCTGTTGTCATACTTCTCATGG |

**Table S2. Primer sequences used for RIP assays**

| Genes | Primer |
| --- | --- |
| DCLRE1C-F | GGTGGCCAAATTAGAAGCGG |
| DCLRE1C-R | AGCCTAACACATTGATGGACTT |
| GAPDH-F | GGAGCGAGATCCCTCCAAAAT |
| GAPDH-R | GGCTGTTGTCATACTTCTCATGG |

**Table S3. Clinicopathological characteristics of 37 patients with osteosarcoma**

| **Characteristics** | **All patients** | **Expression of ZFP36L1** | | **p value** |
| --- | --- | --- | --- | --- |
|  |  | **Low (n=21)** | **High (n=16)** |  |
| **Sex** | p=0.2913 | | | |
| Male | 25 | 16 | 9 |  |
| Female | 12 | 5 | 7 |  |
| **Age (years)** | p=0.7486 | | | |
| ＜14 | 15 | 8 | 7 |  |
| ≥14 | 22 | 13 | 9 |  |
| **Anatomical site** | p=0.2893 | | | |
| Femur | 19 | 11 | 8 |  |
| Tibia | 7 | 2 | 5 |  |
| Humerus | 10 | 7 | 3 |  |
| Pelvis | 1 | 1 | 0 |  |

**Table S4. List of ZFP36L1 Expression Levels and Survival Data for Patients in the TARGET-OS Database**

| **Case ID** | **ZFP36L1 tpm** | **Vital Status** | **Overall Survival Time in Days** |
| --- | --- | --- | --- |
| **TARGET-40-PASUUH** | **46.3233** | **Alive** | **1625** |
| **TARGET-40-PAKFVX** | **69.9724** | **Alive** | **922** |
| **TARGET-40-PAMLKS** | **71.667** | **Dead** | **180** |
| **TARGET-40-PAPKWD** | **83.2441** | **Dead** | **422** |
| **TARGET-40-PANZZJ** | **89.862** | **Alive** | **1538** |
| **TARGET-40-PAVALD** | **92.487** | **Alive** | **551** |
| **TARGET-40-0A4I8U** | **97.7935** | **Alive** | **983** |
| **TARGET-40-PAUYTT** | **98.057** | **Alive** | **579** |
| **TARGET-40-PAUTYB** | **100.3401** | **Dead** | **537** |
| **TARGET-40-PAPWWC** | **107.2381** | **Alive** | **2520** |
| **TARGET-40-PARBGW** | **122.8359** | **Alive** | **286** |
| **TARGET-40-PAMYYJ** | **126.0128** | **Dead** | **271** |
| **TARGET-40-PANXSC** | **135.2729** | **Alive** | **1217** |
| **TARGET-40-PANZHX** | **136.2048** | **Alive** | **1323** |
| **TARGET-40-PALECC** | **140.0511** | **Dead** | **754** |
| **TARGET-40-PASNZV** | **145.256** | **Alive** | **1747** |
| **TARGET-40-PATUXZ** | **146.7717** | **Dead** | **510** |
| **TARGET-40-PATMPU** | **148.6772** | **Dead** | **1579** |
| **TARGET-40-PASRNE** | **153.3431** | **Alive** | **1707** |
| **TARGET-40-PAUVUL** | **153.8803** | **Dead** | **347** |
| **TARGET-40-PAUXPZ** | **157.3176** | **Dead** | **606** |
| **TARGET-40-0A4HXS** | **159.4685** | **Alive** | **2948** |
| **TARGET-40-PAPNVD** | **160.6568** | **Dead** | **922** |
| **TARGET-40-PAKZZK** | **173.4393** | **Dead** | **1515** |
| **TARGET-40-PATMXR** | **181.0912** | **Dead** | **386** |
| **TARGET-40-PATKSS** | **189.7679** | **Dead** | **758** |
| **TARGET-40-PARDAX** | **193.5629** | **Dead** | **679** |
| **TARGET-40-PAVCLP** | **193.9561** | **Alive** | **511** |
| **TARGET-40-PALFYN** | **200.9044** | **Dead** | **1003** |
| **TARGET-40-PATPBS** | **204.0267** | **Alive** | **1468** |
| **TARGET-40-PALKGN** | **206.6459** | **Alive** | **2122** |
| **TARGET-40-0A4I4M** | **214.021** | **Alive** | **708** |
| **TARGET-40-PATEEM** | **215.8918** | **Alive** | **1451** |
| **TARGET-40-PAKXLD** | **217.202** | **Alive** | **2462** |
| **TARGET-40-PARGTM** | **224.4296** | **Alive** | **2610** |
| **TARGET-40-0A4I3S** | **225.1377** | **Alive** | **1844** |
| **TARGET-40-PAMHLF** | **240.3541** | **Alive** | **1913** |
| **TARGET-40-0A4I5B** | **246.5004** | **Alive** | **619** |
| **TARGET-40-PARKAF** | **246.5615** | **Dead** | **1061** |
| **TARGET-40-PASEBY** | **268.775** | **Alive** | **2049** |
| **TARGET-40-PASKZZ** | **268.8628** | **Alive** | **542** |
| **TARGET-40-PAMTCM** | **269.7805** | **Alive** | **3002** |
| **TARGET-40-PANMIG** | **272.2873** | **Dead** | **776** |
| **TARGET-40-PAMHYN** | **276.8132** | **Alive** | **3900** |
| **TARGET-40-PARFTG** | **294.7026** | **Dead** | **653** |
| **TARGET-40-PALZGU** | **299.7951** | **Dead** | **2882** |
| **TARGET-40-0A4I9K** | **304.5963** | **Alive** | **2257** |
| **TARGET-40-0A4I4O** | **318.3588** | **Dead** | **627** |
| **TARGET-40-PANVJJ** | **334.729** | **Alive** | **3095** |
| **TARGET-40-PAUUML** | **341.4888** | **Alive** | **672** |
| **TARGET-40-PASYUK** | **368.8845** | **Alive** | **1870** |
| **TARGET-40-0A4I42** | **372.6168** | **Dead** | **619** |
| **TARGET-40-PASEFS** | **390.807** | **Alive** | **2112** |
| **TARGET-40-PAPIJR** | **401.6577** | **Alive** | **1719** |
| **TARGET-40-PAUBIT** | **410.7699** | **Alive** | **1029** |
| **TARGET-40-PAMEKS** | **414.2056** | **Dead** | **857** |
| **TARGET-40-PATMIF** | **417.3705** | **Alive** | **1580** |
| **TARGET-40-PANSEN** | **480.3672** | **Alive** | **2256** |
| **TARGET-40-PASFCV** | **486.0778** | **Alive** | **2062** |
| **TARGET-40-PAPXGT** | **498.2247** | **Alive** | **2526** |
| **TARGET-40-0A4HLD** | **499.4329** | **Alive** | **3946** |
| **TARGET-40-0A4HX8** | **509.1416** | **Alive** | **1750** |
| **TARGET-40-0A4I0W** | **526.4917** | **Alive** | **683** |
| **TARGET-40-PATJVI** | **527.9892** | **Alive** | **1575** |
| **TARGET-40-PAUTWB** | **535.2162** | **Alive** | **687** |
| **TARGET-40-PARJXU** | **563.4084** | **Dead** | **1631** |
| **TARGET-40-PALWWX** | **567.3628** | **Alive** | **2163** |
| **TARGET-40-PASSLM** | **618.1034** | **Alive** | **1865** |
| **TARGET-40-0A4I6O** | **652.0584** | **Dead** | **1906** |
| **TARGET-40-PAVECB** | **680.4504** | **Alive** | **476** |
| **TARGET-40-0A4I48** | **692.1289** | **Alive** | **1616** |
| **TARGET-40-PATAWV** | **699.1253** | **Alive** | **1702** |
