# Supplementary material for: Downregulation of ZFP36L1 contributes to methotrexate resistance in osteosarcoma through enhanced NHEJ DNA repair mechanisms
Source: Cell Death Dis. 2025 Nov 24;16(1):852. doi: 10.1038/s41419-025-08217-4 (PMC12644584; doi:10.1038/s41419-025-08217-4)

Figure1 B

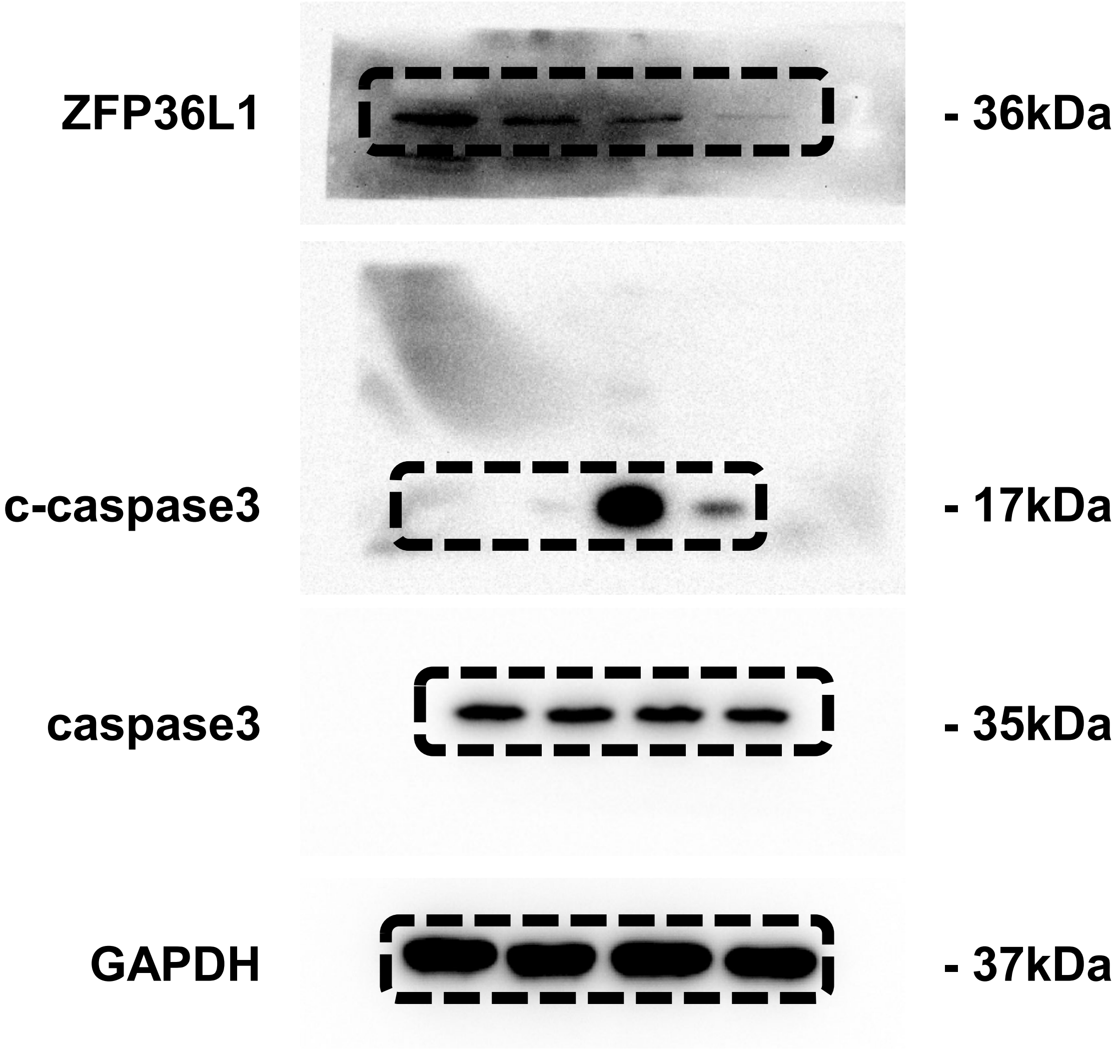

Figure1 H

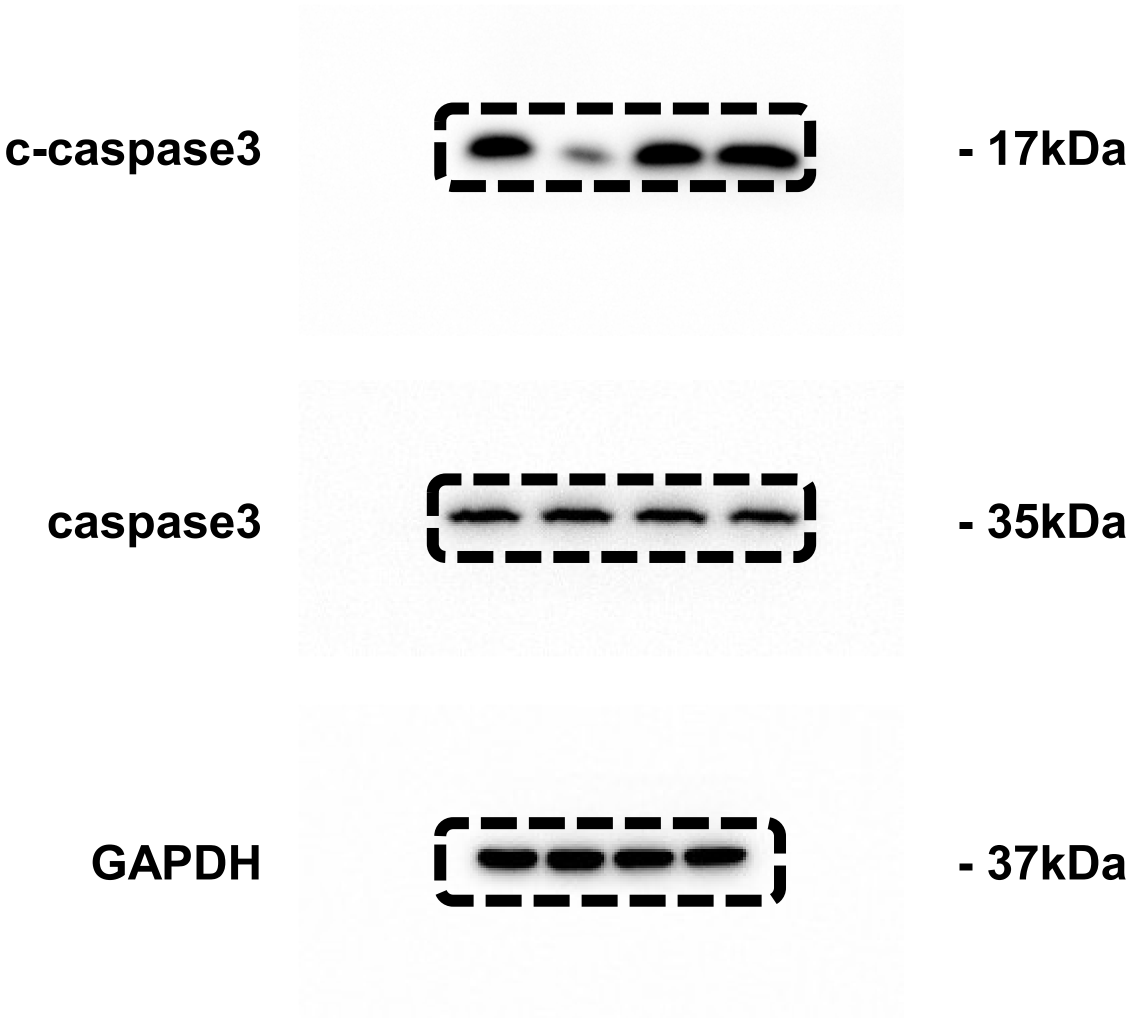

Figure2 D

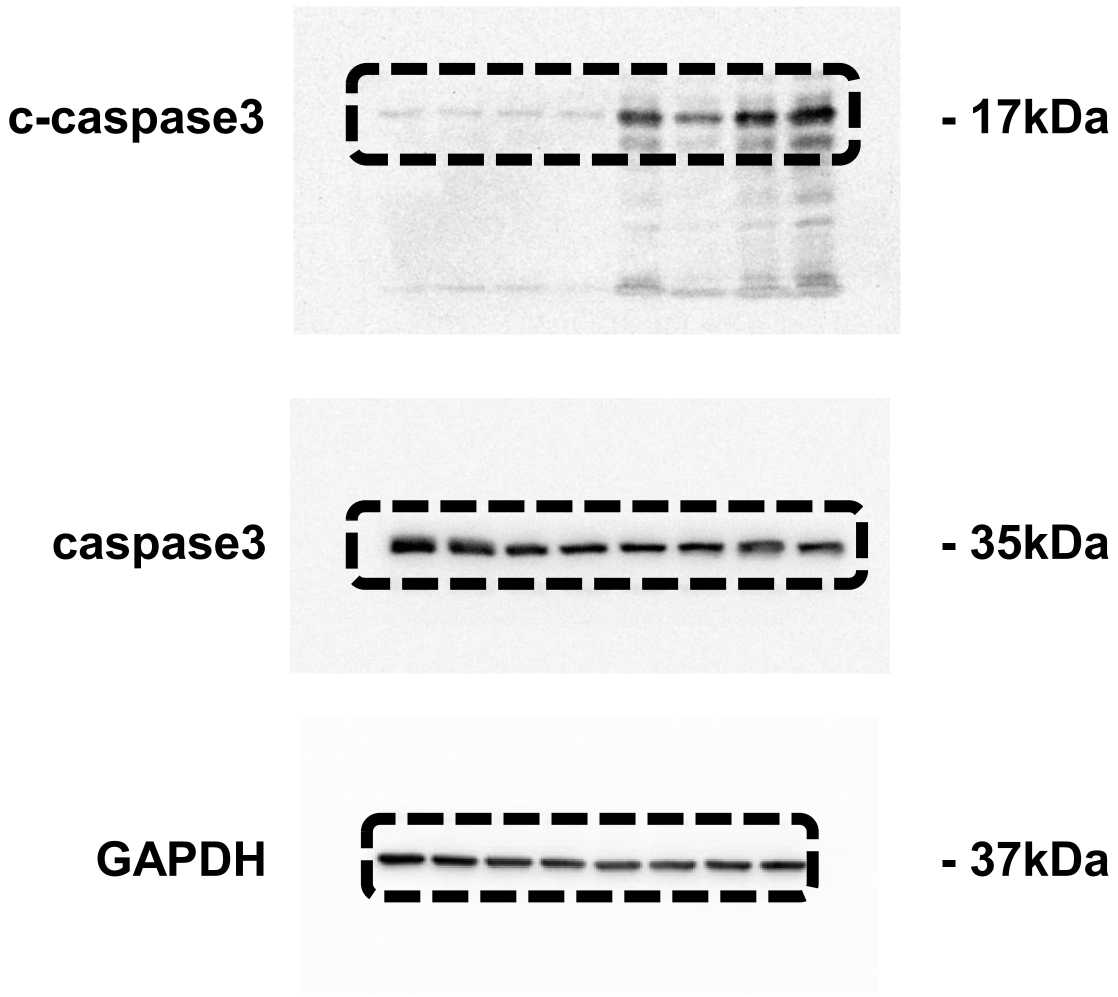

Figure2 H

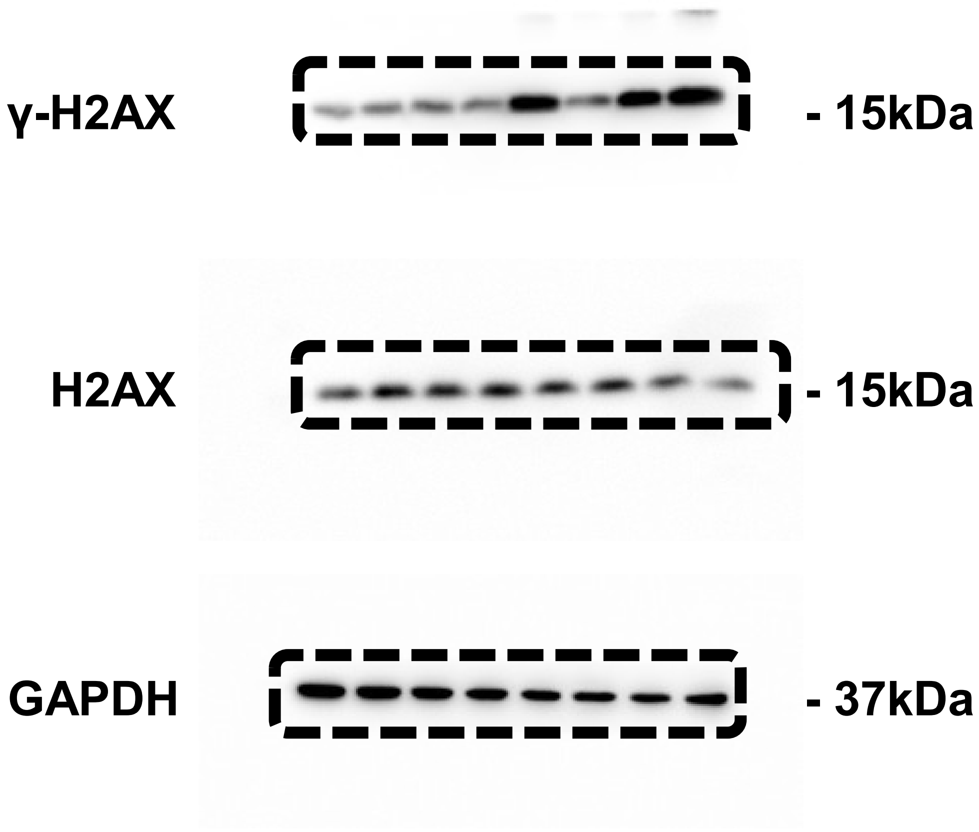

Figure3 A

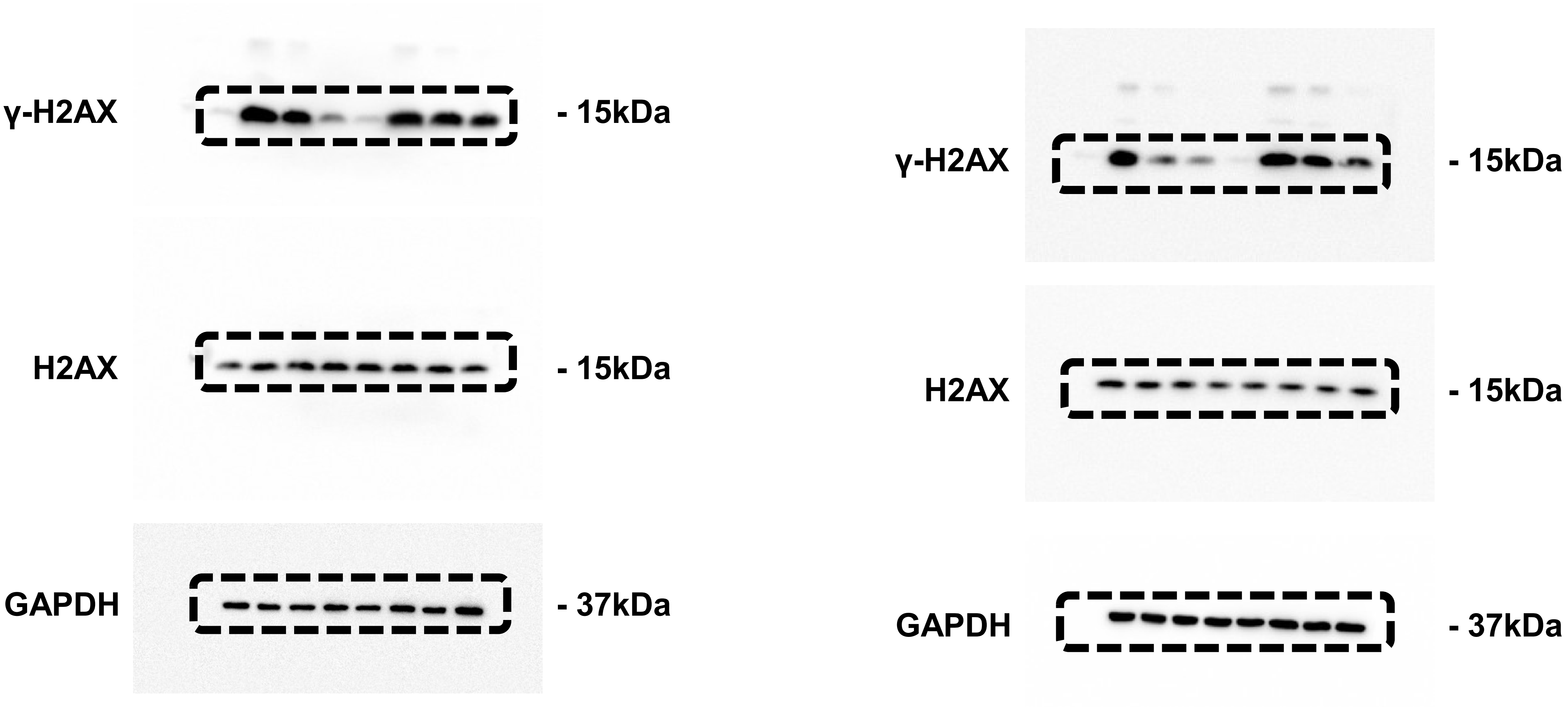
γ-H2AX
- 15kDa
H2AX
- 15kDa
GAPDH
- 37kDa
γ-H2AX
- 15kDa
H2AX
- 15kDa
GAPDH
- 37kDa

Figure3 G

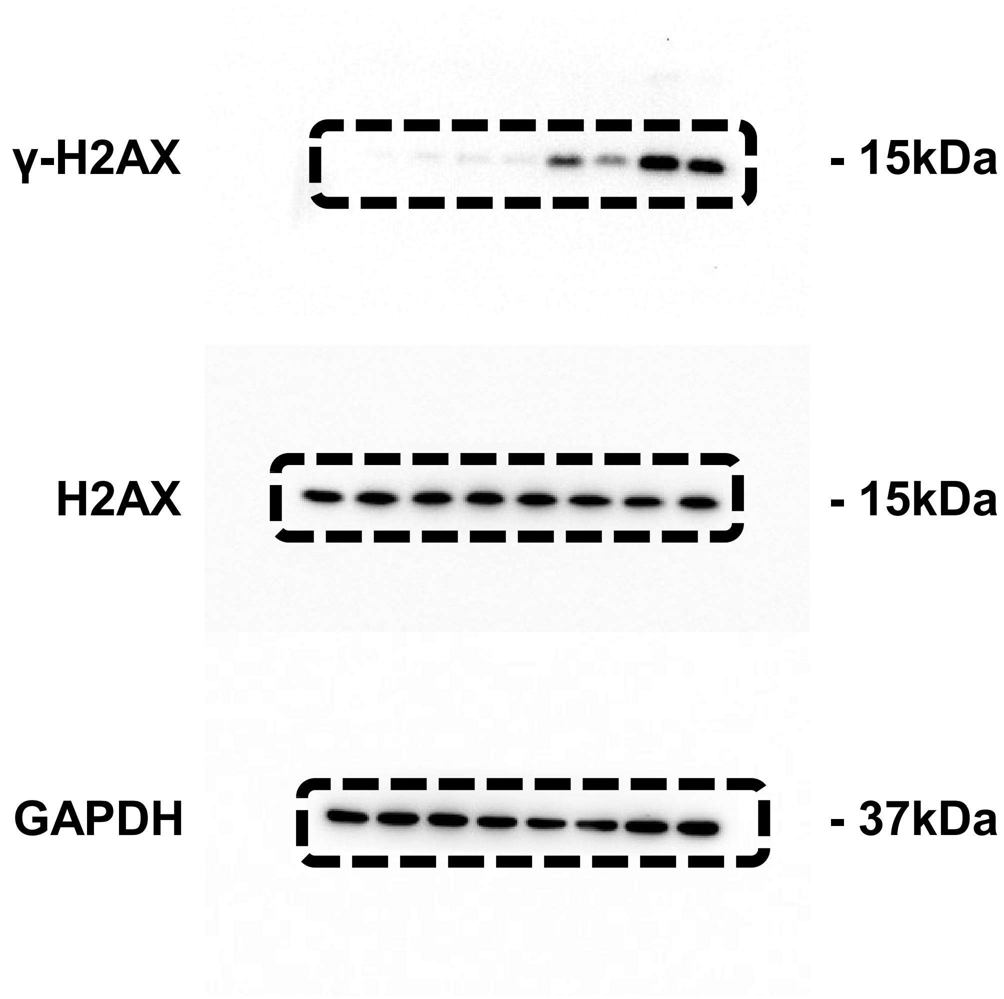

Figure4 C

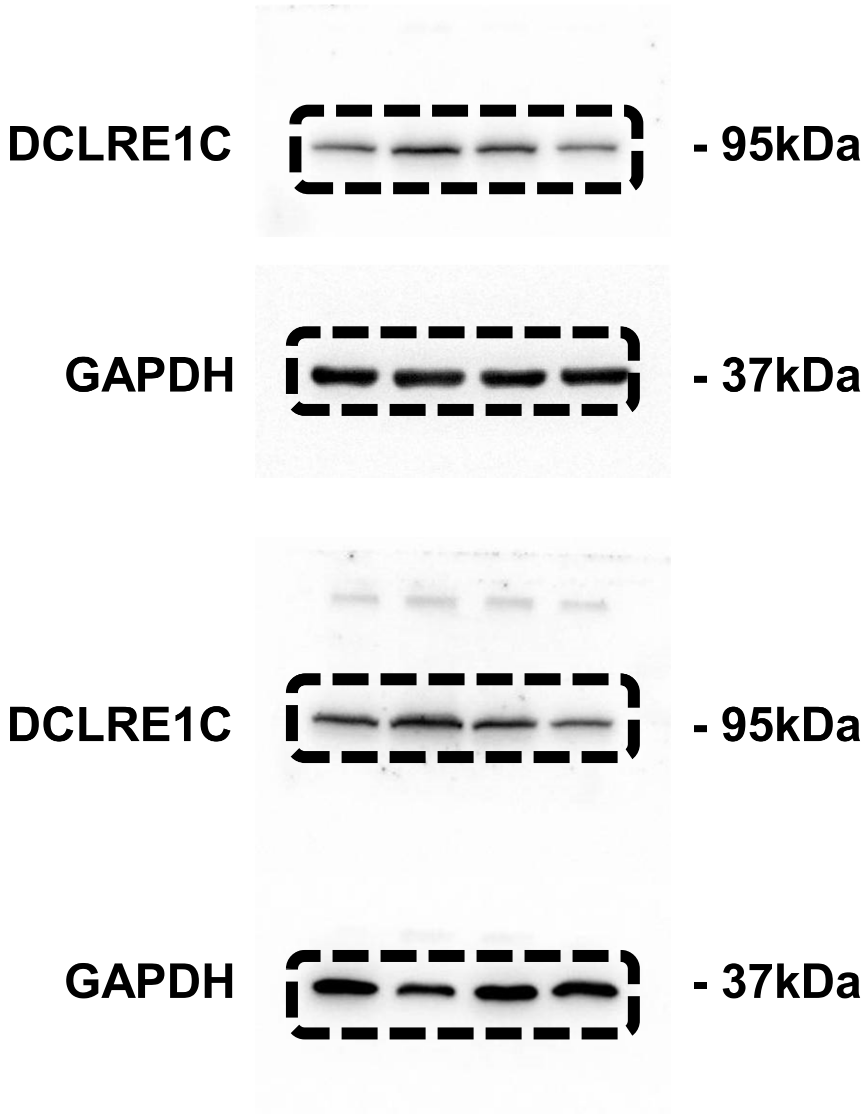

Figure4 G

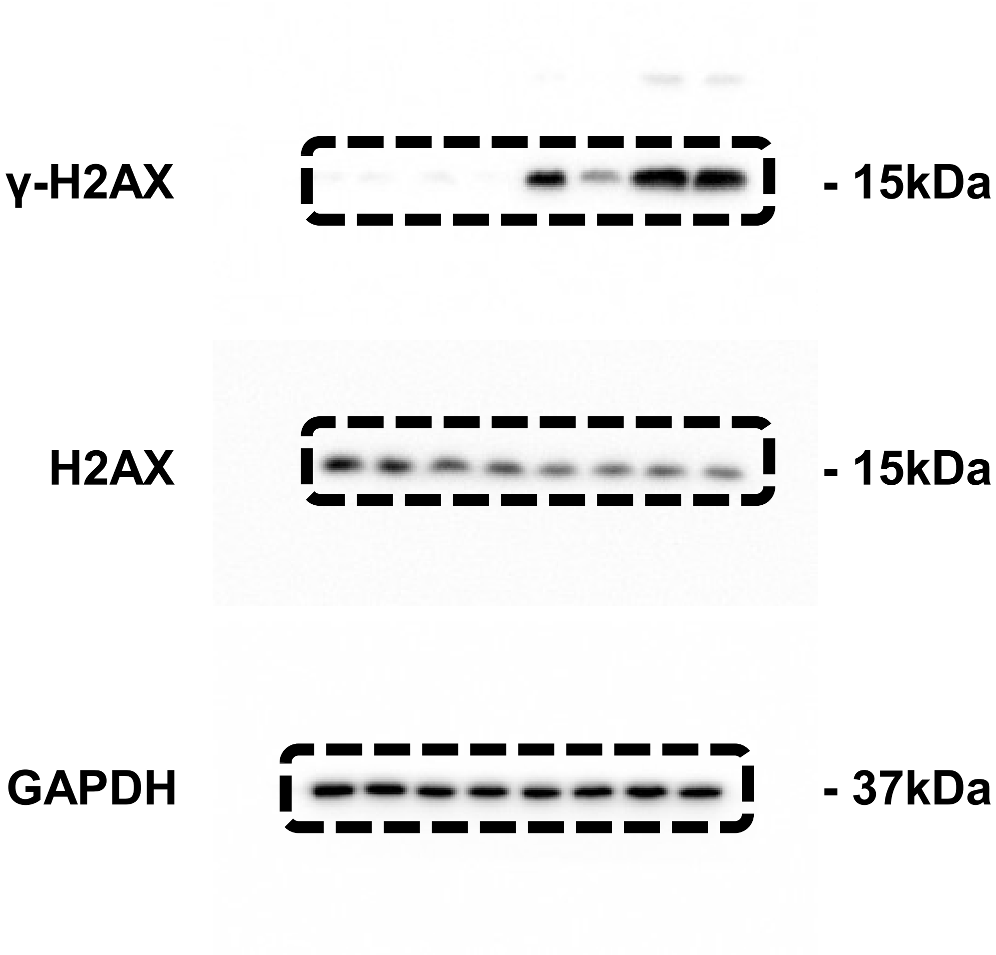

Figure5 E

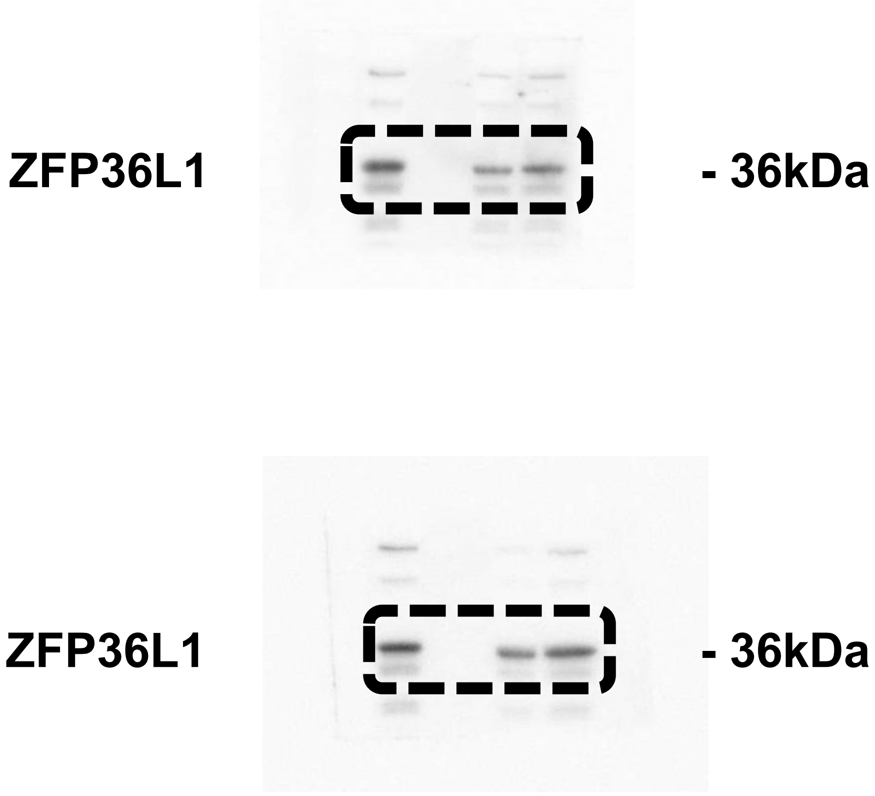

Figure6 D

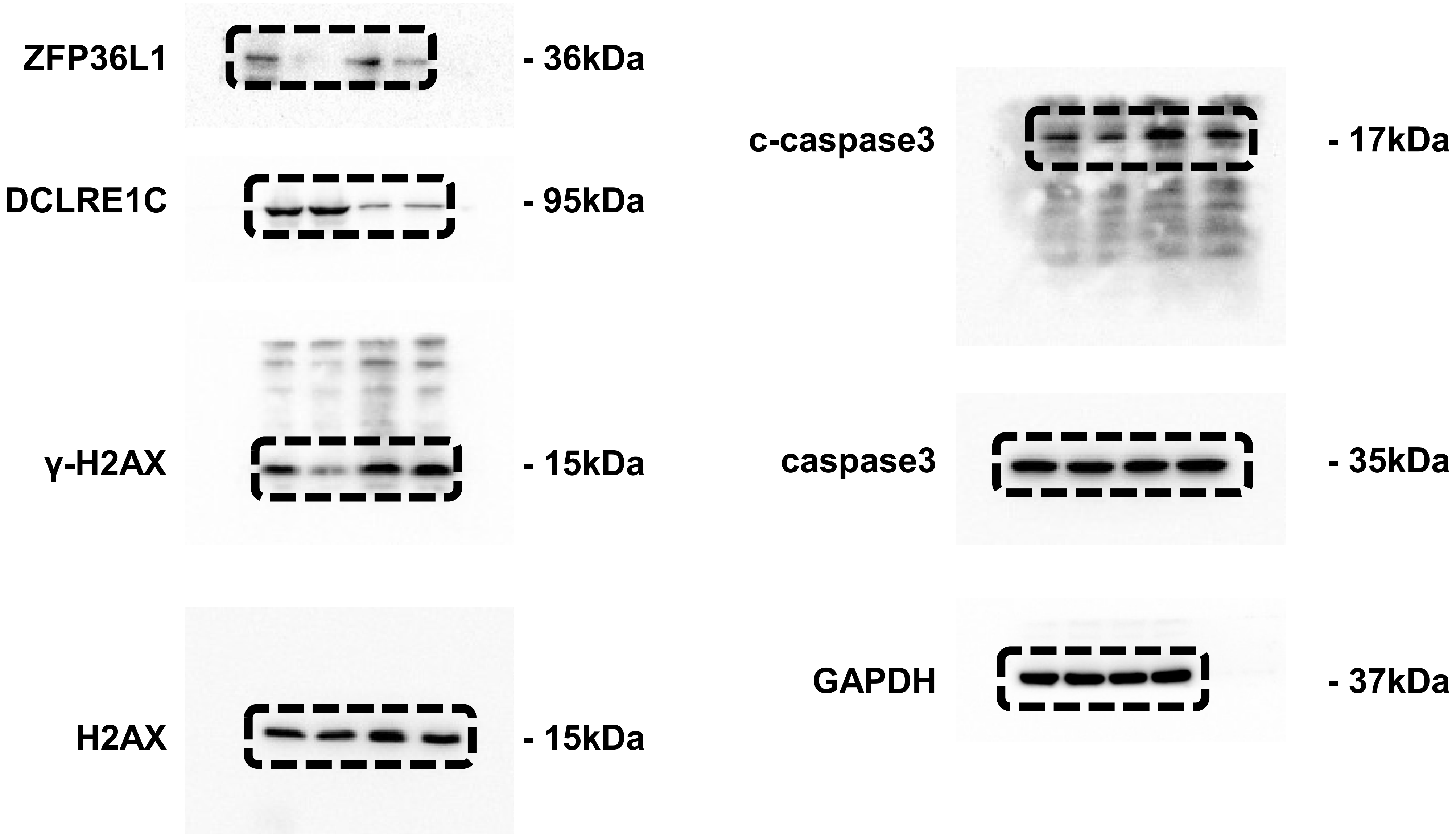
ZFP36L1
- 36kDa
DCLRE1C
- 95kDa
γ-H2AX
- 15kDa
H2AX
- 15kDa
c-caspase3
- 17kDa
caspase3
- 35kDa
GAPDH
- 37kDa

Supplementary Figure1 E

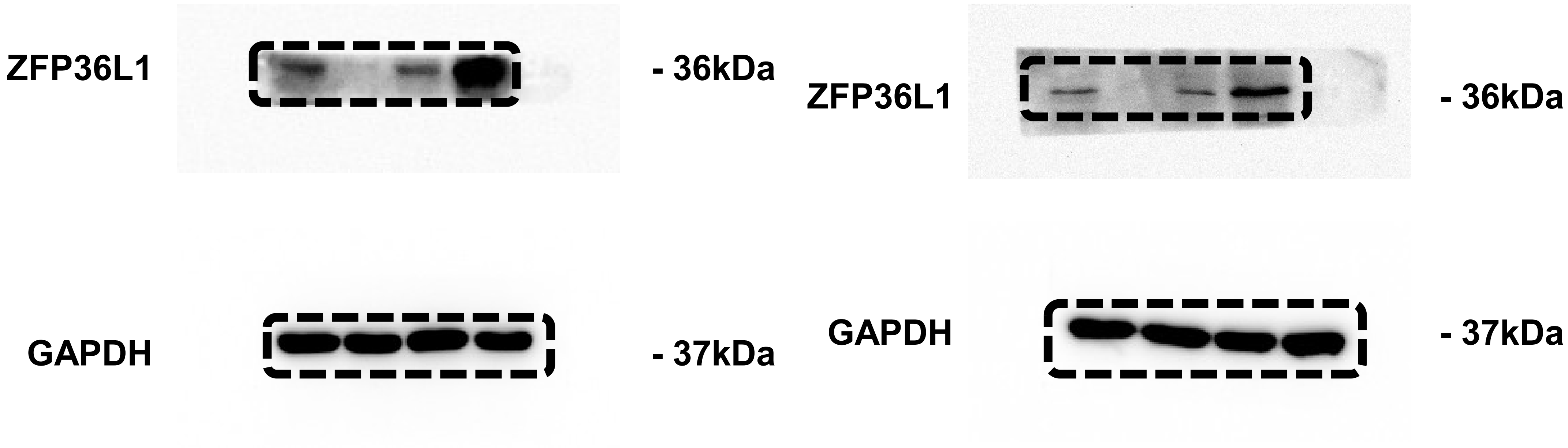

Supplementary Figure3 D

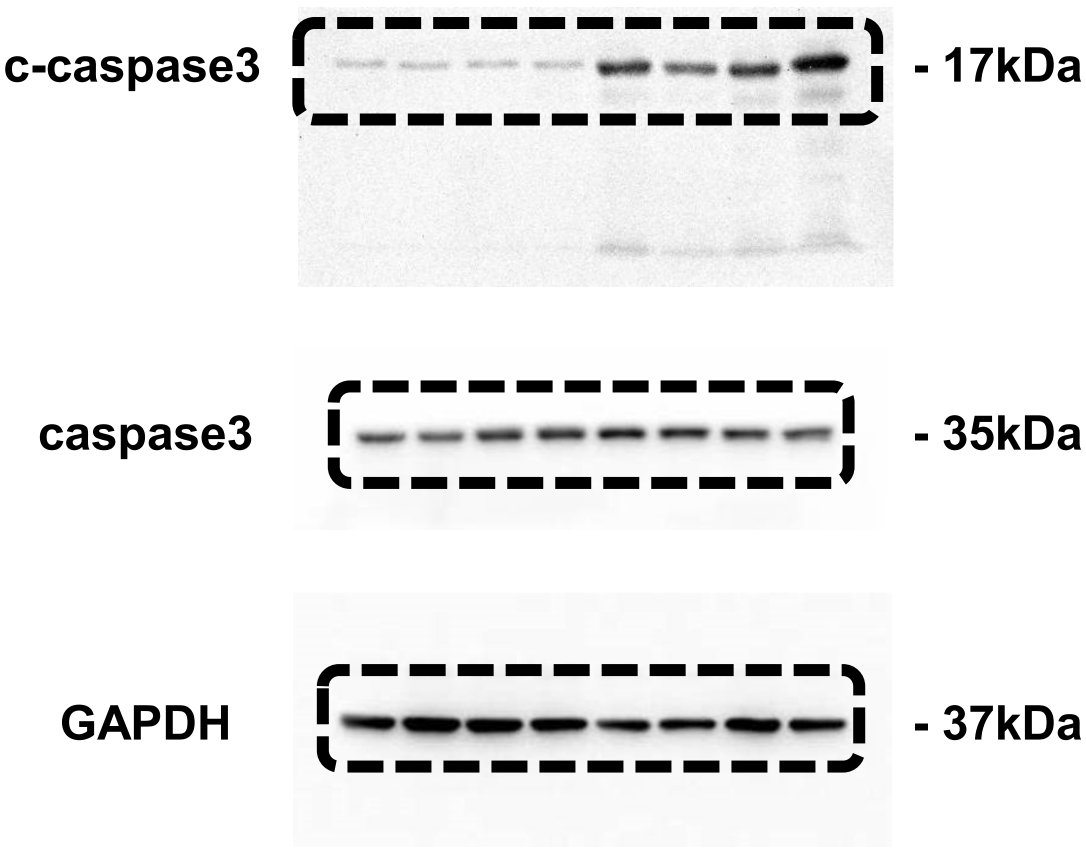

Supplementary Figure4 F

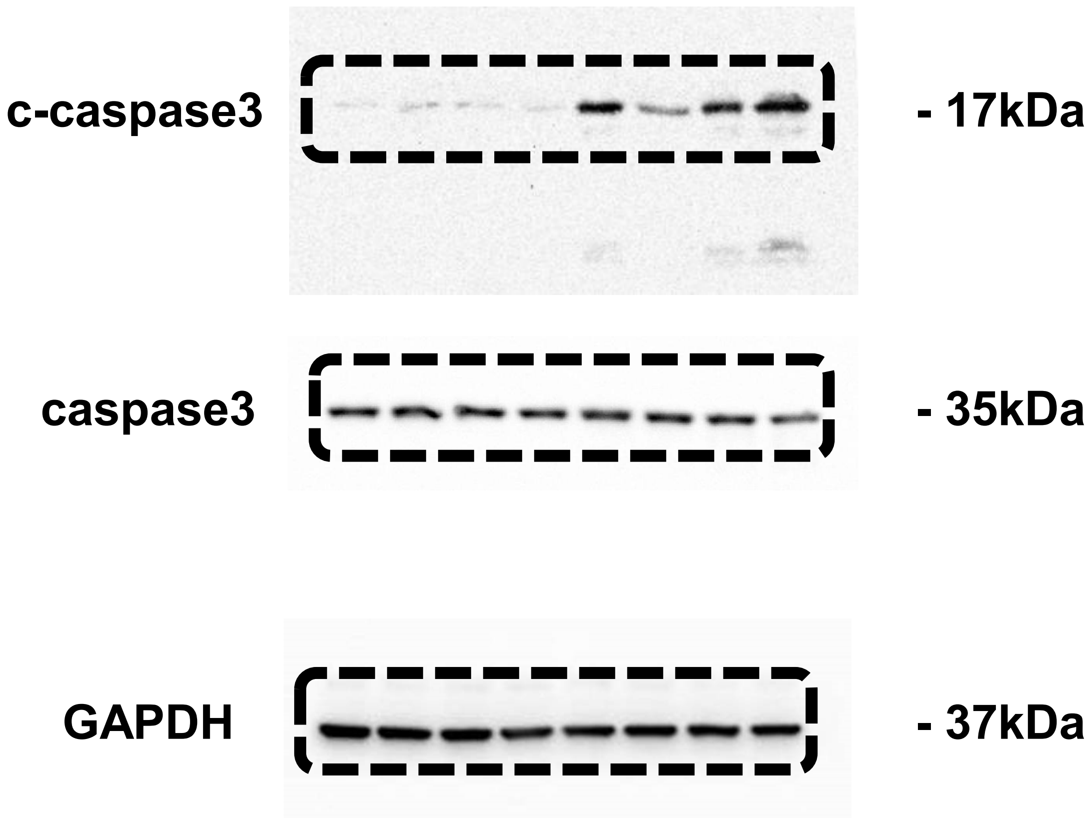

Supplementary Figure5 C

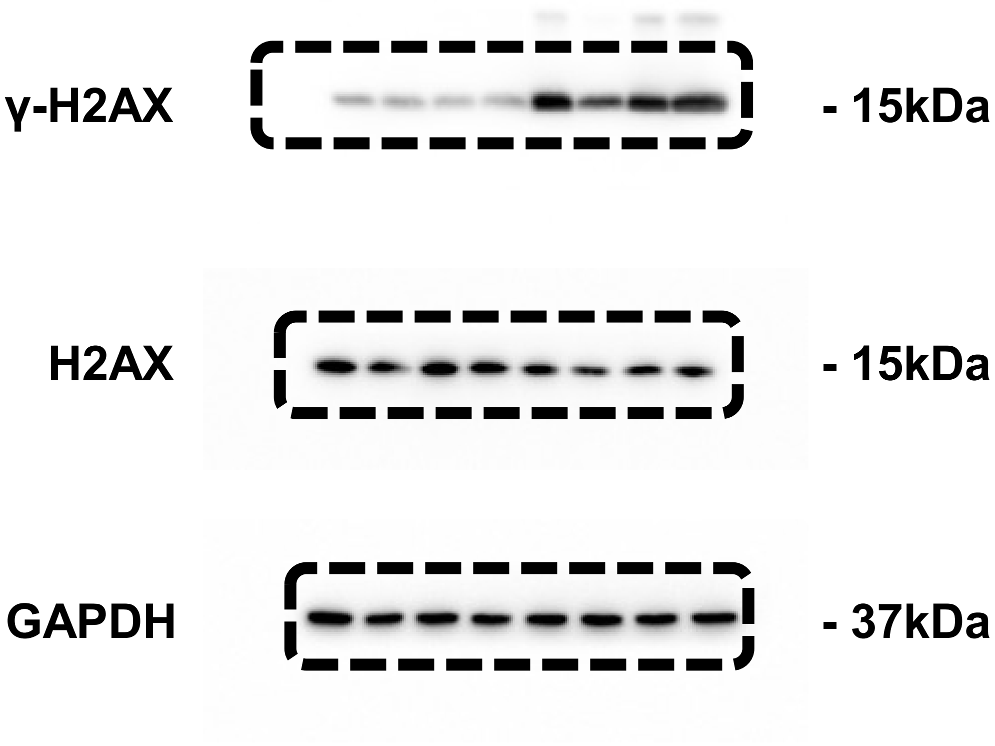

Supplementary Figure6 B

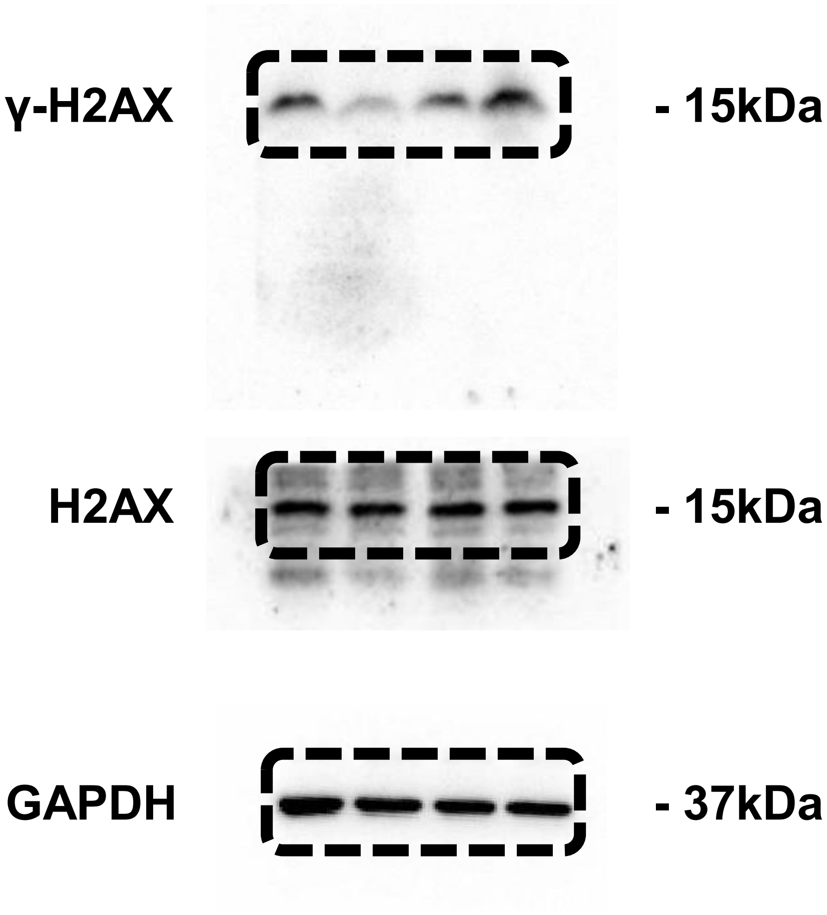

Supplementary Figure6 B

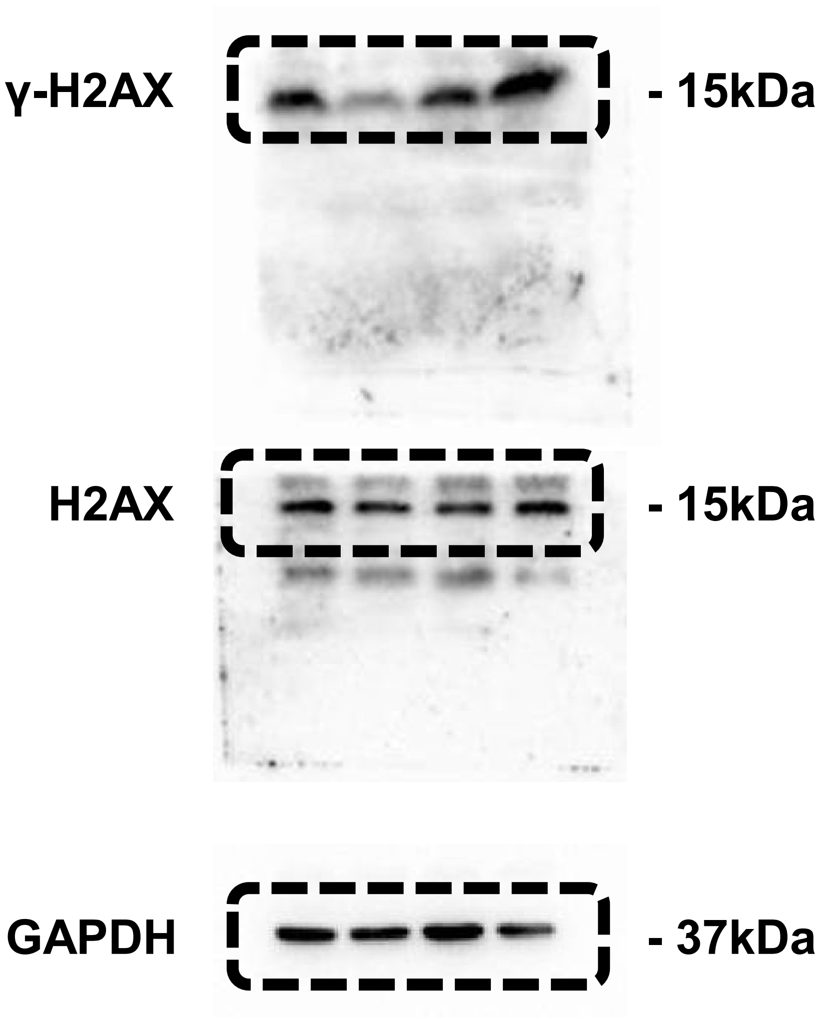

Supplementary Figure7 D

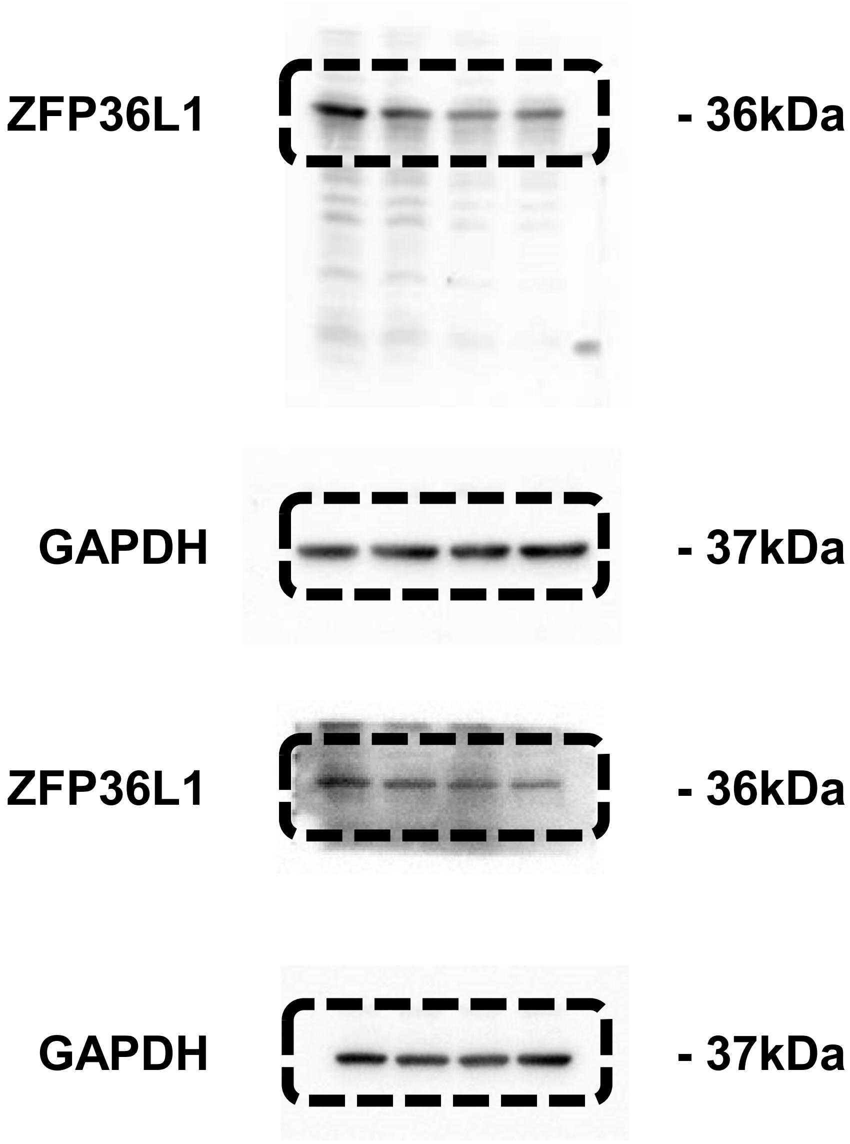

Supplementary Figure9 C

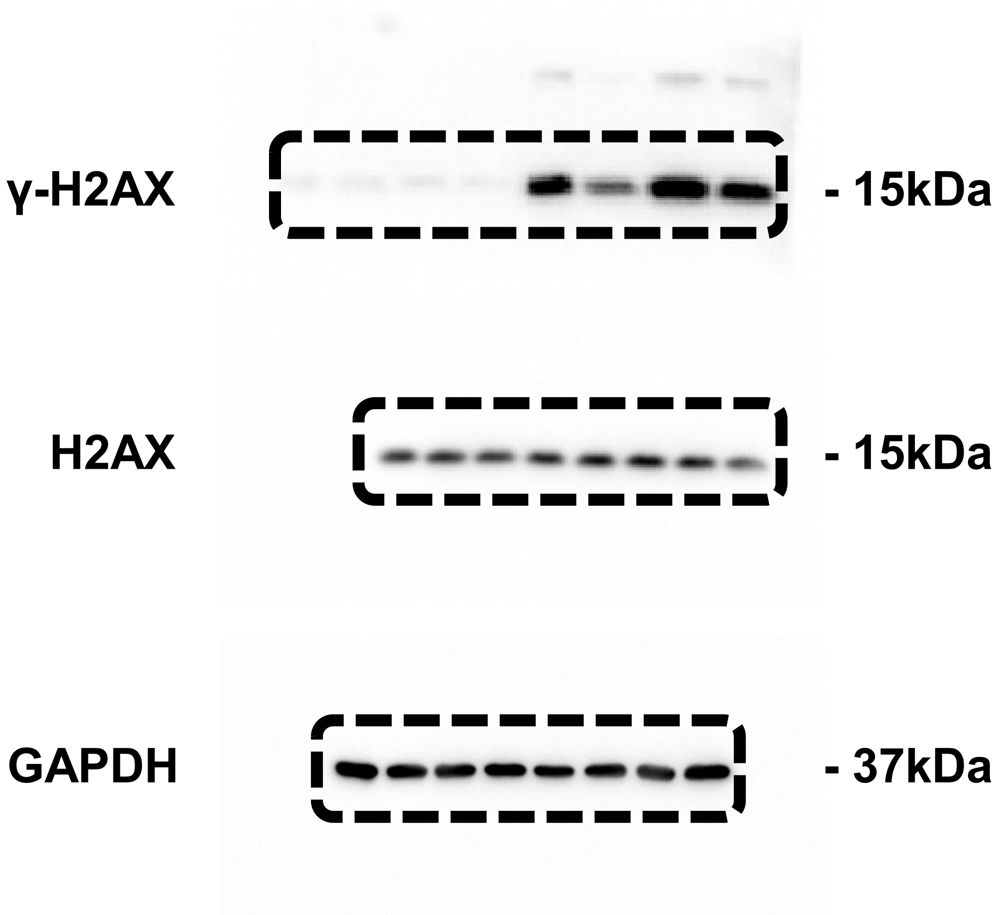

Supplementary Figure11 C

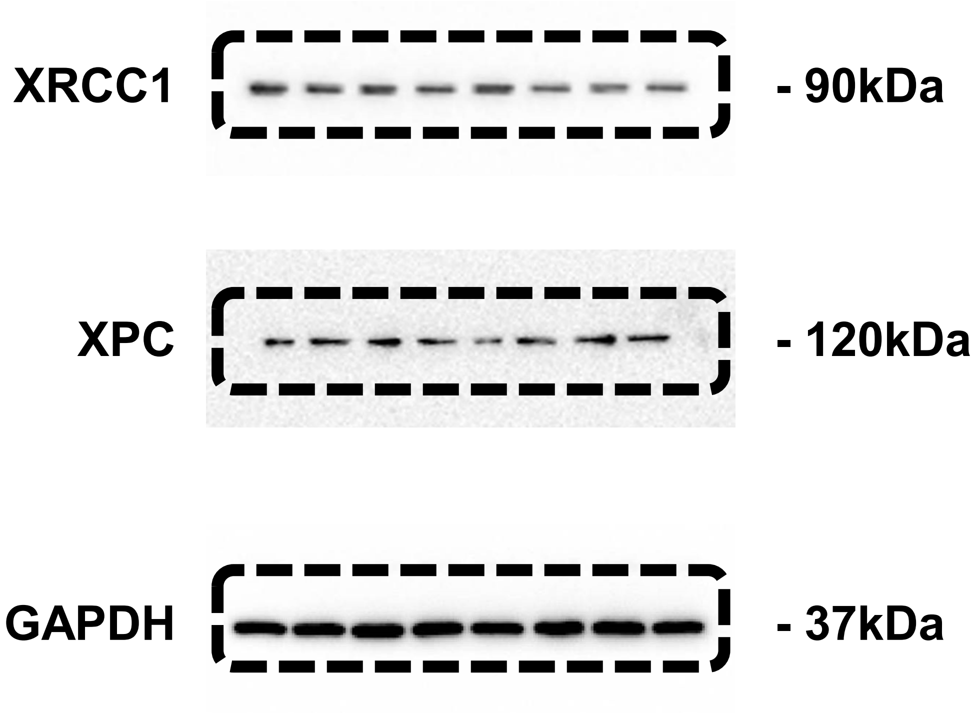

Supplementary Figure12 A

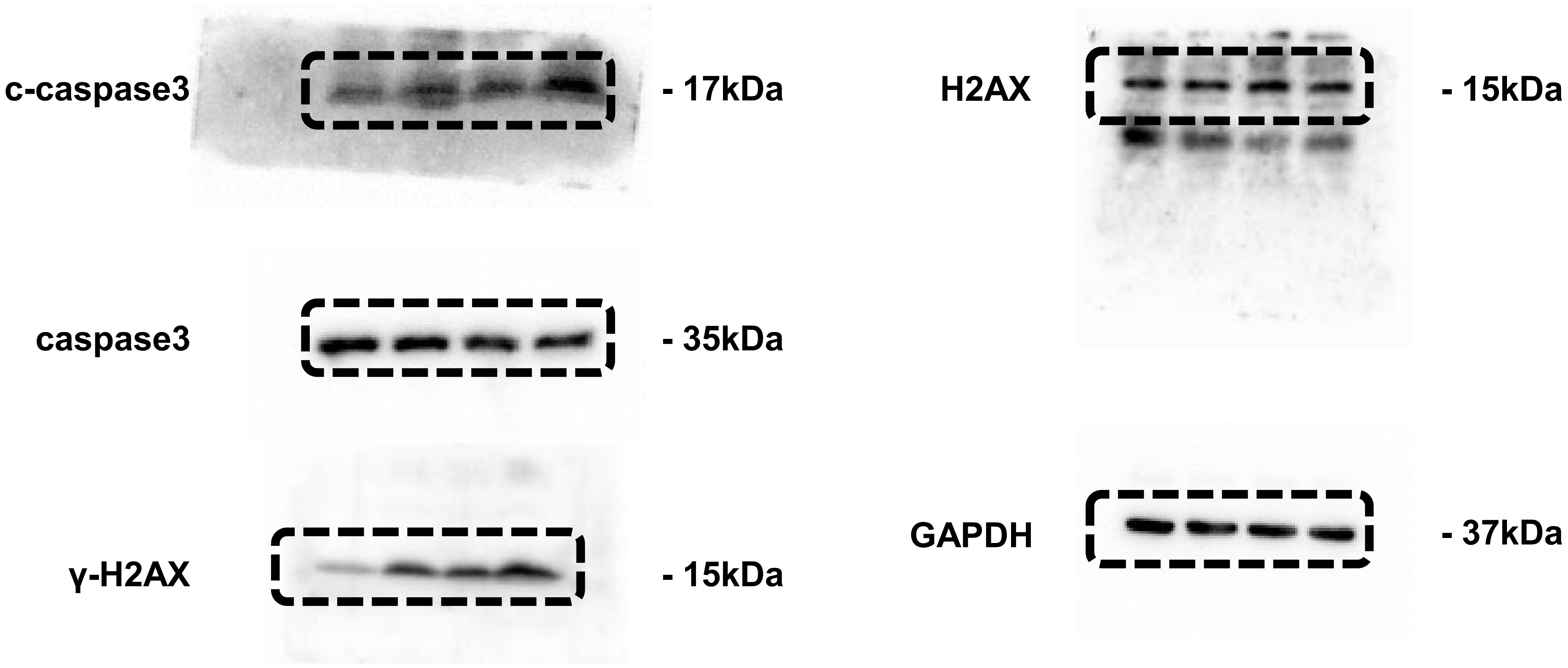

Supplementary Figure14 B

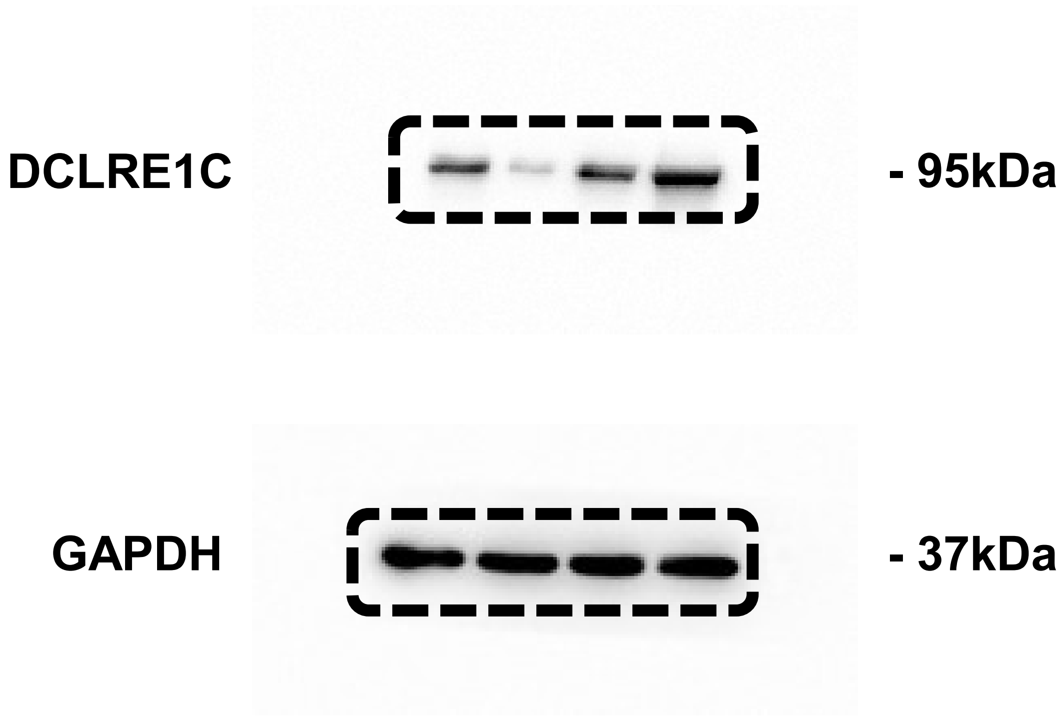

Supplementary Figure14 E

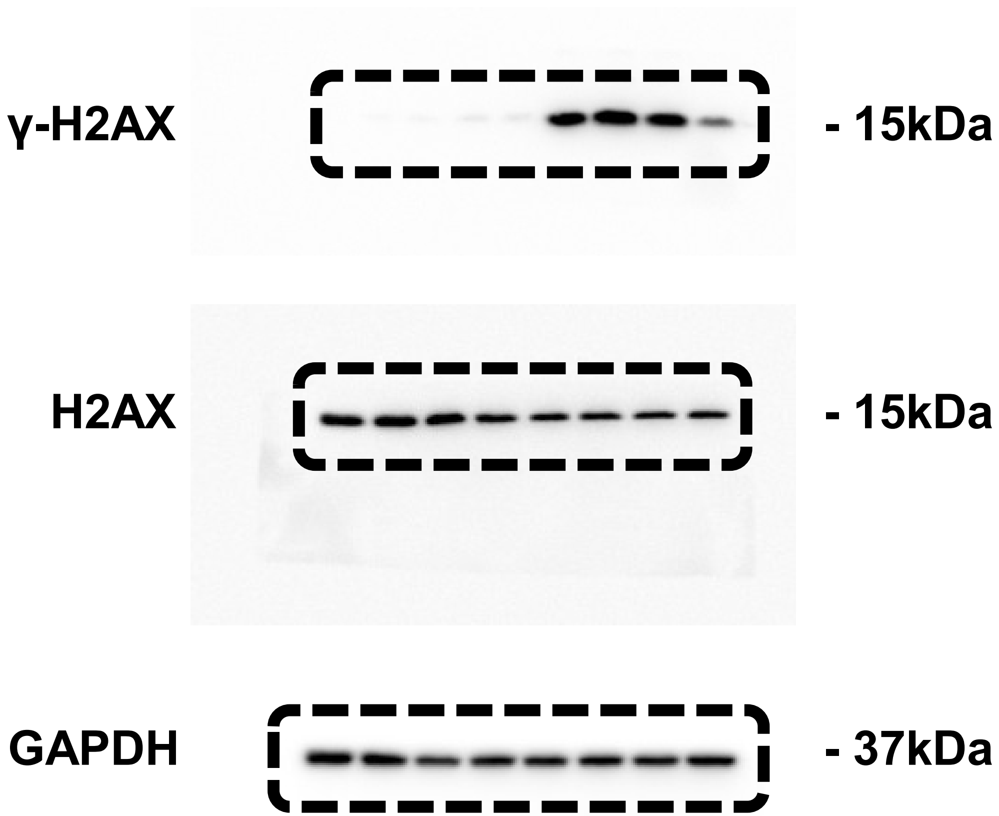

Supplementary Figure15 A

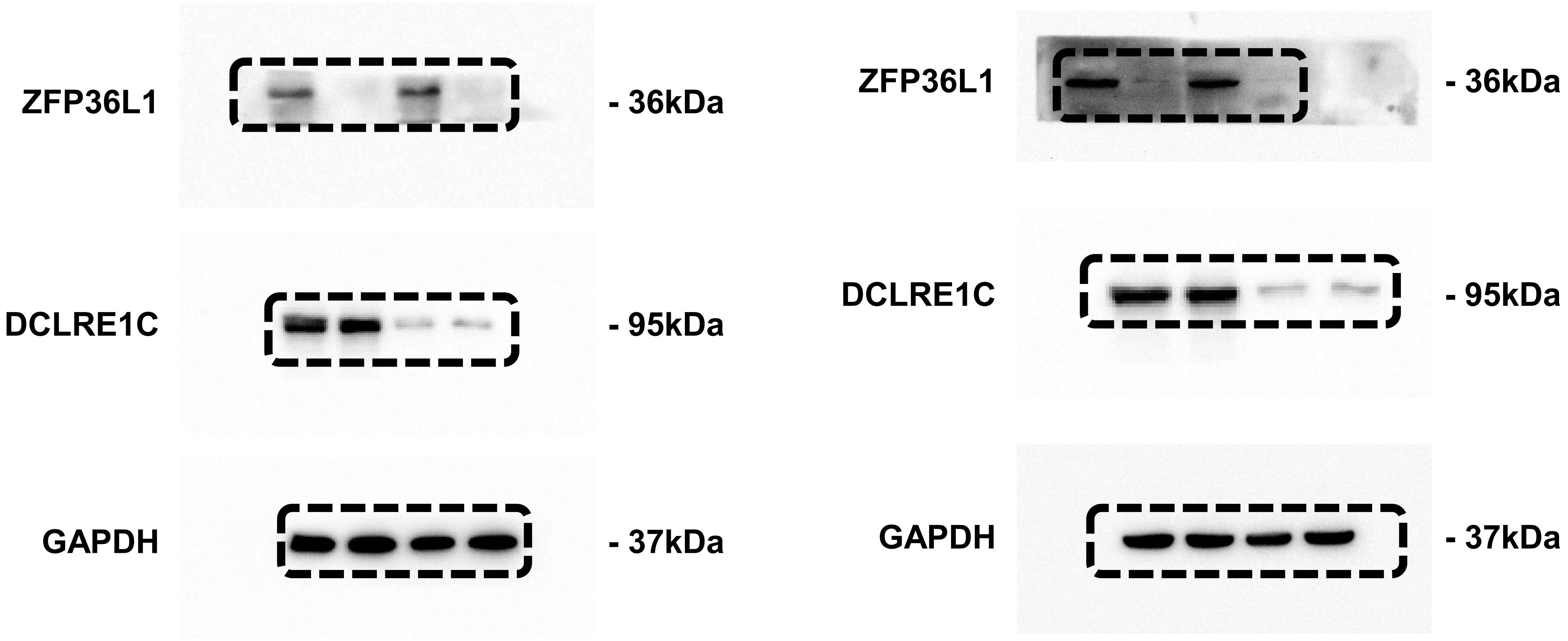

Supplementary Figure15 C

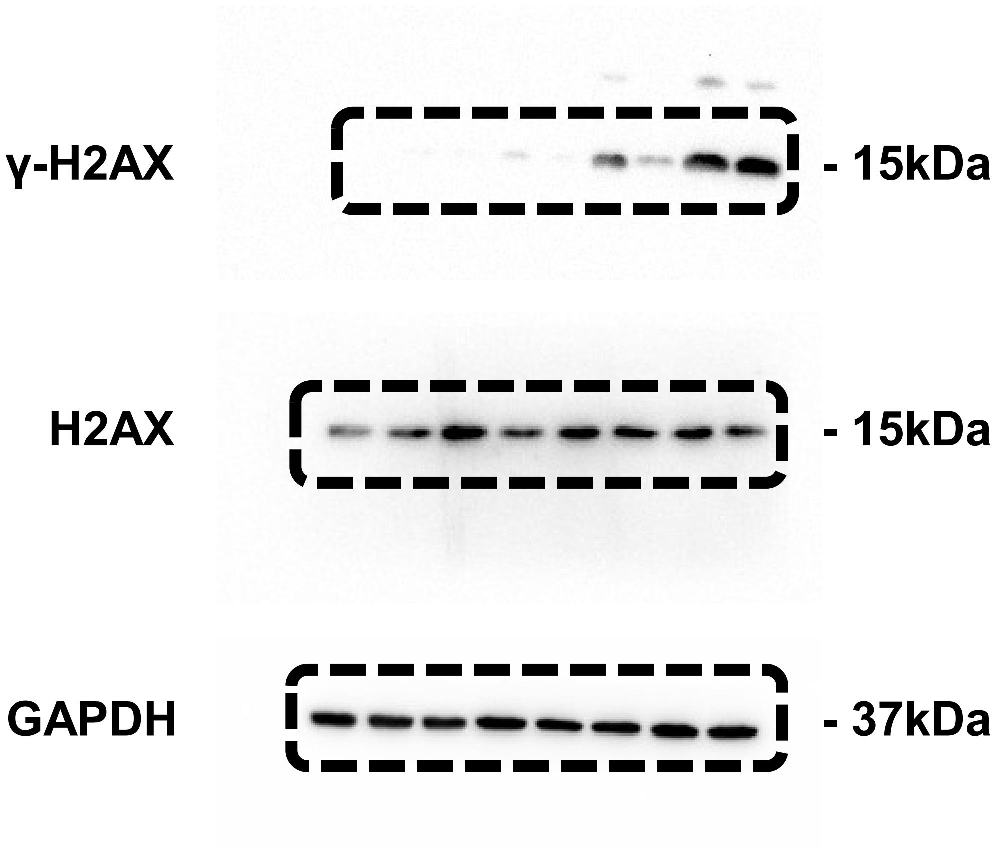

Supplementary Figure16 B

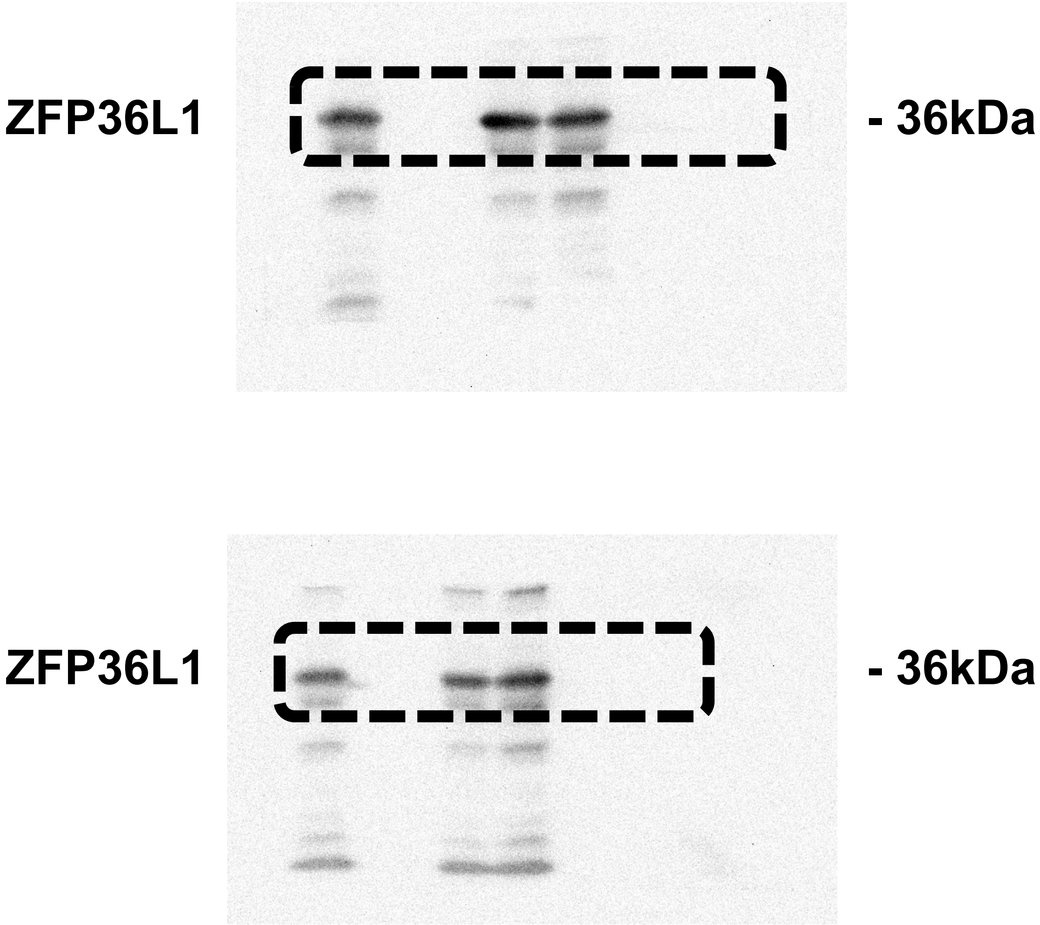

Supplementary Figure16 D

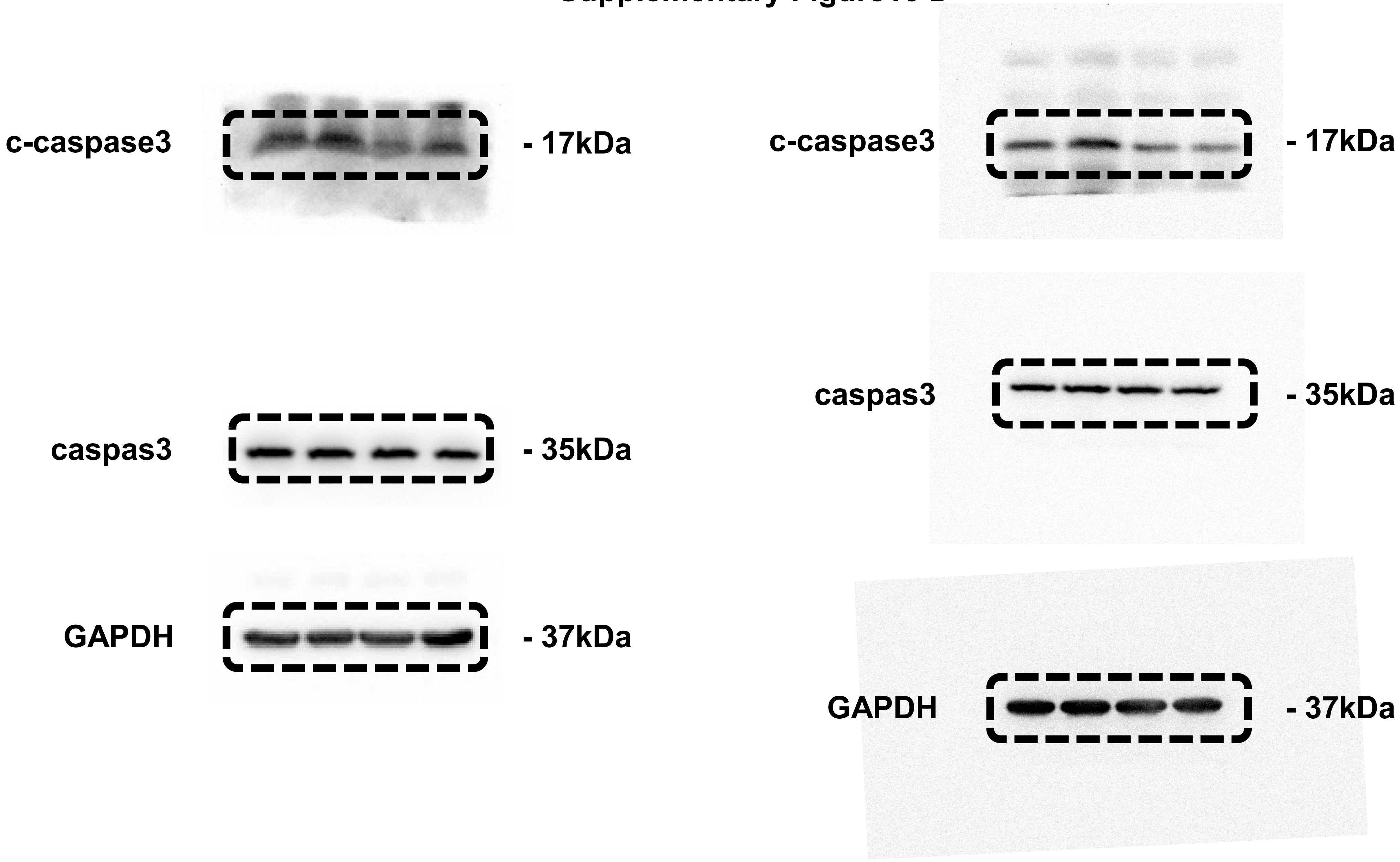

Supplementary Figure17 A

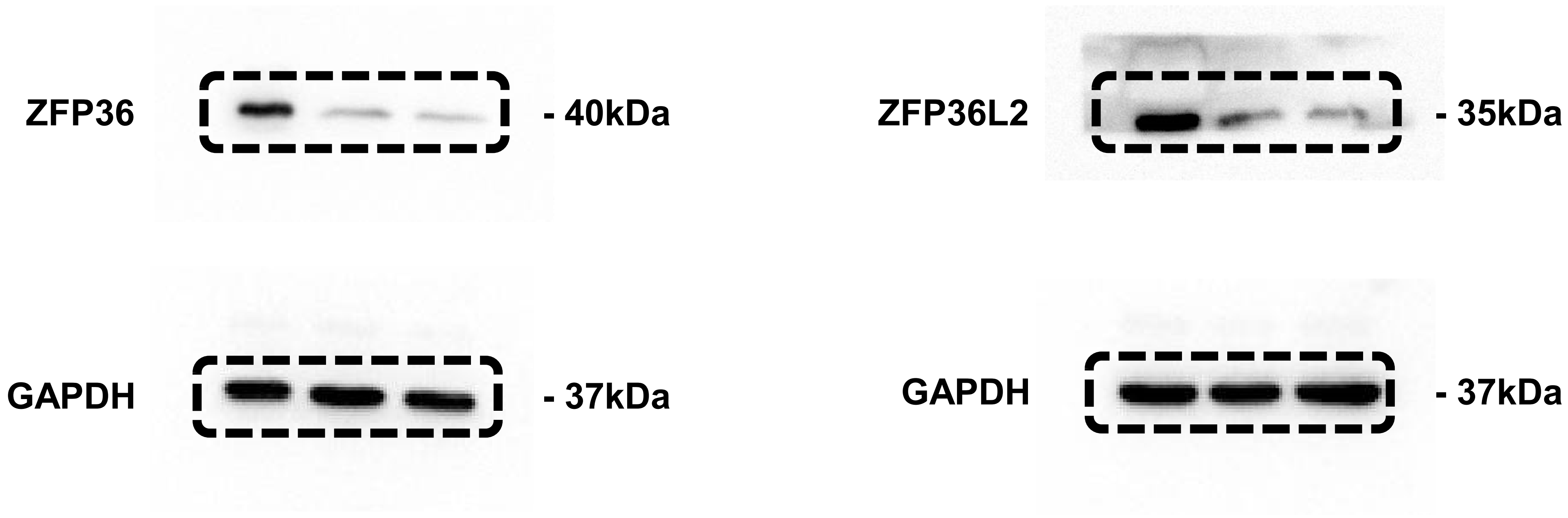

Supplementary Figure17 B

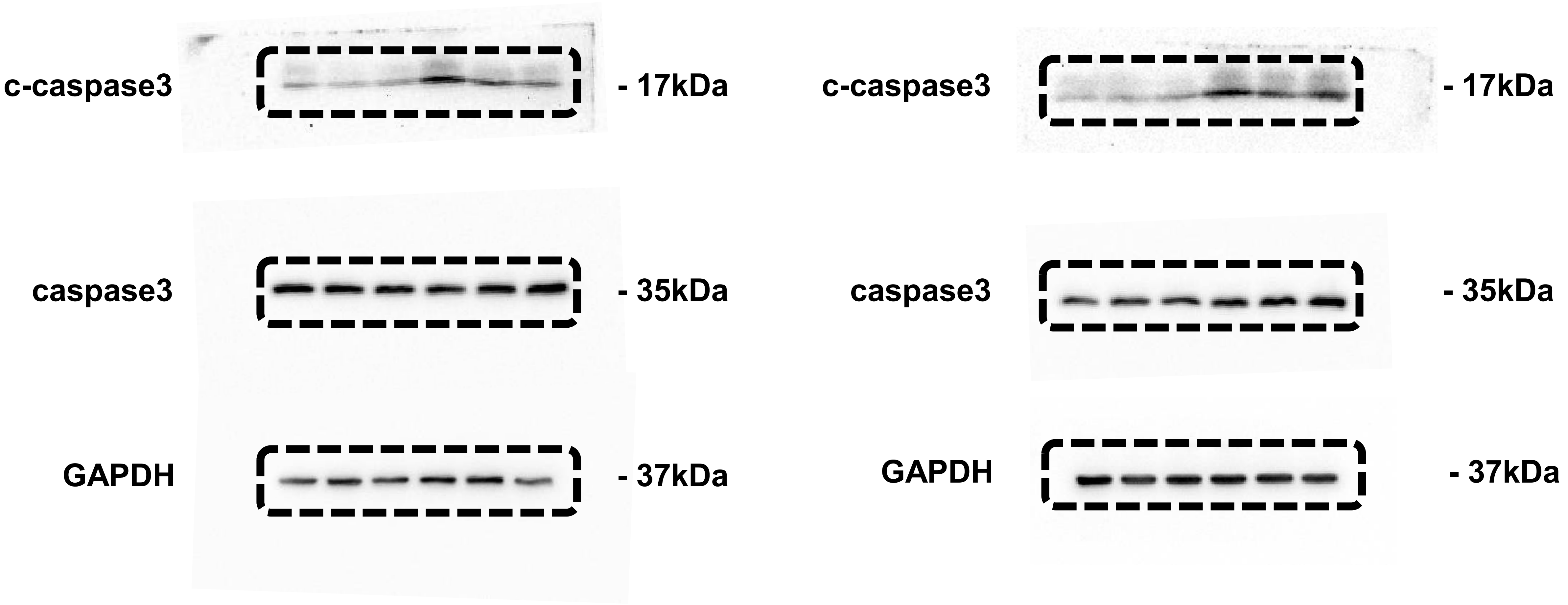

Supplementary Figure17 D

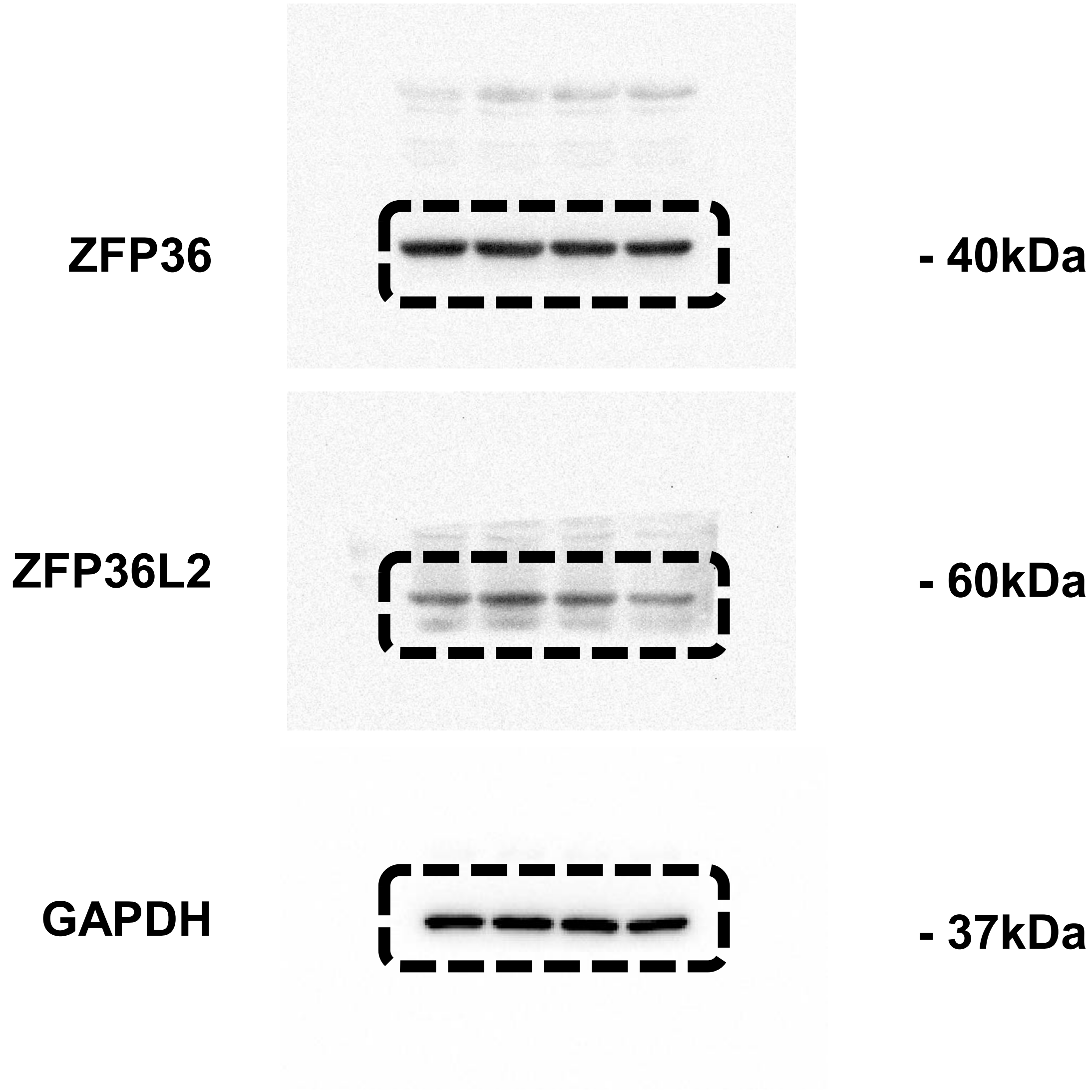

Supplementary Figure17 E

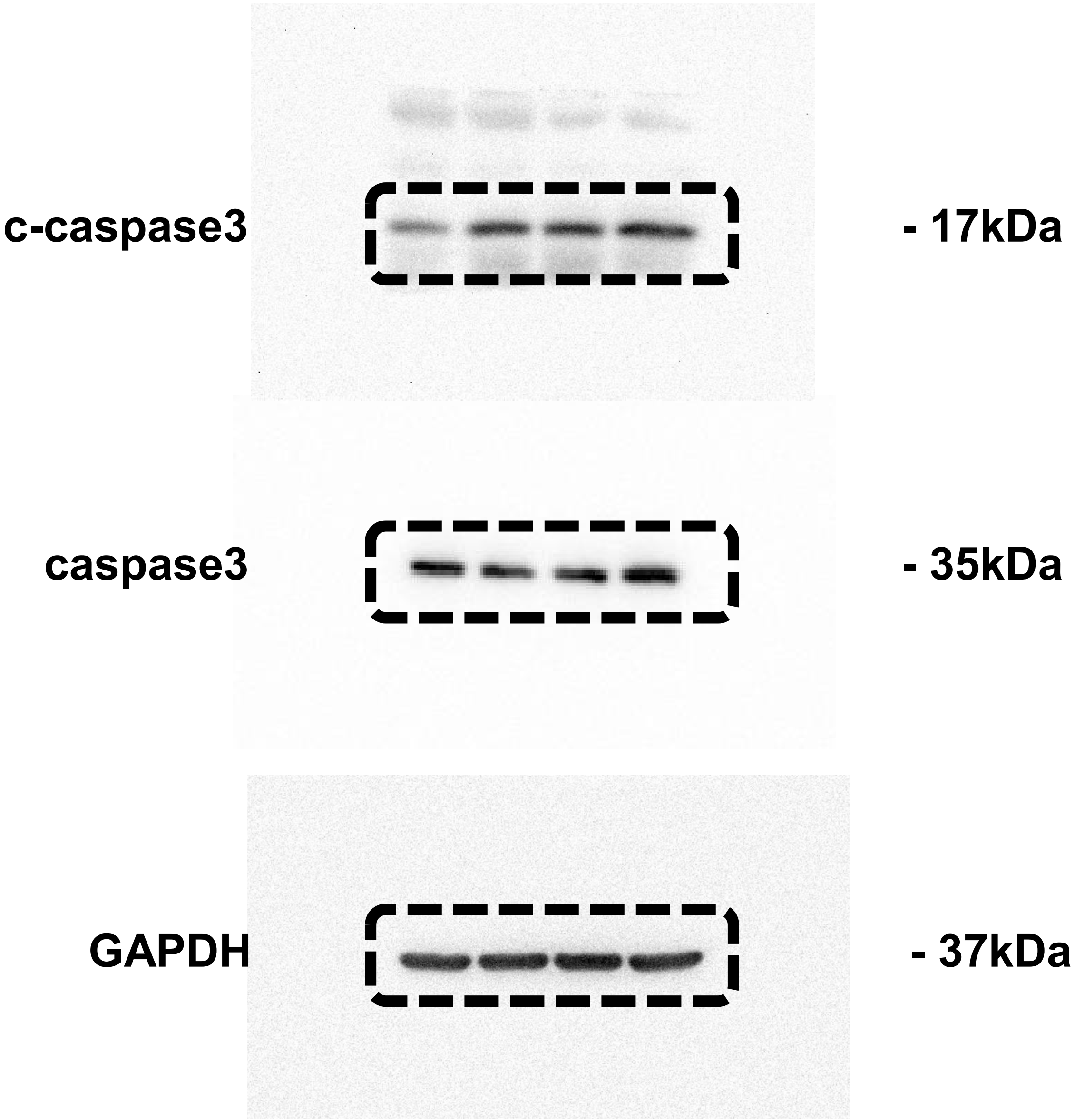

Supplement: Supplementary file 3 — Original data [file 41419_2025_8217_MOESM3_ESM.pdf]
